# Overconsumption of low-digestible sugars can increase susceptibility to *Salmonella* Typhimurium infection

**DOI:** 10.1038/s41467-026-78218-0

**Published:** 2026-11-03

**Authors:** Bidong D. Nguyen, Sandra Y. Wotzka, Lisa Maier, Céline Fetz, Yves Steiger, Sanne Kroon, Elena F. Verdu, Nermin Akduman, Markus Arnoldini, Dorothée L. Berthold, Annika Hausmann, Camille Schmid, Patrick Kiefer, Philipp Christen, Manja Barthel, Lea Fuchs, Andreas Sichert, Ersin Gül, Emma Slack, Julia A. Vorholt, Wolf-Dietrich Hardt

**Affiliations:** 1 Institute of Microbiology, Department of Biology, ETH Zurich, Zurich, Switzerland; 2 Cluster of Excellence EXC 2124 Controlling Microbes to Fight Infections, Interfaculty Institute for Microbiology and Infection Medicine Tübingen, M3-Research Center for Malignome, Metabolome and Microbiome, University of Tübingen, Tübingen, Germany; 3 Farncombe Family Digestive Health Research Institute, Department of Medicine, McMaster University, Hamilton, Canada; 4 Institute for Food, Nutrition and Health, Department of Health Sciences and Technology, ETH Zürich, Zürich, Switzerland; 5 Institute of Molecular Systems Biology, Department of Biology, ETH Zürich, Zürich, Switzerland

**Keywords:** Pathogens, Microbiome

## Abstract

Low-digestible carbohydrates (LDCs), including dietary fibers and sugars that resist absorption, are generally considered beneficial for the gut microbiota. However, we show that acute overconsumption of certain LDCs, particularly low-digestible sugars (LDSs), such as arabinose, lactulose, and polyols commonly used as sugar substitutes, can compromise colonization resistance against the common foodborne pathogen *Salmonella enterica* serovar Typhimurium. LDS overconsumption results in elevated fecal pathogen loads, systemic dissemination, and colitis in infected mice. Mechanistic analysis of arabinose overconsumption revealed that rapid fermentation acidifies the intestinal environment, perturbs the microbiota, alters gut-luminal short-chain fatty acid levels and elevated amino acid availability, which can support pathogen growth. Additionally, this perturbation impacts virulence by broadening tissue tropism (e.g. expansion to small intestine) and selecting for invasive *Salmonella*. Unlike antibiotic perturbations, *Salmonella* colonization requires SPI-1 and SPI-2 Type-III-secretion systems, linking virulence to pathogen fitness. These findings reveal how dietary perturbations can impact the gut ecosystem in ways that not only compromise colonization resistance but also enhance pathogen virulence.

## Abstract (web summary)

Here, the authors show that acute overconsumption of certain poorly absorbed sugars, including some sugar substitutes, can disrupt the gut microbiota, weaken resistance to *Salmonella* colonization, and enhance pathogen virulence.

## Introduction

The gut microbiota plays a vital role in human health, supporting metabolism, immunity, and pathogen defense^1–3^. Dysbiosis, an imbalance in the microbial community, has been linked to metabolic disorders, chronic inflammatory diseases, and infections^4–7^. Diet is a key factor in microbiota homeostasis, as it directly influences microbial composition and functionality^8^. A critical component of the diet is low-digestible carbohydrates (LDCs), which include dietary fibers and resistant sugars that bypass absorption in the small intestine, becoming accessible for fermentation by the microbiota^9^. High-fiber diets increase microbial diversity and support fiber-degrading bacteria, such as Bacteroides, whereas low-fiber Western diets are associated with a higher abundance of Firmicutes and Proteobacteria^10–13^. Western diets are further linked to a higher risk of metabolic and cardiovascular diseases, as well as an increased prevalence of inflammatory bowel disease (IBD) and other gut-related disorders, in part due to a dysbiotic microbiota^14,15^. LDCs exert prebiotic effects by serving as substrates for microbial fermentation, resulting in the production of short-chain fatty acids (SCFAs), which play a crucial role in host metabolism, physiology, and immune regulation^16,17^. Diets low in LDCs reduce SCFA production, contributing to barrier dysfunction, bacterial overgrowth, and pro-inflammatory states^18^.

As LDCs promote the growth of beneficial microbiota, they also influence colonization resistance (CR), the microbiota’s ability to block enteric pathogens^6,19^. CR is conferred through competition for nutritional and physical niches, as well as microbiota-mediated immune defenses^20^. LDCs can accelerate the resolution of *Clostridioides difficile* infection^21^, whereas high-fat, low-fiber diets exacerbate disease severity through the depletion of *Lachnospiraceae* and *Ruminococcaceae*, key competitors for amino acids required by *C. difficile*^22^. However, LDCs can also have divergent effects. While inulin promotes beneficial microbes, such as *Bifidobacterium* and *Faecalibacterium prausnitzii*^23^, it can also promote *Citrobacter* growth in mice fed a Western-style diet^24^. Likewise, lactulose and fructooligosaccharides have been reported to enhance *Salmonella enterica* colonization and invasiveness^25^ though the basis for these effects is not well understood.

Given these findings, we tested additional dietary compounds for their ability to increase susceptibility to *S. enterica* serovar Typhimurium (*S*.Tm) infection in mice. This screen revealed that acute overconsumption of simple LDCs (termed LDSs), including sugar alcohols (polyols), arabinose, and lactulose, compromises CR and promotes colitis with marked systemic dissemination. We focused on arabinose to investigate mechanisms by which certain LDSs can increase susceptibility to enteric infection. Arabinose overconsumption was accompanied by reduced intestinal pH through microbiota fermentation, microflora perturbation and pathogen colonization. Arabinose intake was also associated with enhanced SPI-1 expression, broader tissue tropism (e.g., increased translocation into Peyer’s patches and ileal tissues), and positive selection for virulence. Collectively, these findings demonstrate that dietary perturbations can enhance pathogen virulence.

## Results

### Acute overconsumption of several LDSs supports *S*.Tm colonization in the gut

**Fig. 1:**
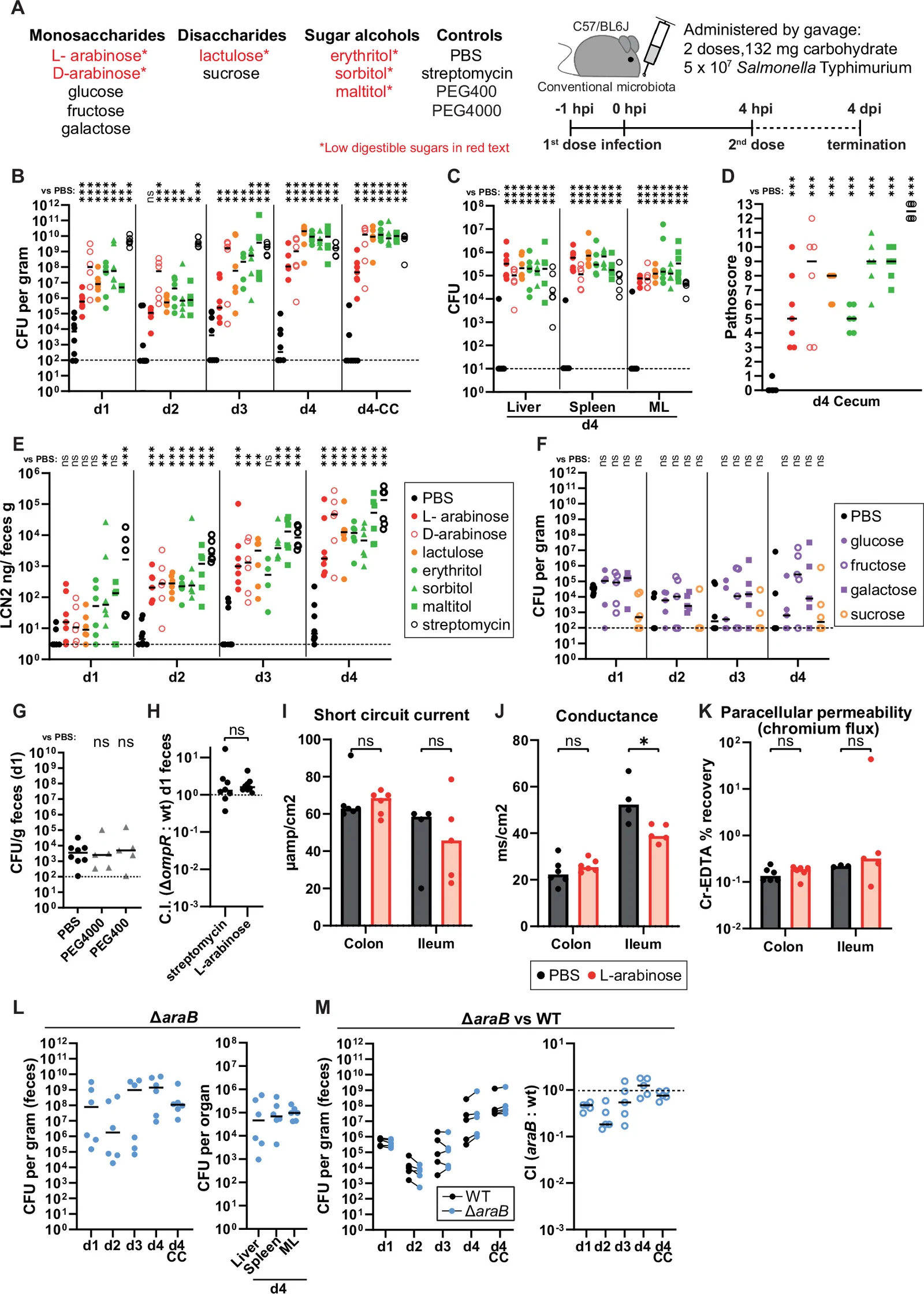
Acute overconsumption of low-digestible sugars promotes S.Tm blooms and pathogen-induced colitis. **A** Experimental setup. SPF C57BL/6 J mice received two 132 mg doses of the indicated carbohydrate or controls by oral gavage, one hour before and four hours after oral infection with 5 × 10^7^ *S*.Tm. Streptomycin-pretreated mice were given 25 mg of the antibiotic by gavage one day prior to infection. LDSs are indicated in red text. **B–E** Effects of LDS overfeeding on *S*.Tm colonization. *n* = 8 mice for PBS, 7 for L-arabinose, and 6 each for D-arabinose, lactulose, erythritol, sorbitol, maltitol, and streptomycin. Pathogen loads in **B** feces collected on days 1–4 (d1–d4) post infection (p.i.), as well as cecum contents (CC) and **C** internal organs (liver, spleen, and mesenteric lymph nodes (ML)) at the termination of the experiment on day 4 p.i. Gut inflammation was assessed by **D** histopathology of cecum tissue (taken at d4 p.i.) and **E** fecal Lipocalin-2 levels by ELISA. **F** Pathogen fecal loads of mice receiving HDCs. *n* = 6 mice for PBS, 7 and 5 each for glucose, fructose, galactose, and sucrose. **G** Fecal loads on day 1 of mice given two doses of PBS, 132 mg PEG4000 or 132 mg PEG400 (*n* = 8, 5, and 5 mice, respectively). **H** Competitive index of Δ*ompR* mutant against a wild type strain in mice given streptomycin or arabinose. *n* = 8 mice per group. **I–K** Ussing chamber measurements of **I** short circuit current, **J** conductance and **K** paracellular permeability, as assessed by serosal ^51^Cr-EDTA recovery, of ileum and cecum from mice receiving either PBS or two doses of 132 mg of arabinose (*n* = 6 mice per group). **L**,** M** Mice were given two doses of 132 mg arabinose and **L** orally infected with either 5 × 10^7^ CFU of the Δ*araB* mutant strain alone (*n* = 6 mice) or **M** a 1:1 mixture of wild-type and Δ*araB* strains at a total dose of 5 × 10^7^ CFU (*n* = 5 mice); (**M**, left) colony-forming units per gram in feces and cecum contents; (**M**, right) the competitive index (CI), expressed as the ratio of the mutant to wild-type strains. **B–K** The two-tailed Mann-Whitney U test was used for statistical analysis; *P < 0.05; **P < 0.01; ***P < 0.001. At least two independent experiments were performed. Horizontal lines indicate medians. Abbreviation: hpi hours post infection; dpi days post infection; ns not significant. The source data and *P* values for panels **B–M** are provided in the Source Data file.

The genomes of *Salmonella* spp. encode a broad repertoire of carbohydrate utilization pathways, enabling growth on at least 73 different compounds as sole carbon sources^26–28^. Despite this versatility, *Salmonella* still competes with the resident microbiota for these nutrients and is forced to rely on less preferred carbon sources^29,30^. To determine whether increased carbohydrate availability promotes *Salmonella* growth, we screened a panel of digestible and low-digestible carbohydrates for their impact on *S*.Tm growth in vivo (Fig. 1A). C57BL/6 mice harboring a specified pathogen-free complex (SPF) microbiota with pronounced colonization resistance were gavaged twice with carbohydrate solutions (132 mg per dose) and infected between doses (Fig. 1A). Carbohydrate amounts exceeded the typical daily intake of fermentable short-chain carbohydrates in mice fed on standard chow (16–184 mg/day, assuming 3 g food intake^31^). Mice receiving LDSs (arabinose, lactulose, and sugar alcohols) excreted significantly higher *S*.Tm fecal loads, more than 100-fold above the PBS controls on day 1 post-infection (p.i.) (Fig. 1B). While fecal *S*.Tm loads declined by day 2 p.i., a second *S*.Tm bloom occurred, reaching levels comparable to streptomycin-pretreated control mice (10^7^–10^11^ CFU/g of feces) by day 3–4 p.i. (Fig. 1B^32^). LDS-treated mice also developed systemic infections, with elevated *S*.Tm counts detected in the liver, spleen, and mesenteric lymph nodes (Fig. 1C). These mice also had colitis as indicated by the inflammatory marker lipocalin in feces (10^2^–10^5^ ng/g) by days 2–4 and histopathological analysis at day 4 p.i. (Fig. 1D, E). These values exceeded those in PBS controls, but inflammation remained less pronounced than in streptomycin-pretreated mice. These findings suggest that LDSs enhanced *S*.Tm colonization and systemic dissemination while inflammatory responses remained milder than in antibiotic-pretreated mice.

In contrast to LDSs, high-digestible carbohydrates (HDCs) had little effect on infection. Fructose, glucose, galactose, and sucrose did not significantly alter pathogen fecal excretion compared to PBS controls (Fig. 1F and Supplementary Fig. 1A), likely because these are efficiently absorbed in the small intestine^33^, and therefore do not reach the lower gastrointestinal tract, the primary site of *S*.Tm growth and disease in mice^32,34^.

High carbohydrate doses could induce osmotic stress, potentially disrupting the microbiota and promoting *Salmonella* growth. However, the administration of osmotically active compounds PEG400 or PEG4000 did not increase fecal *Salmonella* loads relative to PBS control mice (Fig. 1G). Additionally, the osmotic-sensitive *ompR* mutant showed no growth attenuation compared to wild-type in arabinose-fed or streptomycin-pretreated mice (Fig. 1H). Intestinal barrier function remained intact in mice that ingested arabinose, as confirmed by Ussing chamber tests assessing ion transport and permeability across the intestinal mucosa for 5 out of 6 parameters. (Fig. 1I–K). In the absence of infection, LDS overconsumption did not alter tissue histopathology, immune cell populations and inflammatory gene expression (Supplementary Fig. 1B–D). These control experiments rule out osmotic stress, barrier dysfunction, and histopathological damage as major contributors to *S*.Tm overgrowth in LDS-overfed mice.

Among the compounds tested, only LDSs enhanced *S*.Tm infection. Of these, we chose L-arabinose for further investigation because it had only a modest effect on initial gut colonization yet markedly promoted systemic infection and salmonellosis.

### L-arabinose utilization by *S*.Tm is not required for gut colonization

Since arabinose cannot be absorbed by the host, it remains in the gut lumen after ingestion^35^. Therefore, *S*.Tm may consume the supplemented arabinose to fuel its growth. *S*.Tm possesses the required genes for arabinose uptake and catabolism (*araABCD araE*) and can use arabinose as a sole carbon source^28,36,37^. However, several observations indicate that *S*.Tm arabinose utilization is dispensable for infection. The D-enantiomer of arabinose, not metabolizable by *S*.Tm^38^, produced similar effects to those of L-arabinose (Fig. 1B). Furthermore, the *ΔaraB* mutant, which cannot catabolize L-arabinose, still established gut colonization and caused systemic infection in mice that ingested L-arabinose (Fig. 1L). In co-infection experiments, the *ΔaraB* mutant showed a slight growth defect only during the first two days of infection (Fig. 1M). This indicates that while arabinose utilization provides some minor fitness advantage, arabinose overconsumption promotes pathogen expansion indirectly, likely through effects on the microbiota that alter the availability of other nutrients.

### Without infection, acute overconsumption of arabinose transiently alters the microbiota composition

**Fig. 2:**
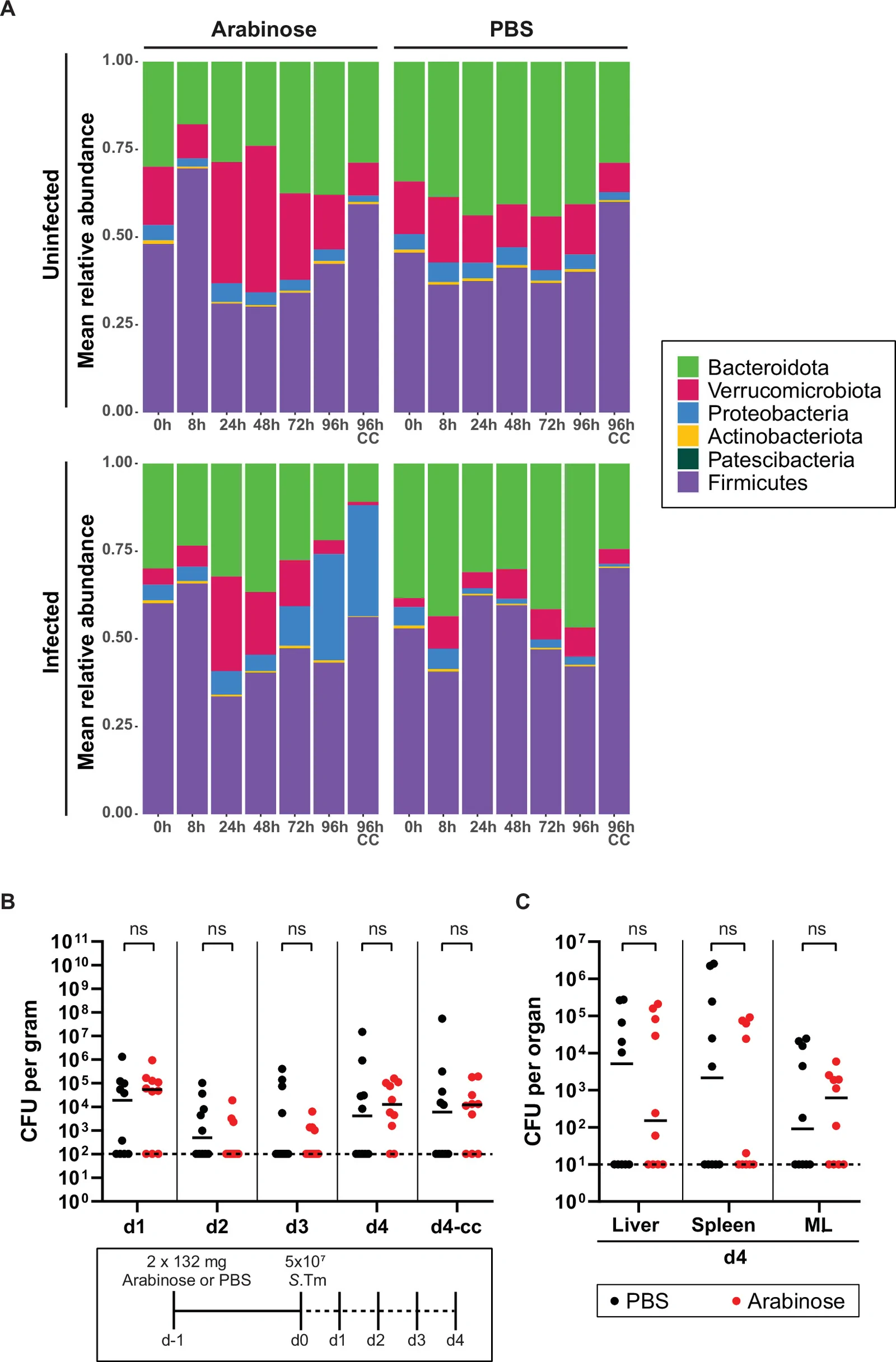
Acute overconsumption of arabinose transiently alters microbiota composition. **A** 16S rDNA analysis of fecal microbiota composition in uninfected or infected mice administered two doses of 132 mg arabinose or mock-treated with PBS. Shown is the mean composition (*n* = 6 mice per group). **B**,** C** Mice were given two doses of 132 mg arabinose or PBS one day prior to oral infection with 5 × 10^7^ CFU total *S*.Tm; *n* = 10 mice per group. **B** Pathogen loads in feces (d1–d4) and cecal contents (CC) and **C** in systemic organs (liver, spleen, and mesenteric lymph nodes (ML)). At least two independent experiments were performed. The two-tailed Mann–Whitney *U* test was used for statistical analysis; ns, not significant. Horizontal lines indicate medians. The source data for panels **A–C** are provided in the Source Data file.

We hypothesized that arabinose overconsumption perturbs the gut microbiota and thereby promotes infection. 16S rRNA gene sequencing revealed transient microbiota shifts that return to the pre-ingestion baseline within 96 h in uninfected mice (Fig. 2A). Eight hours post-arabinose administration, Bacteroidota abundance decreased while Bacillota (f. Firmicutes) increased. By 24 h, Bacteroidota levels recovered, Bacillota declined, Verrucomicrobiota increased before all changes resolved by 96 h (Fig. 2A). Delaying infection until one day after arabinose ingestion abolished *S*.Tm colonization, indicating that restoration of colonization resistance parallels microbiota recovery (Fig. 2B, C). The shifts were specific to arabinose overconsumption, as PBS-treated controls exhibited relatively stable microbiota composition.

In infected arabinose-overfed mice, Verrucomicrobiota gradually declined while *S*.Tm (Proteobacteria) increased, consistent with overall rising pathogen fecal CFUs (Fig. 2A and Supplementary Fig. 2A). This suggests that arabinose overconsumption opens a niche exploitable by *S*.Tm, possibly at the expense of Verrucomicrobiota. By days 3 and 4 p.i. major compositional shifts emerged, including a marked decline in both Bacteroidota and Verrucomicrobiota, coinciding with inflammation onset and luminal *S* Tm densities of 10^7^–10^10^ CFUs per gram stool (Fig. 2A and Supplementary Fig. 2A, B). In contrast, mice infected with avirulent Δ*invG* or Δ*ssaV* mutants, which cannot trigger inflammation, maintained microbiota compositions similar to uninfected, arabinose-overconsuming mice, with communities trending back toward baseline and an absence of *S*.Tm growth (Supplementary Fig. 2C). These data suggest that luminal pathogen blooms at days 3–4 p.i. in arabinose-overconsuming mice require virulence-triggered inflammation to overcome colonization resistance from the renormalizing microbiota.

Taken together, these observations explain the infection kinetics (Fig. 1B). Arabinose overconsumption shifts microbiota composition, permitting an initial pathogen bloom on day 1 p.i. that collapses by day 2 p.i. *Salmonella* virulence subsequently drives more drastic microbiota changes, resulting in a robust *S*.Tm bloom on days 3–4 p.i. (Fig. 2A and Supplementary Fig. 2A, B**)**.

### Fermentation of arabinose by the microbiota leads to rapid intestinal acidification that subsequently reduces colonization resistance

The mechanisms underlying the microbial shifts are not immediately clear, as arabinose is neither inherently toxic nor limited to a narrow subset of microbial taxa. As a component of plant cell walls, arabinose serves as a carbon source for various gut bacteria involved in the digestion and fermentation of dietary fibers^39,40^. Arabinose utilization operons are widespread across taxa, including Enterobacteriaceae, Bacteroidota, Bifidobacteriaceae, Enterococcceae, and Oscillospiraceae^41–43^. To test whether representative gut bacteria can metabolize arabinose, we grew selected human gut isolates on L-arabinose. Arabinose supplementation enhanced the growth of *Bacteroides vulgatus*, *B. uniformis*, *B. thetaiotaomicron*, *B. ovatus*, *Roseburia intestinalis*, and *Dorea* spp., but not *Clostridium ramosum* and *C. perfringens*, *Lactobacillus paracasei*, and *Streptococcus parasanguinis* (Supplementary Fig. 3A**)**. Medium acidification was observed in strains that fermented arabinose or glucose.

**Fig. 3:**
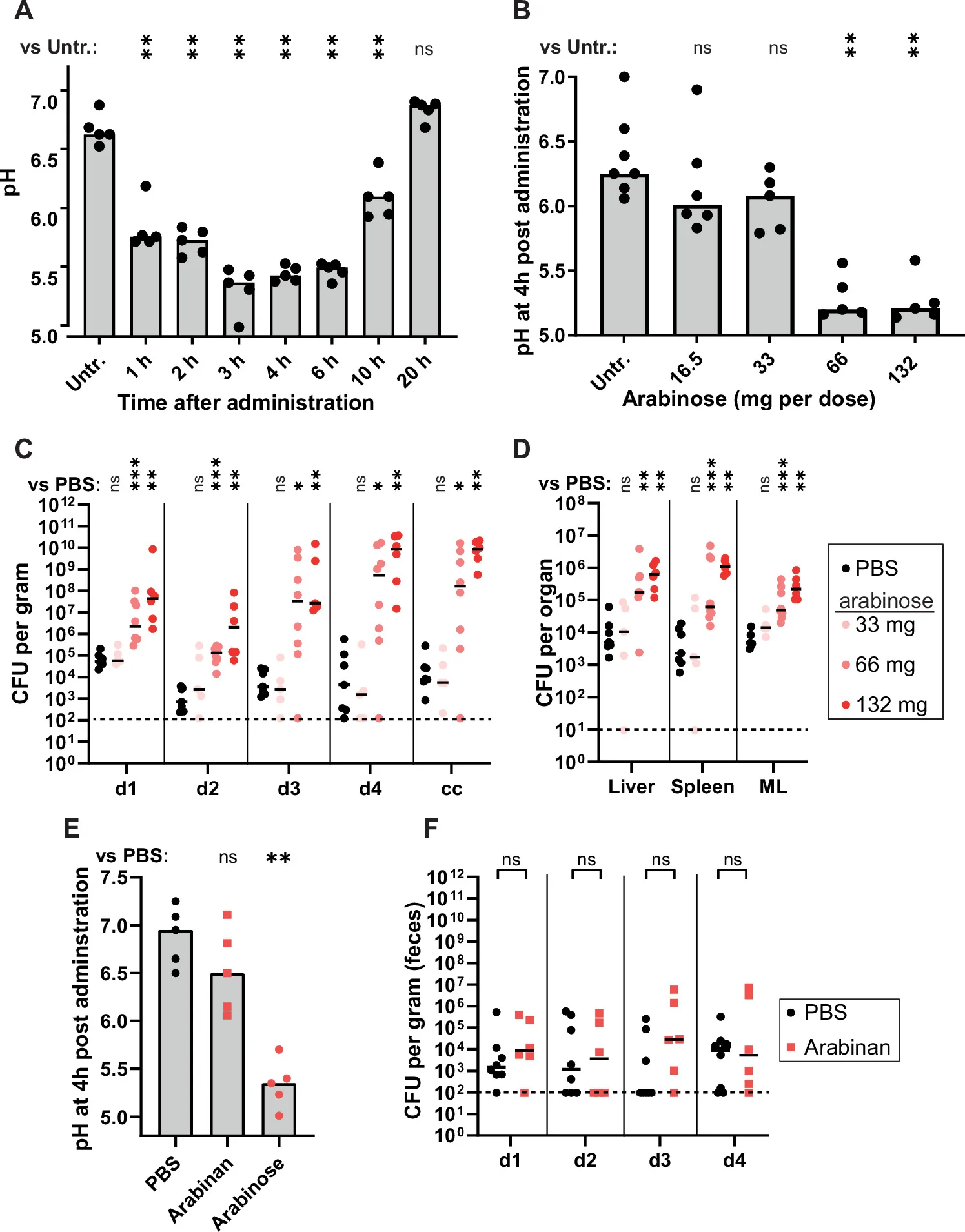
Gut acidification correlates with S.Tm blooms following arabinose ingestion. **A** Cecal pH was measured at the indicated time points following a single 132 mg dose of arabinose or untreated controls. *n* = 5 mice per time point. **B** Cecal pH measured 4 h after administration of two doses of arabinose at the indicated amounts. *n* = 7, 6, 5, and 5 mice for the untreated, 16.5-, 33-, 66-, and 132-mg groups, respectively. **C**,** D** Fecal and organ *S*.Tm loads in mice given two doses of varying amounts of arabinose (*n* = 5, 8, and 6 mice for the 33-, 66-, and 132-mg groups, respectively) or mock treatment (PBS; *n* = 7 mice). **E** Cecal pH measured 4 h after two doses of 132 mg arabinan, arabinose, or PBS. *n* = 5 mice per group. **F** Fecal *S*.Tm loads in infected mice administered two doses of 132 mg arabinan (*n* = 6 mice), or PBS (*n* = 8 mice). At least two independent experiments were performed. The two-tailed Mann-Whitney U test was used for statistical analysis; *P < 0.05; **P < 0.01; ***P < 0.001; ns, not significant. Horizontal lines indicate medians. The source data and *P* values for panels **A–F** are provided in the Source Data file.

While arabinose utilization promotes microbial growth, its rapid breakdown can also lead to growth-inhibiting acidification through the accumulation of fermentation byproducts. Acid stress suppresses the growth of several gut commensals in culture^44^. Indeed, a single arabinose dose rapidly lowered the cecum-luminal pH from ~6.8 to ~5.7 within 1 h, reaching 5.0–5.5 by 3 h post-administration before returning to baseline after ~10 h (Fig. 3A). A pH of 5.5 inhibits growth of Bacteroidota in culture while being better tolerated by some members of Bacillota^45,46^, consistent with the observed decrease in Bacteroidota and increase in Bacillota 8 h after arabinose administration (Fig. 2A). Because *Bacteroides* spp. contribute to colonization resistance against *Salmonella*^47^, their reduction under acidic conditions may explain the initial *S*.Tm bloom. In vitro, acidity impaired the growth of commensals such as *B. thetaiotaomicron* and *E. rectale* more than *S*.Tm (Supplementary Fig. 3B**)**, suggesting a potential mechanism for the selective advantage of *S*.Tm under acidic conditions. To assess the link between infection susceptibility and gut acidification, we administered graded doses of arabinose to identify the threshold required to promote *S*.Tm infection. Higher arabinose doses (≥66 mg) lowered the pH (≈5.5), increased fecal *S*.Tm, and elevated systemic bacterial loads, whereas lower doses (≤33 mg) did not (pH ≈ 6.8) with little effect on *S*.Tm colonization resistance (Fig. 3B–D). Mice receiving arabinan (2 × 132 mg), an arabinose polymer that is not rapidly fermented, showed less pronounced acidification and no associated *S*.Tm blooms (Fig. 3E, F**)**. Arabinan is a plant-derived branched polysaccharide containing arabinofuranose monomers that requires multiple enzymes for its breakdown, and generally has a lower turnover than free monosaccharides^48^. These data suggest an association between gut acidification and reduced colonization resistance.

### Arabinose overconsumption alters metabolite concentrations in the gut lumen

**Fig. 4:**
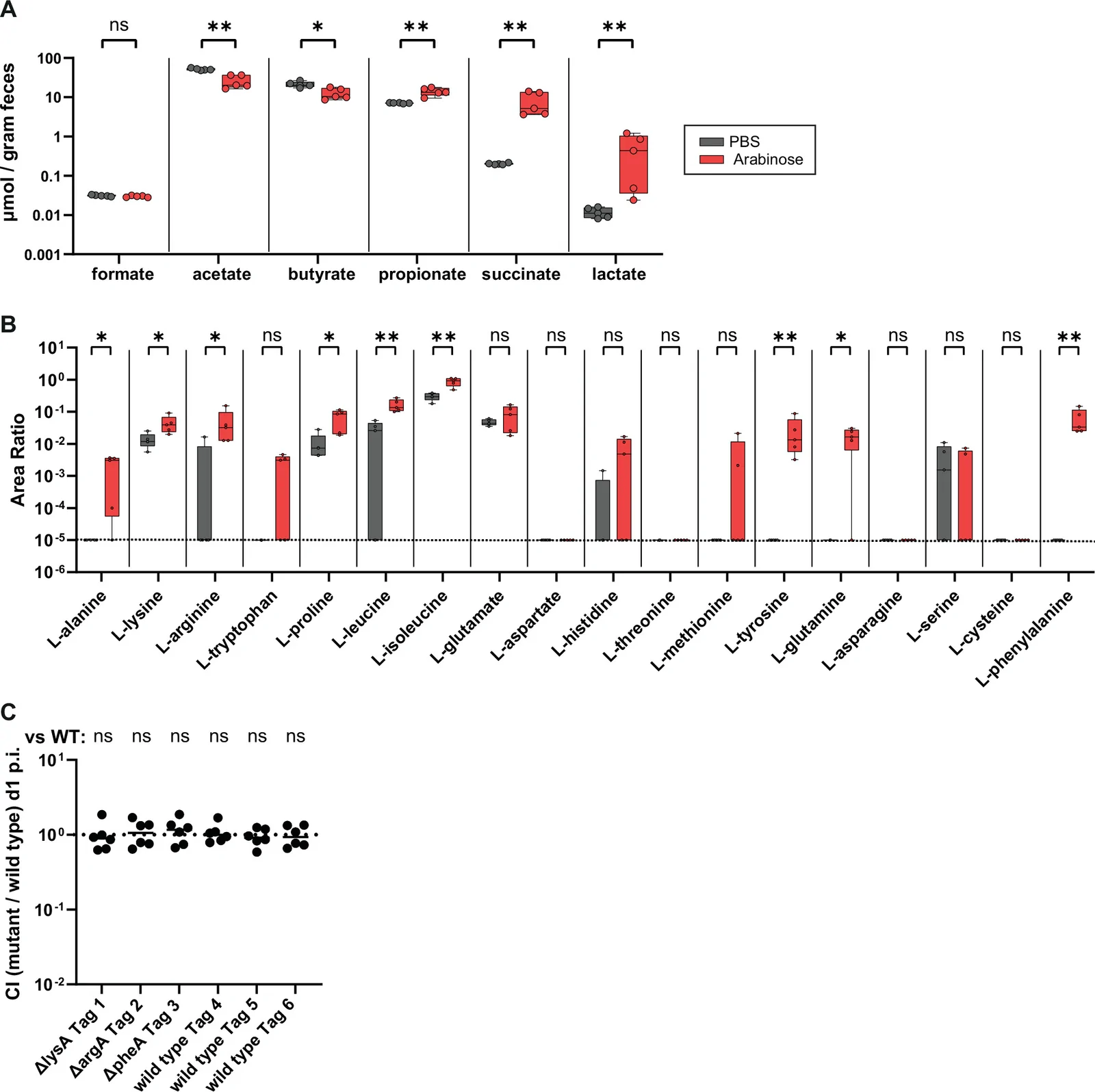
The microbiota metabolize arabinose, altering gut metabolite profiles. **A**,** B** Metabolite measurements of short-chain fatty acids (SCFAs), carboxylates, and amino acids in cecal contents from mice administered two doses of 132 mg arabinose or mock treatment (PBS). *n* = 5 mice per group. Samples were collected 4 h after the final dose. **A** LC-MS quantification of formate, acetate, butyrate, propionate, succinate, and lactate. **B** LC-MS quantification of relative amino acid levels. **C** Arabinose-overfed mice (two oral doses of 132 mg arabinose; *n* = 6 mice) were infected with a consortium of tagged lysine-, arginine-, and phenylalanine-auxotrophic mutants plus three isogenic wild-type strains at equal titers (5 × 10^7^ total CFU). Strain abundances were quantified by qPCR using tag-specific primers. Competitive indices (CI) were calculated relative to the mean abundance of the wild-type strains. The two-tailed Mann–Whitney *U* test was used for statistical analysis; *P < 0.05; **P < 0.01; ***P < 0.001 ns, not significant. *n* = 6 mice per group. Horizontal lines indicate medians. The source data and *P*-values for panels **A–C** are provided in the Source Data file.

Carbons from arabinose enter glycolysis via the pentose phosphate pathway and are incorporated into fermentation byproducts in the form of organic acids, such as SCFAs, succinate, and lactate^49–51^. Targeted liquid chromatography-mass spectrometry (LC-MS) detected significant increases in propionate, succinate, and lactate in the mouse gut four hours after the second dose of arabinose, whereas butyrate and acetate decreased, and formate remained unchanged (Fig. 4A). These shifts in organic acid levels are consistent with the observed reduction in luminal pH.

Arabinose ingestion also caused significant increases in several amino acids, namely alanine, lysine, arginine, proline, leucine, isoleucine, tyrosine, glutamine, and phenylalanine in the cecal contents of mice (Fig. 4B). These increases may reflect reduced microbial uptake or release of intracellular metabolites through microbial lysis. Elevated amino acid availability may contribute to *S*.Tm blooms as excess amino acids promote *S*.Tm colonization in complex or dysbiotic microbiota^52,53^. Indeed, the high competitive fitness of auxotrophic strains suggests that amino acid import was sufficient to support *S*.Tm growth in arabinose-overfed mice (C.I. ≈ 1.0; Fig. 4C). Overall, these findings support a model in which gut acidification disrupts the microbiota, subsequently reshaping the metabolic landscape to favor *S*.Tm expansion.

### Impact of virulence factors on gut luminal growth

**Fig. 5:**
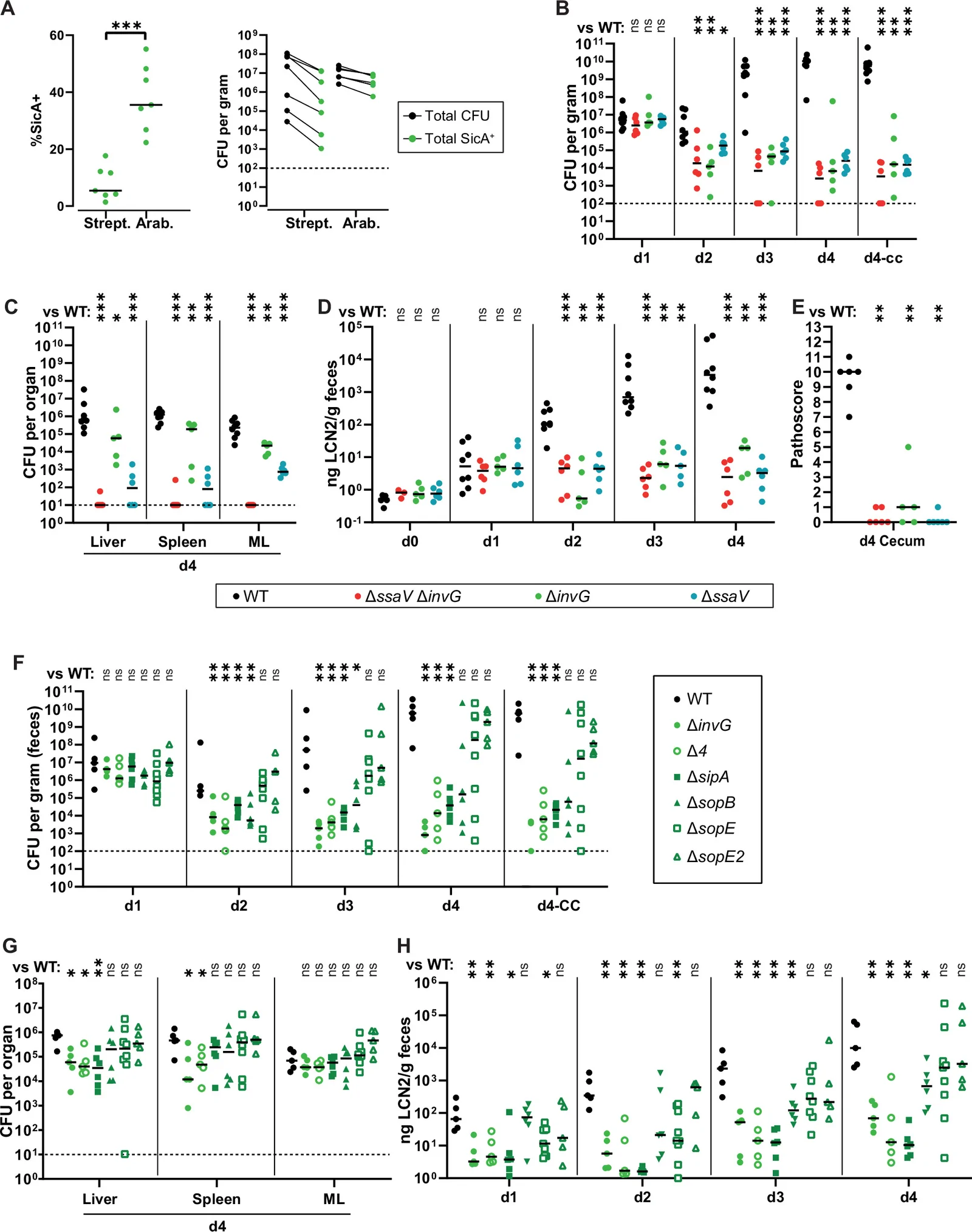
In arabinose-overfed mice, virulence is induced and required for luminal growth. **A** Percentage and total number of *S*.Tm expressing *sicA* at 8 h p.i. in feces from mice pre-treated with streptomycin or administered two doses of 132 mg arabinose. *n* = 7 mice per group. **B–E** Mice given arabinose were orally infected with 5 × 10^7^ CFU of one of the indicated strains: wild-type (WT; *n* = 8 mice), SPI-1 mutant (Δ*invG*; *n* = 5 mice), SPI-2 mutant (Δ*ssaV*; *n* = 6 mice), or SPI-1 SPI-2 double mutant (Δ*invG* Δ*ssaV*; *n* = 6 mice). **B** Pathogen loads in feces (d1-d4) and cecal contents (CC). **C** Bacterial loads at systemic sites (liver, spleen, and mesenteric lymph nodes (ML)). **D** Fecal lipocalin-2 (LCN2) levels. **E** Histopathological assessment of cecal tissue collected on day 4 p.i. **F–H** Arabinose-fed mice were orally infected with 5 × 10^7^ CFU of one of the indicated strains: wild type (WT; *n* = 5 mice), Δ*invG* (*n* = 5 mice), Δ4 (Δ*sipA*, Δ*sopB*, Δ*sopE*, Δ*sopE2; n* = 5 mice), Δ*sipA* (*n* = 6 mice), Δ*sopB* (*n* = 6 mice), Δ*sopE* (*n* = 8), or Δ*sopE2* (*n* = 5 mice). **F** *S*.Tm loads in feces and cecal contents. **G** Bacterial loads in systemic organs. **H** Fecal LCN2 levels. The two-tailed Mann-Whitney U test was used for statistical analysis; *P < 0.05; **P < 0.01; ***P < 0.001; ns, not significant. Horizontal lines indicate medians. The source data and *p*-values for panels **A–H** are provided in the Source Data file.

Colonization kinetics and marked systemic pathogen loads point to enhanced pathogen invasiveness in LDS overconsuming mice (Fig. 1B, C). Increased invasion into intestinal tissue and subsequent reseeding may contribute to the luminal *S*.Tm blooms at days 3-4 p.i., while enhanced dissemination likely explains the elevated pathogen burdens in the mesenteric lymph nodes, spleen and liver, which exceeded those in streptomycin-pretreated controls. Virulence genes carried on two pathogenicity islands (SPI-1 and SPI-2) enable *S*.Tm to invade and replicate within intestinal and immune cells, such as dendritic cells and macrophages, facilitating systemic spread^54–58^. We hypothesized that LDSs induce SPI-1 expression and thereby promote tissue invasion. Using a transcriptional GFP reporter for the SPI-1 chaperone SicA^59^, we observed a fivefold higher percentage of *sicA* + (GFP+) *S*.Tm in arabinose-overfed mice compared to streptomycin-pretreated mice (Fig. 5A), indicating increased SPI-1 expression and invasive potential.

While SPI-1 and SPI-2 and their effectors are essential for inflammation and disease progression, they are not inherently required for gut luminal growth in antibiotic-pretreated mice, where the microbiota is strongly perturbed. Accordingly, SPI-1 and SPI-2-deficient mutants colonize the guts of streptomycin-pretreated mice as efficiently as wild type^56,60^. By contrast, both SPI-1 and SPI-2 virulence were required for colonization and pathogen growth in mice receiving arabinose. Mutants lacking essential components of either the SPI-1 or SPI-2 type III secretion systems (Δ*invG* and Δ*ssaV*, respectively) or both (Δ*invG* Δ*ssaV*) colonized the gut similarly to wild-type at day 1 p.i. but became severely attenuated thereafter (days 2–4 p.i.), with fecal loads 5–6 logs lower than wild-type (Fig. 5B). Infection with these mutants also yielded reduced organ counts, lower lipocalin levels, and milder mucosal pathology, consistent with a marked defect in virulence (Fig. 5C–E). These observations suggest that the requirement for SPI-1 and SPI-2 in *S*.Tm colonization is context dependent, and that arabinose overconsumption provides a robust model for studying the role of SPI-1 and -2 virulence in infection, transmission or virulence evolution.

Given the strong contribution of SPI-1 to *S*.Tm luminal blooms at days 3–4 p.i., we next dissected the role of individual SPI-1 effectors in this process. SPI-1 effectors, namely SipA, SopB, SopE, and SopE2, manipulate the host cytoskeleton to enable *S*.Tm entry into non-phagocytic cells. Among these, SipA plays the most significant role in the invasion of enteric epithelial cells in vivo and polarized cells in tissue culture^61^. However, contributions of SPI-1 effectors to gut-luminal colonization were not resolved. In arabinose-overconsuming mice, the Δ*sipA* mutant behaved similarly to mutants lacking all four effectors or a SPI-1 secretion-deficient mutant, exhibiting an initial bloom on day 1 p.i., followed by a marked attenuation in fecal counts (Fig. 5F). Δ*sipA* infection also resulted in reduced dissemination to the liver (day 4 p.i.; Fig. 5G) and lower levels of fecal lipocalin throughout the infection (days 1–4 p.i.; Fig. 5H). The Δ*sopB* mutant was also attenuated, albeit less severely than Δ*sipA*, and showed moderate fecal shedding and reduced inflammation (Fig. 5F, H). By contrast, SopE played only a minor role, as Δ*sopE*-infected mice resembled wild-type infection, except for a slight delay in inflammation (i.e., at day 2 p.i.; Fig. 5F, H). SopE2 appeared redundant, as the Δ*sopE2* mutant was phenotypically indistinguishable from wild type.

These observations highlight a functional hierarchy among SPI-1 effectors in vivo that mirrors prior invasion studies, with SipA being most critical for mucosal infection, followed by SopB, whereas SopE/SopE2 appear largely dispensable^61,62^. Importantly, the effects of arabinose overconsumption tie invasion to gut-luminal blooms, a link not captured in earlier in vivo models^60^.

**Fig. 6:**
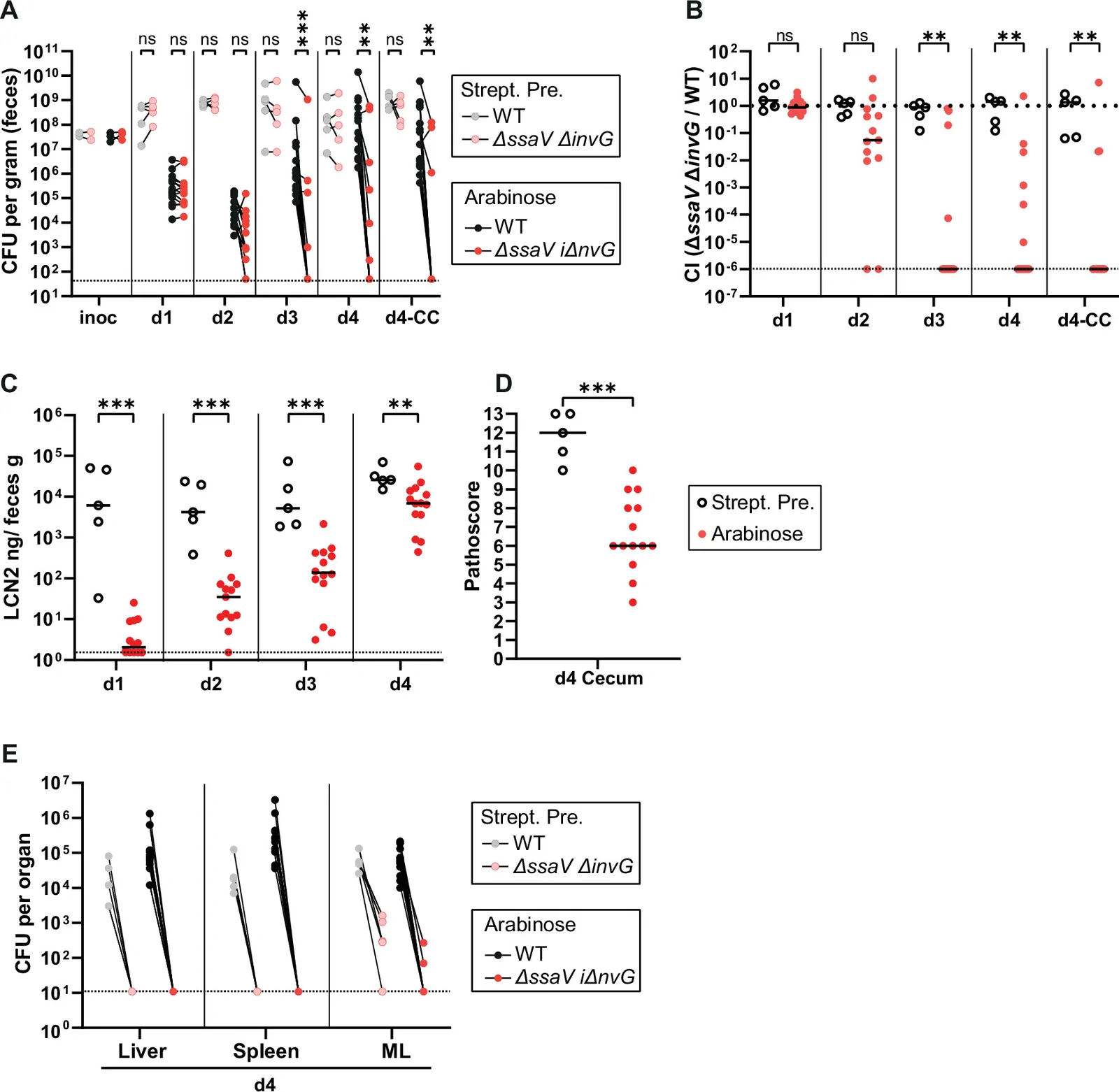
Arabinose overconsumption selects against avirulent *S*.Tm mutants. Mice pre-treated with streptomycin (*n* = 5 mice) or administered two doses of 132 mg arabinose (*n* = 14 mice) were orally infected with a 1:1 mixture of wild-type (WT) *S*.Tm and the avirulent mutant strain (Δ*invG* Δ*ssaV*). **A** Pathogen loads in feces (d1-d4) and cecal contents (CC). **B** Competitive indices (CI) comparing the relative fitness of the wild-type and the avirulent strain. **C** Fecal lipocalin-2 (LCN2) levels. **D** Histopathological assessment of cecum tissue collected on day 4 p.i. The two-tailed Wilcoxon and Mann–Whitney U tests were used for statistical analysis; *P < 0.05; **P < 0.01; ***P < 0.001; ns, not significant. **E** Pathogen loads at day 4 p.i. in the liver, spleen, and mesenteric lymph nodes (ML). Abbreviations: inoc inoculum. Horizontal lines indicate medians. The source data and *p*-values for panels **A–E** are provided in the Source Data file.

The inability of virulence-attenuated mutants to maintain gut colonization in arabinose-overconsuming mice might stem from their failure to induce inflammation. Inflammation promotes Enterobacteriaceae blooms by generating niche-specific nutrients, including tetrathionate and nitrate, that *S*.Tm can exploit for anaerobic respiration and oxidative TCA cycle metabolism^63–67^. To test this, we performed a 1:1 co-infection with a SPI-1, SPI-2-secretion-deficient (Δ*invG* Δ*ssaV*) mutant and a congenic wild-type strain to trigger inflammation in trans. On day 1 p.i., both strains bloomed to similar levels as indicated by fecal shedding (Fig. 6A). Consistent with previous observations (Fig. 1B), the total fecal pathogen loads plummeted by day 2 and rebounded to 10^6^–10^10^ CFU per gram feces by days 3–4 p.i. (Fig. 6A). Strikingly, by day 4 p.i., the wild-type strain had completely outcompeted the Δ*invG* Δ*ssaV* mutant, whose fecal counts fell below detection in 6 of 12 mice (Fig. 6A, B). The onset of inflammation, indicated by elevated lipocalin levels and histopathology, coincided with a rise of the wild-type strain and a sharp decline of the Δ*invG* Δ*ssaV* mutant population (Fig. 6C, D). These findings suggest that SPI-1 and SPI-2 virulence not only trigger mucosal inflammation but also confer a strong competitive advantage during the days 3–4 p.i. luminal bloom. The reduced fitness of the Δ*invG* Δ*ssaV* mutant was not due to an intrinsic growth defect, as it grew comparably to wild type in streptomycin-pretreated mice (Fig. 6A, B). The absence of the mutant at systemic sites confirms that it remained confined to the gut lumen (Fig. 6E). Thus, the dominance of wild-type *S*.Tm in the lumen at days 3–4 p.i. further suggests that the luminal population is substantially replenished by re-seeding wild-type *S*.Tm from tissue reservoirs established via SPI-1 and/or SPI-2 virulence. Previous work in antibiotic-pretreated mice suggests such re-seeding events are comparatively rare^68^. We substantiated this hypothesis using pools of barcoded wild-type *S*.Tm strains in arabinose-overfed mice (Supplementary Fig. 4). Overall, the data suggest that virulence and gut colonization are tied in the diet-driven infection model, but uncoupled in the antibiotic-pretreatment model, underscoring how infection context shapes the selective pressures governing pathogen fitness.

### Arabinose overconsumption influences tissue tropism

**Fig. 7:**
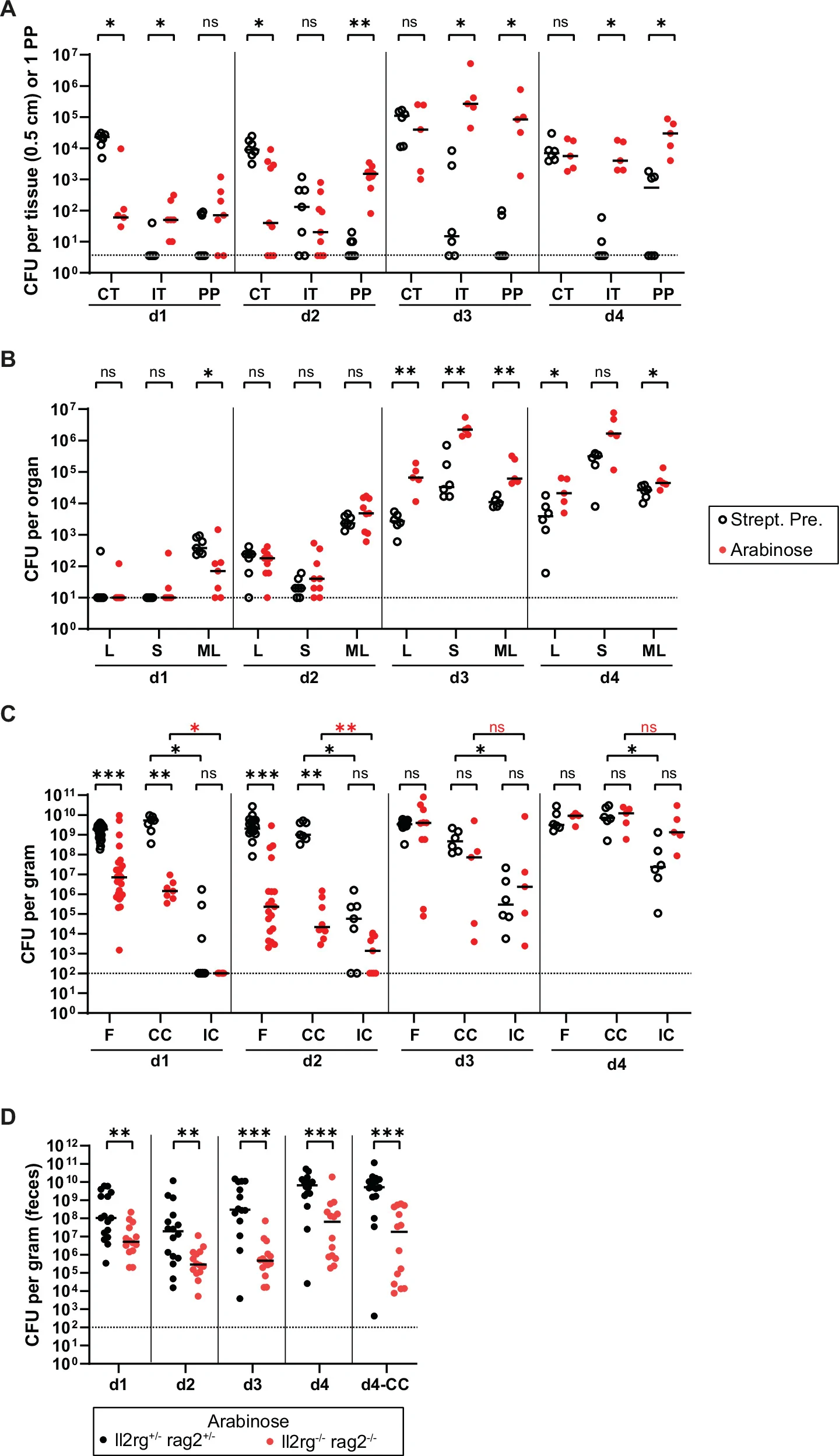
*S*.Tm tissue tropism upon arabinose overconsumption. **A–C** Mice were pretreated with streptomycin (*n* = 7, 7, 6, and 6 mice on days 1–4 p.i., respectively) or given two doses of 132 mg arabinose (*n* = 7, 9, 5, and 5 mice, respectively) and given 5 × 10^7^ *S*.Tm by oral gavage. **A** *S*.Tm extracted from indicated tissue (CT cecum tissue; IT ileum tissue; PP Peyer’s Patches) by gentamicin protection assay and enumerated by plating on MacConkey agar. **B** Pathogen loads in systemic organs: liver (L), spleen (S), and mesenteric lymph nodes (ML). **C** Pathogen loads in intestinal contents: feces (F), cecal contents (CC), and ileum contents (IC). **D** Heterozygous (*n* = 16 mice) and homozygous (*n* = 14 mice) *Il2rg*/*Rag2* mice were given two doses of 132 mg arabinose and orally infected with 5 × 10^7^ CFU of *S*.Tm. *S*.Tm counts in fecal (d1–d4) and cecal contents (CC). The two-tailed Mann–Whitney U and Wilcoxon tests were used for statistical analysis; *P < 0.05; **P < 0.01; ***P < 0.001; ns, not significant. Horizontal lines indicate medians. The source data and *P* values for panels **A–D** are provided in the Source Data file.

To identify intestinal reservoirs, we performed gentamicin protection assays on tissues from infected mice receiving either streptomycin or arabinose. This assay eliminates extracellular bacteria, allowing quantification of tissue-lodged bacteria. Sampled tissues included typical invasion and persistence sites, such as the cecum, ileum, and Peyer’s patches. In the arabinose group, *S*.Tm was distributed similarly across these tissues whereas in the streptomycin-pretreated mice, *S*.Tm localized preferentially in cecum tissue **(**Fig. 7A). Tissue loads in arabinose-overfed mice rise until day 3 of infection and plateaued (or slightly decline) by day 4 p.i. unlike the streptomycin-pretreatment group, where tissue burdens changed little over time (Fig. 7A). Systemic pathogen loads (liver, spleen, and mesenteric lymph nodes) increased in both groups, but by day 3 p.i. were higher in the arabinose-overfed mice than in the streptomycin-pretreatment controls (Fig. 7B).

Early in infection (days 1–2 p.i.), the luminal *S*.Tm population was concentrated in the cecum and colon with lower titers in the ileum in both treatments (as inferred from cecal and ileal contents and feces; Fig. 7C). In arabinose overconsuming mice, this pattern shifted by day 3 p.i., when ileal *S*.Tm titers became comparable to cecal titers. In contrast, streptomycin-pretreated mice exhibited only limited *S*.Tm expansion in the lumen of the ileum, with titers remaining significantly lower than in cecal contents (Fig. 7C). These data may suggest that reseeding from ileal tissues contributes to *S*.Tm shedding in arabinose overconsuming mice, as substantial bacterial titers were detected in both ileal tissue and Peyer’s patches following blooms in ileal contents. To investigate the contribution of Peyer’s patches to reseeding, we tested *Il2rg*^*−/−*^* Rag2*^*−/−*^ mice, which lack Peyer’s patches. These mice exhibited lower fecal *S*.Tm loads compared to +/− littermate controls (Fig. 7D). In contrast, *S*.Tm systemic burdens were much higher in the knockout mice, likely due to the absence of NK cells, which release pro-inflammatory cytokines that accelerate gut inflammation while restricting systemic spread^69–72^ (Supplementary Fig. 5A). The reduction in fecal shedding may suggest that Peyer’s patches contribute to luminal reseeding. However, we cannot directly attribute the decreased fecal loads to the absence of Peyer’s patches, as knockout mice also exhibited reduced inflammation compared to heterozygous controls, which could independently limit luminal *S*.Tm growth (Supplementary Fig. 5B).

## Discussion

Foodborne pathogens are frequently encountered through contaminated food, yet most exposures do not result in disease due to microbiota-mediated CR. Antibiotics are among the best-established risk factors for enteric infection, but many cases occur without recent antibiotic exposure, suggesting additional susceptibility determinants. In humans, dietary contributions remain poorly defined, as studies largely focus on identifying contaminated food sources. Foodborne infections caused by *Salmonella enterica* and *Escherichia coli* exhibit seasonal spikes, including during holiday periods, typically attributed to temperature, food handling, and storage practices^73–79^. Such periods can also involve short-term dietary alterations and excess food intake, though whether these contribute to infection susceptibility remains unknown. However, animal studies show that diet-elicited perturbations can affect the gut microbiota and weaken CR^2,80^. High-fat Western diets promote excessive bile salt secretion, which most Bacteroidetes and Firmicutes strains cannot tolerate, permitting blooms of tolerant Enterobacteriaceae such as *S*.Tm and *E. coli*^81^. Here, we broaden current understanding of diet-mediated perturbations by showing how acute overconsumption of specific carbohydrates can not only disrupt CR, but also reshape pathogen–microbiota interactions in ways that enhance pathogen virulence.

Arabinose intake induced reversible changes in gut metabolites, pH, and microbiota composition. Metabolite and pH analyses revealed that arabinose was rapidly fermented, causing organic acid accumulation and subsequent gut acidification (Fig. 3). This pH shift may have reversibly altered the microbiota composition (Fig. 2A) and promoted pathogen growth through increased availability of nutrients, including amino acids that supply carbon and nitrogen (Fig. 4B). *S*.Tm possesses multiple acid resistance systems^82–84^, enabling it to withstand gastric acidity (pH 2–3)^85^ and proliferate within macrophages’ acidic compartments (pH 4.8)^86–90^. This resistance may give *S*.Tm a competitive edge over the microbiota under mild pH stress (Supplementary Fig. 3B).

Recent studies identified arabinose as a key carbon source for pathogen growth. *S*.Tm acquires arabinose via a genome-encoded alpha-N-arabinofuranosidase that liberates the sugar from dietary polysaccharides to support its expansion in superspreader mice^91^. Additionally, continuous supplementation of free L-arabinose in drinking water throughout the infection increased pathogen burdens^91,92^. In these settings, arabinose catabolism contributed substantially to pathogen fitness, as mutants defective in arabinose utilization were attenuated. Surprisingly, in our model, defective arabinose catabolism did not prevent *S*.Tm colonization or disease when arabinose was administered as two high doses around the time of infection (Fig. 1L). With this regimen, the ∆*araB* mutant featured merely a slight disadvantage (Fig. 1M). This suggests that *S*.Tm expansion in our model primarily depends on a dysbiotic microbiota rather than direct utilization of a supplemented sugar. Differences in experimental design, particularly acute overconsumption versus continuous intake, likely explain the varying reliance on arabinose catabolism.

The mechanisms by which other LDSs tested, namely lactulose, erythritol, maltitol, and sorbitol, compromise colonization resistance remain unexplored. Sorbitol and erythritol did not alter intestinal pH (Supplementary Fig. 6), suggesting that acidification does not underlie their effects. However, lactulose and maltitol appear to act through a similar mechanism, as both induce a comparable drop in pH, albeit less pronounced with maltitol (Supplementary Fig. 6). This raises the possibility that gut acidification may represent one broader mechanism by which certain rapidly fermentable foods weaken colonization resistance. Further studies should address whether different LDSs elicit distinct microbiota perturbations and assess how different perturbations reduce CR.

Overconsumption alone does not inherently compromise colonization resistance, as arabinan, glucose, fructose, galactose, and sucrose did not support *S*.Tm colonization (Figs. 1F and 3F). This may reflect efficient host absorption of preferred sugars, limiting their availability to the microbiota^33^, or the slower breakdown of dietary fibers, resulting in only modest pH changes insufficient to substantially alter the microbial community^48^. Thus, the risk associated with overconsumption depends on what is overconsumed.

We demonstrated that dietary components could influence pathogen virulence in several ways, including increased virulence expression, broader tissue invasiveness, and selection against avirulent mutants. Despite milder disease sequelae and slow infection kinetics, mice overfed on arabinose or other LDSs unexpectedly exhibited high pathogen loads at systemic sites **(**Fig. 1B, C**)**. The increased fraction of SPI-1^+^ *S*.Tm cells likely contributes to increased dissemination (Figs. 1C and 5A, 7B). This enrichment of SPI-1^+^ cells cannot be directly attributed to L-arabinose, as previous studies have shown that L-arabinose represses SPI-1 by inhibiting the SPI-1 master regulator HilD and reducing cell invasion in vitro^93^. Instead, altered gut metabolites likely contributed to SPI-1 induction. Microbiota-derived metabolites, such as SCFAs, are known to modulate virulence gene expression. Formate and acetate upregulate HilD expression via BarA/SirA signaling^94^, whereas propionate and butyrate exert inhibitory effects^47,95,96^. Metabolite analysis revealed increased propionate and decreased butyrate and acetate levels (Fig. 4A). Although these changes would generate a mix of opposing regulatory signals for SPI-1, we observed an overall increase in SPI-1⁺ cells, suggesting that SPI-1 induction may result from the collective shift or from additional unidentified microbiota-derived signals. Future work should address how these metabolites interact to regulate SPI-1 expression, as this interplay may have important implications for pathogen virulence in vivo.

Increased lactate and succinate were also observed (Fig. 4A). These metabolites, along with low pH, are known inducers of SPI-2 expression intracellularly and in culture^97–100^ suggesting that SPI-2 may likewise be induced in vivo. While we did not assess SPI-2 expression in the gut lumen, where its role remains less defined, prior studies show that SPI-2 transcription is primed before invasion^101,102^, potentially enhancing *S*.Tm survival within host tissues^103^. Follow-up studies should determine whether luminal SPI-2 expression contributes to early-stage virulence.

Arabinose intake broadens tissue tropism, likely contributing to higher systemic burdens. It increased pathogen loads in ileal tissue, particularly Peyer’s patches, whereas significantly lower loads were observed in antibiotic-pretreated mice (Fig. 7A). Peyer’s patches function in immune surveillance but also serve as entry points for several bacterial pathogens, including other *S. enterica* spp.^104–106^, *Yersinia enterocolitica*^107,108^, and *Vibrio cholerae*^109^, which exploit the microfold cells to access the lamina propria for dissemination. These specialized lymphoid tissues are enriched in macrophages and dendritic cells, capable of trafficking *Salmonella* to mesenteric lymph nodes, the bloodstream, and distal organs such as the spleen and liver^110,111^. In contrast, the cecum becomes the primary site of infection in antibiotic-pretreated mice, explaining the comparatively lower pathogen burdens at systemic sites. In arabinose overfed mice, elevated virulence expression may promote access to Peyer’s patches or other immune inductive sites where SPI-2 virulence factors are especially crucial for evading innate immune responses. The exact mechanisms underlying this altered tissue tropism remain unclear and warrant further investigation.

Experimental evolution studies reveal that the microbiota selects against virulence-attenuating mutations^112–116^. During *S*.Tm infection of antibiotic-pretreated mice, the combined effects of antibiotics and inflammation on the microbiota mediate a trade-off between pathogen replication and virulence loss^115^. *S*.Tm achieves optimal growth by depleting the microbiota through SPI-1 and SPI-2-driven inflammation^67^, but this also promotes the luminal overgrowth of virulence-attenuating *hilD* mutations^117^. While the mechanisms underlying the selection for virulence-attenuated mutants remain poorly understood, the arabinose infection model provides a setting in which virulence is positively selected in the gut-luminal pathogen population. *S*.Tm required SPI-1 and SPI-2 for sustained gut colonization in arabinose overfed mice (Fig. 5B), whereas these virulence factors were dispensable under antibiotic pretreatment^56^ (Fig. 6A, B). While inflammation promoted *S*.Tm growth in both models, only in the arabinose infection model was the growth of avirulent mutants significantly constrained (Figs. 5B and 6A, B). This can be explained by the microbiota’s control over pathogen population dynamics in the gut lumen between days 1–3. Notably, gut acidification triggers transient dysbiosis that allows an initial *S*.Tm bloom but also permits fast microbiota recovery that suppresses further growth and renders the luminal pathogen population susceptible to clearance by subsequent inflammatory responses (Fig. 2). As a consequence, avirulent mutants, confined to the gut lumen, are selected against, while reseeding tissue-resident *Salmonella* (carrying functional SPI-1 and SPI-2 virulence factors) establish a luminal population that retains its full virulence (Fig. 6). In contrast, antibiotic pretreatment extensively depletes the microbiota, allowing the luminal *S*.Tm population to expand to high densities that resist clearance even when SPI-1 and SPI-2 are defective (Fig. 6A, B). Incomplete clearance of luminal *S*.Tm permits the overgrowth of avirulent mutants, particularly *hilD* mutants, which can arise through spontaneous mutation^117^ and replicate faster than wild-type^59^. Our study suggests that diet can mediate pathogen:microbiota interactions that favor virulence.

The LDSs under study are commonly used as medications or food additives. Arabinose has antidiabetic properties, including sucrase inhibition^118^, while lactulose is widely used as both a laxative and a treatment for hyperammonemia^119,120^. Maltitol, erythritol, and sorbitol are common sugar substitutes found in low-calorie diet foods^121^. Although our findings may raise food safety concerns, infection susceptibility was dose-dependent and occurred only at large, acute doses of ~ 5 g/kg body weight. Moderate intake did not produce the same effect. A human clinical study found that a 50 g dose of lactulose did not alter gut microbiota composition or trigger *E*. coli blooms in healthy adults^122^, suggesting that tolerable doses are unlikely to compromise CR. In our study, lower arabinose doses failed to weaken CR and failed to induce pronounced gut acidification. Thus, moderate fermentation was insufficient to overwhelm host buffering capacity. Organic acids are neutralized by host-derived bicarbonate, secreted via the SLC26 family of Cl⁻/HCO_3_^−^ and SCFA/HCO_3_⁻ exchangers, thereby maintaining pH along the intestinal tract^123,124^. Disruption in these transporters causes colitis and microbiota dysbiosis in mice^125^ and is associated with increased inflammatory bowel disease incidence in humans^126–128^. These studies underscore that pH homeostasis supports a resilient gut microbiota.

While evidence supports the safety of LDSs at moderate doses, the literature indicates that outcomes can vary depending on dietary context and duration of intake. For example, fructo-oligosaccharides and xylo-oligosaccharides, touted as prebiotics for promoting *Lactobacillus* and *Bifidobacterium* growth, suppress the growth of *E. coli*, *C. jejuni*, and *S*. Enteritidis in vitro^129^. However, prolonged supplementation in mice with 10% fructo-oligosaccharides in mice chow or water increased systemic *Salmonella* loads and fecal shedding when consumed two weeks prior to pathogen challenge^130^. Moreover, fructo-oligosaccharides consumption amplified *Salmonella* blooms in rats fed on a low calcium, high-fat Western diet^131^. Histopathology was not assessed in these studies, making it unclear whether there was overt salmonellosis, as hallmark symptoms like colitis and diarrhea were not directly evaluated. Together, these findings emphasize that although moderate intake of LDSs may generally be safe, specific contexts can disrupt microbial homeostasis and promote pathogen expansion.

Several limitations should be considered. Our findings are based on animal models, and the contribution of CR to human enteric infection susceptibility remains inferred rather than directly established. We also examined only a limited set of individual carbohydrates, whereas human diets involve complex mixtures that may exert synergistic or buffering effects on the microbiota. Whether similar mechanisms operate in more physiologically relevant dietary contexts remains to be determined. Nevertheless, our study highlights the importance of evaluating not only the nutritional but also the microbiological consequences of emerging dietary interventions. Given the impact of dietary compounds on pathogen virulence, we recommend that prebiotic tolerability testing include assessments of infection susceptibility.

## Methods

### Experimental model and subject details

#### Mouse lines

All mice used in this study originated from C57BL/6 J breeders, initially obtained from Jackson Laboratories and housed at the EPIC mouse facility of ETH Zurich, Switzerland. Mice were maintained on a standard chow diet (Kliba Nafag, 3537; autoclaved), consisting of 4.5% fat, 18.5% protein, approximately 50% carbohydrates, and 4.5% fiber by weight. Mice were bred and maintained under specific pathogen-free, full-barrier conditions in individually ventilated cages at the ETH Zurich EPIC mouse facility, with a 12-h light/12-h dark cycle, a room temperature of 21 ± 1 °C, and a relative humidity of 50 ± 10%. For our experiments, we used 8- to 12-week-old mice, randomly assigning animals of either sex to experimental groups. All studies were conducted in accordance with ethical and legal requirements and were reviewed and approved by the Kantonales Veterinäramt Zürich under the licenses: ZH193/2016, ZH158/19, and ZH109/22.

### Bacteria strains

All *Salmonella* enterica serovar Typhimurium (*S*.Tm) strains used in this study are isogenic to *S*.Tm SB300, a re-isolate of SL1344^132^ from a systemic mouse infection in the Galán lab^133^. This strain was genome re-sequenced and is naturally resistant to streptomycin.

### Method details

#### Bacteria strains and culture conditions

Strain construction. Novel single-gene deletion strains were constructed using the λ Red recombination system^134^. PCR products amplified from pKD3 or pKD4 plasmid template using primers with 40-nt flanking sequences of the target gene were electroporated into wild-type *S*. Typhimurium harboring the pKD46 plasmid. Recombinant strains were then selected on LB agar supplemented with appropriate antibiotics. P22 phage lysates were prepared from the original mutant strains and used to transduce the mutation into the ancestral strain. A complete list of strains is provided in Supplementary Data.

*S*.T m strains were routinely grown overnight at 37 °C in Lysogeny broth (LB,^135^) with agitation under ambient conditions. Strains were stored at −80 °C in 1_3_% peptone 5% glycerol broth.

### Mouse infection experiments

#### Preparation of the inoculum

Bacteria were grown overnight for 12 h in high salt (0.3 M NaCl) LB medium supplemented with the appropriate antibiotics, then sub-cultured for 4 h in antibiotic-free (0.3 M NaCl) LB, washed twice in PBS, and resuspended in PBS. Mice were inoculated via oral gavage with 5 × 10^7^ bacterial and housed in individually ventilated cages.

#### Carbohydrate administration

Carbohydrates (listed in Supplementary Data) were dissolved in water, gently heated until fully dissolved, cooled to room temperature, and administered to mice via oral gavage.

#### Sample collection

Feces and intestinal contents were weighed and suspended in PBS. Liver, spleen segments, and mesenteric lymph nodes were homogenized in PBS/Tergitol (0.5% v/v)/BSA (0.5% w/v) using a TissueLyser (Qiagen) and plated on MacConkey agar supplemented with the appropriate antibiotics.

#### Gentamicin protection assay

Intestinal tissue segments were cut longitudinally, washed three times in PBS to remove luminal contents, and incubated in 400 μg/mL gentamicin (in PBS) for 45 min to kill extracellular bacteria. Samples were then washed six times in PBS to remove residual gentamicin and subsequently homogenized in PBS/Tergitol (0.5% v/v)/BSA (0.5% w/v) using a TissueLyser to release intracellular bacteria. Tissue suspensions were plated on MacConkey agar supplemented with the appropriate antibiotics to quantify viable *S*.Tm.

#### Inflammation assessments

Intestinal tissue samples were cryo-embedded for histopathological scoring as described in ref.^32^. 5 μm cryosections were prepared and stained with hematoxylin and eosin (H&E)^136^ to access enteropathy severity. Fecal Lipocalin-2 levels were measured using ELISA (DuoSet Lipocalin ELIZA kit, DY1857, R&D Systems, Minneapolis, MN, USA).

#### Intestinal barrier function

Using chambers were used to evaluate intestinal permeability as previously described^137^. Paracellular and transcellular permeability were assessed using 6 μCi/ml of 51Cr-EDTA and 5 × 10^5^ M HRP (type II; Sigma Aldrich) probes, respectively. Serosal 51Cr-EDTA was quantified using a liquid scintillation counter and reported as percent recovery/cm^2^/h.

### Metabolite measurements

#### Sample preparation

Cecum contents were collected, suspended in PBS and homogenized (without beads) using a Tissuelyzer (Qiagen) at 25 Hz for 2.0 min, followed by centrifugation for 5 min at 20,000 x *g* to pellet cells and particulate matter. Cell-free supernatants were collected and then centrifuged for 40 min at 20,000 x *g*, 4 °C to remove any remaining suspended particulate matter. Clear supernatants were transferred to clean microfuge tubes and stored at −80 °C until thawed for LC-MS processing. For acetate, butyrate, formate, lactate, and succinate, samples were derivatized using methods from^138^. In brief, samples were mixed with isotopic internal standards and reacted with the derivatizing reagents, 3NPH (3-nitrophenylhydrazone) and N3D (3-dimethylaminopropyl-N’-ethylcarbodiimide (EDC)·HCl) for 45 min at 40 °C. The reaction was quenched with trifluoroacetic acid (TFA) at a final concentration of 0.08% and then diluted in 70% isopropanol to a final concentration of 250 µg cecal content per ml. Derivatized samples were stored at −80 °C until measurement. For amino acids, the samples were mixed with an isotopic standard (^13^C5-labeled glutamate) and diluted in HILIC buffer to achieve a final concentration of 250 µg cecal content per ml. Samples were then stored at −80 °C until measurement.

#### Liquid chromatography–mass spectrometry

For each compound, calibration curves were made to determine its quantifiable concentration range. LC-MS analyses were carried out with an Ultimate 3000 ULPC instrument (Thermo Scientific, Waltham, MA, USA) hyphenated to a QExative plus mass spectrometer (Thermo Scientific, Waltham, MA, USA). In case of short-chain fatty acid and carboxylate analysis, we applied a previously described method with slight modifications^138^. For LC separation, we used a Kinetex XB C18 column (particle size 1.7 µm; 50 × 2.1 mm, Phenomenex, Torrance, CA, USA) with 0.1% formic acid in water (solvent A) and 0.1% formic acid in acetonitrile as mobile phases. The sample injection volume was 2 µl. At a flow rate of 500 µl min^−1^, we run a linear gradient with solvent B changing as follows: 0 min: 2%; 3 min: 95%; 5 min: 95%; 5.3 min: 2%. Subsequently, the column was equilibrated for 2 min at the initial condition. The applied amino acid analysis method was based on a previously described Method^139^ adapted to the MS instrument. For LC separation, we used an AQUITY BEH NH2 column (particle size 1.7 µm; 100 × 2.1 mm, Waters, Milford, MA, USA) with a buffer consisting of 10 mM ammonium formate (short buffer) in water: acetonitrile (50:50) (solvent A) and the same buffer in acetonitrile: water: methanol (95:5:5) as mobile phases. To shift pH, 1.76 ml of formic acid was added to both mobile phases. The sample injection volume was 2 µl. At a flow rate of 500 µl min^−1^ we run a linear gradient with solvent B changing as follows: 0 min: 84.3%; 1.5 min: 84.3%; 5.5 min: 5.3%; 7.5 min: 5.3%; 8 min: 84.3%, 10 min: 84.3%. Heated electro spray ionization was performed in both cases by FTMS at a mass resolution of 70’000 (at m/z 200). SCFAs and carboxylates were measured in the negative mode applying a spray voltage of −2.7 kV and amino acids in the positive mode with a spray voltage of +3.4 kV. All other source parameters were the same and set as follows: spray voltage: 2.7 kV; S-lens RF level: 50; sheath gas: 50; aux gas: 20; sweep gas: 0; aux gas heater: 350 °C.

### Bacterial flow cytometry

SicA promoter activity was assessed in mice using a plasmid-based *PsicA*-gfp reporter^59^. Fresh feces collected 8 h.p.i. were diluted in PBS and incubated for 1 h at room temperature with 2 μg ml^−1^ chloramphenicol (AppliChem) to inhibit protein synthesis and allow GFP maturation. *S*.Tm was stained using a human anti-O12 antibody (hSTA5, gift from A. Lanzavecchia, IRB Bellinzona) and a goat anti-human IgG AF647 secondary antibody (Jackson ImmunoResearch). Fluorescence was measured on a Cytoflex flow cytometer (Beckman Colter) using CytExpert v2.5, with gating as described in ref.^140^.

### 16S rRNA gene sequencing

Fecal pellets and cecum contents were snap-frozen and stored at −20 °C. For gDNA extraction, samples were homogenized in RLT buffer with 3 mm metal beads using a TissueLyser II (Qiagen) for 3 min at 30 Hz, followed by two rounds of bead-beating with 0.1 mm zirconia beads. Lysates were processed using the AllPrep DNA/RNA Mini Kit (Qiagen) per the manufacturer’s protocol. The V4 region of the 16S rRNA gene was amplified using primers 806 R (5’-GGACTACNVGGGTWTCTAAT-3’) and 515 F (5’-GTGYCAGCMGCCGCGGTAA-3’)^141,142^, and libraries were multiplexed with Illumina Unique Dual Indexing Primers. Sequencing was performed on an Illumina NextSeq2000 (2 × 300 bp, 450 bp inserts) at BMK, Germany. Reads were trimmed with cutadapt^143^ and processed using the DADA2 pipeline^144^; ASVs were annotated using IDTAXA with the SILVA v138 database^145,146^. Raw data were deposited at the European Nucleotide Archive (ENA), Accession numbers: PRJEB115268; ERP195487

### Carbohydrate utilization of human microbiota

Unless otherwise stated, Bacteroides ovatus ATCC-8483, Bacteroides thetaiotaomicron DSM No.: 2079, Bacteroides vulgatus DSM No.: 1447, Bacteroides uniformis DSM No.: 6597, Clostridum perfringens DSM No.: 11782, Clostridum ramosum DSM No.: 1402, Coprococcus comes ATCC No.: 27758, Dorea formicigenerans DSM No.: 3992, Lactobacillus paracasei ATCC No: SD5275, Odoribacter splanchnicus DSM No.: 20712, Roseburia intestinalis DSM No.: 14610, Ruminococcus gnavus ATCC No.: 29149, S.Tm SB300, and Streptococcus parasanguinis DSM No.: 6778 were routinely grown in YCFA medium (YCFA—Yeast extract-Casein Hydrolysate-Fatty Acids; DSMZ medium 1611) supplemented with 0.5% (w/v) glucose at 37 °C in the anaerobic chamber (Coy Laboratory Products Inc., 2% H2, 12% CO2, and the balance N2). YCFA supplemented with glucose promotes the growth of all the previously mentioned bacterial strains and allows us to replace the carbohydrate source. Therefore, YCFA medium was used as a base medium to examine carbohydrate utilization of human microbial strains. YCFA medium was prepared without a carbohydrate and supplemented with 2x concentrations (1%) of arabinose or glucose (AppliChem, LOT: 64015153. Following supplementation and filtering of the YCFA medium, supplemented and unsupplemented YCFA medium were pipetted into a deep well plate (Thermo Fischer Scientific). Then, 50 µl of medium was distributed to each well of a 96-well u-bottom screening plate (Thermo Fischer Scientific) by using Epmotion 96 (Eppendorf) and stored at −30 °C until use. The human microbial strains were inoculated in YCFA (supplemented with 0.5% glucose) and cultured anaerobically at 37 °C before being subcultured. On the day of subculturing, the stored 96-well plates were thawed, brought into the anaerobic chamber, and pre-reduced. Following O/N incubation, 1.5 mL of each subculture was aliquoted in Eppendorf tubes and centrifuged at RT for 1 min. Supernatants were removed from the Eppendorf tubes, and the bacterial pellets were resuspended in YCFA medium without a carbohydrate source, allow to avoid glucose residues to influence the growth in other carbohydrate sources. OD578 measurements of the resuspended bacteria culture were taken, and the cultures were diluted to an OD578 of 0.02 in YCFA medium (without carbohydrate). Furthermore, 50 µl of the diluted culture per well was used to inoculate the wells of the 96-well plate containing 50 µl of the YCFA supplemented with/without carbohydrate sources, resulting in a starting of a OD578 of 0.01 bacterial culture supplemented with/without 0.5% of carbohydrates.

After inoculation, plates were sealed with breathable membranes (Breathe-Easy) to prevent evaporation and cross-contamination between wells. OD578 was measured hourly for 24 h after 30 s of linear shaking using a microplate spectrophotometer (Epoch2, BioTek, Gen5 software, version 3.05) and an automated microplate stacker (BioStack 4, BioTek) installed in a custom-built incubator (EMBL Mechanical Workshop). At least three biological replicates of each compound-strain combination were tested. Data analysis was performed as previously described (Maier et al.^147^). Briefly, growth curves were blanked, truncated at stationary phase entry, AUCs calculated, and normalized to control growth curves (YCFA without carbohydrate source).

### pH measurements of human microbiota

A photometric pH assay for microplate bacterial cultures was modified to measure the pH of human microbial strains growing in different carbohydrate sources, resulting in a simple and affordable pH measurement protocol (Takao et al., 2020). The following stock solutions were prepared in dimethyl sulfoxide (DMSO; Sigma-Aldrich): 2.5% methyl orange (MO; TCI chemicals), 1% methyl red (MR; Sigma-Aldrich), and 20% bromothymol blue (BTB; TCI chemicals). The following volumes of the stock solutions were then freshly mixed in acetonitrile (AcCN; TCI chemicals) to prepare pH indicator mixtures: 360 µl (0.01%) MO, 480 µl (0.0053%) MR, 180 µl (0.04%) BTB, 5490 µl (0.63 mM) 10 mM NaOH, 5490 µl water, 75000 µl (86.2%) AcCN. The pH standards were simultaneously prepared using a series of McIlvaine buffers (pH 4.0–7.8) (McIlvaine, 1921). A 40 μL aliquot of either buffer/medium/culture and a 90 μL aliquot of AcCN-pH indicator solution were mixed in a 96-well v-bottom plate (Greiner Bio-One). After centrifugation at 500 x *g* for 1 min in a plate centrifuge (Thermo Fisher Scientific), each 60 μL of supernatant was transferred to a well of a black, clear-bottom plate (Greiner Bio-One) and the plates were sealed with breathable membranes (Breathe-Easy) to prevent evaporation. The absorbance values at 540 nm and 620 nm were then measured by a plate reader (Tecan), and Δ(540–620) was calculated. The estimation of the pH of each sample was done by obtaining the calibration curve of the measured pH values against Δ(540–620). At least three biological replicates of each compound-strain combination were tested.

### Growth rates of *B. thetaiotaomicron*, *E. rectale*, and *S*.Tm in varying pH

Data for the dependence of growth rates on pH for *B. thetaiotaomicron* (ATCC 29148) and *Eubacterium rectale* (ATCC 33656) are published in ref. ^148^, and are shown here for comparison. For this study, we have measured the growth of *S*.Tm at different pH values under the same conditions described in ref. ^148^. In short, strains were cultured under anaerobic conditions (10% CO2, 5% H2, 85% N2) in growth medium containing 1% tryptone, 20 mM glucose, 100 mM KH_2_PO_4_/K₂HPO_4_ adjusted to the desired pH, 50 mM NaCl, 0.5 mM CaCl_2_, 0.4 mM MgCl_2_, 50 μM MnCl₂, 50 μM CoCl_2_, 4 μM FeSO_4_, 5 mM cysteine, 20 mM NaHCO_3_, 5 mM Na_2_SO_4_, 20 mM NH_4_Cl, 1.2 mg/L hemin, 1 mg/L menadione, 2 mg/L folinic acid, and 2 mg/L vitamin B12. To reach lower pH values, pH was adjusted using HCl.

### WITS-distribution analysis

Genomic DNA was prepared from overnight cultures, using commercial kits (DNA Mini Kit, Qiagen). qPCR analysis was performed using the FastStart Universal SYBR Green Master Mix (Roche). The thermocycling programs and primers were described previously^149,150^. The population size of each tagged strain was calculated by taking the product of the number of kanamycin-resistant colonies/g feces and the ratio of WITS determined by qPCR.

### Flow cytometry analysis of cecum mucosa immune cell composition

Flow cytometry analysis of cecum mucosa immune cell composition was performed as previously described^81^. Specifically, mice were euthanized, and the cecum was excised and opened longitudinally. To detach epithelial cells, the cecal tissue was cut into small fragments and incubated twice in 14 ml PBS containing 5 mM EDTA, 15 mM HEPES, and 10% FCS for 20 min at 37 °C with gentle agitation. The samples were then washed in RPMI supplemented with 30% FCS and transferred to RPMI containing 1 mg/ml Collagenase VIII (Sigma) and 0.2 mg/ml DNase I (Roche). The tissue fragments were digested for 1 h at 37 °C with mild shaking. Following digestion, the suspension was passed through a 70 μm cell strainer and washed with RPMI. Cells were resuspended in RPMI and gently layered onto a NycoPrep 1.077 gradient (Progen). After centrifugation for 30 min at 400 g, cells at the interphase were collected, rinsed with RPMI containing 15% FCS, and resuspended in PBS with 1% FCS. For intracellular staining, surface marker staining was performed first. Cells were then fixed and permeabilized for 40 min at room temperature in the dark using Foxp3 Fix/Perm buffer (eBioscience/Thermo Fisher). After washing with Permeabilization/Wash buffer (eBioscience/Thermo Fisher), cells were stained for an additional 40 min at room temperature in the dark. Antibodies and dyes used in this study included: CD45 (30-F11, BioLegend), CD4 (RM4-5, Thermo Fisher), Foxp3 (FJK-16s, Thermo Fisher), F4/80 (BM8, BioLegend), CD103 (2E7, BioLegend), MHCII (M5/114.15.2, BioLegend), CD11b (M1/70, BioLegend), CD11c (N418, BioLegend), Sytox Blue (Invitrogen), and Zombie NIR (BioLegend).

### Real-Time qPCR for analysis of transcript levels

Real-Time qPCR was performed as previously described^81^. Mid-segments of the cecum were rinsed in cold sterile PBS to remove luminal contents, shock-frozen in RNAlater (Qiagen) using liquid nitrogen, and stored at −80 °C until RNA isolation. Total RNA was purified with the RNeasy Mini Kit (Qiagen) following the manufacturer’s protocol. RNA concentrations were adjusted to 0.125 µg µl^−1^, and cDNA was synthesized using the RT2 HT First Strand cDNA Kit (Qiagen). Gene expression analysis was performed with custom RT2 Profiler PCR arrays and primer assays (Qiagen) using Universal FastStart SYBR Green Master (ROX; Roche) on a StepOne Plus Real-Time PCR System (Applied Biosystems). The thermal cycling program consisted of an initial denaturation at 95 °C for 14 min, followed by 40 amplification cycles of 94 °C for 15 s, 61 °C for 30 s, and 72 °C for 20 s. Transcript levels were normalized to *actin-b* expression, and a Cq cut-off of 38 cycles was applied.

### Quantification and statistical analysis

#### Statistical analysis

The Mann–Whitney or Wilcoxon test was performed using GraphPad Prism Version 10 for Windows (GraphPad Software, La Jolla, California, USA, www.graphpad.com). Statistical details of each experiment are provided in the corresponding figure legends and in the Source Data File, which contains the underlying raw data. Where sample sizes (*n*) or *P*-values are not explicitly indicated in the figure legends, they are provided in the Source Data File. Horizontal bars in the graphs indicate medians where applicable. *P* values of <0.05 (two-tailed) were considered statistically significant. ^∗^ *P* < 0.05, ^∗∗^ *P* < 0.01, ^∗∗∗^ *P* < 0.001.

### Reporting summary

Further information on research design is available in the Nature Portfolio Reporting Summary linked to this article.

## Supplementary information

Supplemental Information

Supplemental Dataset

Reporting Summary

Transparent Peer Review file

## Source data

Source Data

## Data Availability

The raw sequencing data generated in this study have been deposited in the European Nucleotide Archive (ENA) under accession numbers PRJEB115268 and ERP195487. Source data are provided with this paper (Source Data File). Source data are provided with this paper.

## References

[1] Visconti, A. et al. Interplay between the human gut microbiome and host metabolism. *Nat. Commun.* **10**, 4505 10.1038/s41467-019-12476-z (2019). PMC6776654 31582752

[2] Woelfel, S., Silva, M. S. & Stecher, B. Intestinal colonization resistance in the context of environmental, host, and microbial determinants. *Cell Host Microbe* **32**, 820–836 10.1016/j.chom.2024.05.002 (2024). 38870899

[3] Jordan, C. K. I. & Clarke, T. B. How does the microbiota control systemic innate immunity? *Trends Immunol.* **45**, 94–102 10.1016/j.it.2023.12.002 (2024). 38216387

[4] Qin, J. et al. A metagenome-wide association study of gut microbiota in type 2 diabetes. *Nature* **490**, 55–60 10.1038/nature11450 (2012). 23023125

[5] Bohnhoff, M. & Miller, C. P. Enhanced susceptibility to Salmonella infection in streptomycin-treated mice. *J. Infect. Dis.* **111**, 117–127 10.1093/infdis/111.2.117 (1962). 13968487

[6] Kamada, N., Chen, G. Y., Inohara, N. & Nunez, G. Control of pathogens and pathobionts by the gut microbiota. *Nat. Immunol.* **14**, 685–690 10.1038/ni.2608 (2013). PMC4083503 23778796

[7] Wypych, T. P. & Marsland, B. J. Antibiotics as instigators of microbial dysbiosis: implications for asthma and allergy. *Trends Immunol.* **39**, 697–711 10.1016/j.it.2018.02.008 (2018). 29655522

[8] David, L. A. et al. Diet rapidly and reproducibly alters the human gut microbiome. *Nature* **505**, 559–563 10.1038/nature12820 (2014). PMC3957428 24336217

[9] Marteau, P. & Flourie, B. Tolerance to low-digestible carbohydrates: symptomatology and methods. *Br. J. Nutr.* **85**, S17–S21 10.1079/bjn2000258 (2001). 11321024

[10] Menni, C. et al. Gut microbiome diversity and high-fibre intake are related to lower long-term weight gain. *Int J. Obes. (Lond.)* **41**, 1099–1105 10.1038/ijo.2017.66 (2017). PMC5500185 28286339

[11] Asnicar, F. et al. Microbiome connections with host metabolism and habitual diet from 1,098 deeply phenotyped individuals. *Nat. Med.* **27**, 321–332 10.1038/s41591-020-01183-8 (2021). PMC8353542 33432175

[12] Bisanz, J. E., Upadhyay, V., Turnbaugh, J. A., Ly, K. & Turnbaugh, P. J. Meta-analysis reveals reproducible gut microbiome alterations in response to a high-fat diet. *Cell Host Microbe* **26**, 265–272 e264 10.1016/j.chom.2019.06.013 (2019). PMC6708278 31324413

[13] Agus, A. et al. Western diet induces a shift in microbiota composition, enhancing susceptibility to Adherent-Invasive E. coli infection and intestinal inflammation. *Sci. Rep.* **6**, 19032 10.1038/srep19032 (2016). PMC4705701 26742586

[14] Martinez, K. B., Leone, V. & Chang, E. B. Western diets, gut dysbiosis, and metabolic diseases: are they linked? *Gut Microbes* **8**, 130–142 10.1080/19490976.2016.1270811 (2017). PMC5390820 28059614

[15] Severino, A. et al. The microbiome-driven impact of the Western diet in the development of noncommunicable chronic disorders. *Best. Pr. Res Clin. Gastroenterol.* **72**, 101923 10.1016/j.bpg.2024.101923 (2024). 39645277

[16] Agus, A., Clement, K. & Sokol, H. Gut microbiota-derived metabolites as central regulators in metabolic disorders. *Gut* **70**, 1174–1182 10.1136/gutjnl-2020-323071 (2021). PMC8108286 33272977

[17] Zhang, D. et al. Short-chain fatty acids in diseases. *Cell Commun. Signal* **21**, 212 10.1186/s12964-023-01219-9 (2023). PMC10436623 37596634

[18] Ayakdas, G. & Agagunduz, D. Microbiota-accessible carbohydrates (MACs) as novel gut microbiome modulators in noncommunicable diseases. *Heliyon* **9**, e19888 10.1016/j.heliyon.2023.e19888 (2023). PMC10559293 37809641

[19] Desai, M. S. et al. A dietary fiber-deprived gut microbiota degrades the colonic mucus barrier and enhances pathogen susceptibility. *Cell* **167**, 1339–1353 e1321 10.1016/j.cell.2016.10.043 (2016). PMC5131798 27863247

[20] Wotzka, S. Y., Nguyen, B. D. & Hardt, W. D. Salmonella typhimurium diarrhea reveals basic principles of enteropathogen infection and disease-promoted DNA exchange. *Cell Host Microbe* **21**, 443–454 10.1016/j.chom.2017.03.009 (2017). 28407482

[21] Hryckowian, A. J. et al. Microbiota-accessible carbohydrates suppress Clostridium difficile infection in a murine model. *Nat. Microbiol.* **3**, 662–669 10.1038/s41564-018-0150-6 (2018). PMC6126909 29686297

[22] Mefferd, C. C. et al. A high-fat/high-protein, Atkins-type diet exacerbates Clostridioides (Clostridium) difficile infection in mice, whereas a high-carbohydrate diet protects. *mSystems* **5**, e00765–e00719 (2020). 10.1128/mSystems.00765-19 PMC7018531 32047064

[23] Ramirez-Farias, C. et al. Effect of inulin on the human gut microbiota: stimulation of Bifidobacterium adolescentis and Faecalibacterium prausnitzii. *Br. J. Nutr.* **101**, 541–550 10.1017/S0007114508019880 (2009). 18590586

[24] An, J. et al. Western-style diet impedes colonization and clearance of Citrobacter rodentium. *PLoS Pathog.* **17**, e1009497 10.1371/journal.ppat.1009497 (2021). PMC8049485 33819308

[25] Bovee-Oudenhoven, I. M., ten Bruggencate, S. J., Lettink-Wissink, M. L. & van der Meer, R. Dietary fructo-oligosaccharides and lactulose inhibit intestinal colonisation but stimulate translocation of Salmonella in rats. *Gut* **52**, 1572–1578 10.1136/gut.52.11.1572 (2003). PMC1773861 14570725

[26] Warsi, O., Lundin, E., Lustig, U., Nasvall, J. & Andersson, D. I. Selection for novel metabolic capabilities in Salmonella enterica. *Evolution* **73**, 990–1000 10.1111/evo.13713 (2019). PMC6593847 30848832

[27] Seif, Y. et al. Genome-scale metabolic reconstructions of multiple Salmonella strains reveal serovar-specific metabolic traits. *Nat. Commun.* **9**, 3771 10.1038/s41467-018-06112-5 (2018). PMC6138749 30218022

[28] Gutnick, D., Calvo, J. M., Klopotowski, T. & Ames, B. N. Compounds which serve as the sole source of carbon or nitrogen for Salmonella typhimurium LT-2. *J. Bacteriol.* **100**, 215–219 10.1128/jb.100.1.215-219.1969 (1969). PMC315380 4898986

[29] Gul, E. et al. Differences in carbon metabolic capacity fuel co-existence and plasmid transfer between Salmonella strains in the mouse gut. *Cell Host Microbe* **31**, 1140–1153 e1143 10.1016/j.chom.2023.05.029 (2023). 37348498

[30] Eberl, C. et al. E. coli enhance colonization resistance against Salmonella Typhimurium by competing for galactitol, a context-dependent limiting carbon source. *Cell Host Microbe* **29**, 1680–1692 e1687 10.1016/j.chom.2021.09.004 (2021). 34610296

[31] Tuck, C. J. et al. Nutritional profile of rodent diets impacts experimental reproducibility in microbiome preclinical research. *Sci. Rep.* **10**, 17784 10.1038/s41598-020-74460-8 (2020). PMC7575541 33082369

[32] Barthel, M. et al. Pretreatment of mice with streptomycin provides a Salmonella enterica serovar Typhimurium colitis model that allows analysis of both pathogen and host. *Infect. Immun.* **71**, 2839–2858 10.1128/IAI.71.5.2839-2858.2003 (2003). PMC153285 12704158

[33] Koepsell, H. Glucose transporters in the small intestine in health and disease. *Pflug. Arch.* **472**, 1207–1248 10.1007/s00424-020-02439-5 (2020). PMC7462918 32829466

[34] Furter, M., Sellin, M. E., Hansson, G. C. & Hardt, W. D. Mucus architecture and near-surface swimming affect distinct Salmonella typhimurium infection patterns along the murine intestinal tract. *Cell Rep.* **27**, 2665–2678 e2663 10.1016/j.celrep.2019.04.106 (2019). PMC6547020 31141690

[35] Seri, K. et al. L-arabinose selectively inhibits intestinal sucrase in an uncompetitive manner and suppresses glycemic response after sucrose ingestion in animals. *Metabolism* **45**, 1368–1374 10.1016/s0026-0495(96)90117-1 (1996). 8931641

[36] Lee, J. H., Al-Zarban, S. & Wilcox, G. Genetic characterization of the araE gene in Salmonella typhimurium lt2. *J. Bacteriol.* **146**, 298–304 10.1128/jb.146.1.298-304.1981 (1981). PMC217082 7012118

[37] Lee, J. H., Russo, R. J., Heffernan, L. & Wilcox, G. Regulation of L-arabinose transport in Salmonella typhimurium LT2. *Mol. Gen. Genet.* **185**, 136–141 10.1007/BF00333803 (1982). 6283309

[38] Old, D. & Mortlock, R. P. The metabolism of D-arabinose by Salmonella typhimurium. *J. Gen. Microbiol.* **101**, 341–344 10.1099/00221287-101-2-341 (1977). 200704

[39] Koropatkin, N. M., Cameron, E. A. & Martens, E. C. How glycan metabolism shapes the human gut microbiota. *Nat. Rev. Microbiol.* **10**, 323–335 10.1038/nrmicro2746 (2012). PMC4005082 22491358

[40] Flint, H. J., Scott, K. P., Duncan, S. H., Louis, P. & Forano, E. Microbial degradation of complex carbohydrates in the gut. *Gut Microbes* **3**, 289–306 10.4161/gmic.19897 (2012). PMC3463488 22572875

[41] Arzamasov, A. A., van Sinderen, D. & Rodionov, D. A. Comparative genomics reveals the regulatory complexity of bifidobacterial arabinose and arabino-oligosaccharide utilization. *Front. Microbiol.* **9**, 776 10.3389/fmicb.2018.00776 (2018). PMC5928203 29740413

[42] Zhang, L. et al. Ribulokinase and transcriptional regulation of arabinose metabolism in Clostridium acetobutylicum. *J. Bacteriol.* **194**, 1055–1064 10.1128/JB.06241-11 (2012). PMC3294803 22194461

[43] Glowacki, R. W. P. & Martens, E. C. If you eat it, or secrete it, they will grow: the expanding list of nutrients utilized by human gut bacteria. *J. Bacteriol.* **203**, fiac038 10.1128/JB.00481-20 (2020). PMC8092160 33168637

[44] Firrman, J. et al. The impact of environmental pH on the gut microbiota community structure and short-chain fatty acid production. *FEMS Microbiol. Ecol.* **98**, e00481–20 10.1093/femsec/fiac038 (2022). 35383853

[45] Duncan, S. H., Louis, P., Thomson, J. M. & Flint, H. J. The role of pH in determining the species composition of the human colonic microbiota. *Environ. Microbiol.* **11**, 2112–2122 10.1111/j.1462-2920.2009.01931.x (2009). 19397676

[46] Walker, A. W., Duncan, S. H., McWilliam Leitch, E. C., Child, M. W. & Flint, H. J. pH and peptide supply can radically alter bacterial populations and short-chain fatty acid ratios within microbial communities from the human colon. *Appl. Environ. Microbiol.* **71**, 3692–3700 10.1128/AEM.71.7.3692-3700.2005 (2005). PMC1169066 16000778

[47] Jacobson, A. et al. A gut commensal-produced metabolite mediates colonization resistance to salmonella infection. *Cell Host Microbe* **24**, 296–307 e297 10.1016/j.chom.2018.07.002 (2018). PMC6223613 30057174

[48] Madeira, J. V. et al. in *Biotechnology of Microbial Enzymes* (ed Goutam Brahmachari) 475–511 (Academic Press, 2017).

[49] Macfarlane, G. T. & Macfarlane, S. Bacteria, colonic fermentation, and gastrointestinal health. *J. AOAC Int* **95**, 50–60 10.5740/jaoacint.sge_macfarlane (2012). 22468341

[50] Tomioka, S. et al. Cooperative action of gut-microbiota-accessible carbohydrates improves host metabolic function. *Cell Rep.* **40**, 111087 10.1016/j.celrep.2022.111087 (2022). 35858544

[51] Ciani, M., Comitini, F. & Mannazzu, I. In *Encyclopedia of Ecology* (eds Sven Erik Jørgensen & Brian D. Fath) 1548–1557 (Academic Press, 2008).

[52] Pickard, J. M. et al. Dietary amino acids regulate Salmonella colonization via microbiota-dependent mechanisms in the mouse gut. *Nat. Commun.* **16**, 4225 10.1038/s41467-025-59706-1 (2025). PMC12058977 40335509

[53] Pickard, J.M. et al. Dietary amino acids regulate Salmonella colonization via microbiota-dependent mechanisms in the mouse gut. *Nat Commun* **16**, 4225 10.1038/s41467-025-59706-1 (2025). PMC12058977 40335509

[54] Coburn, B., Grassl, G. A. & Finlay, B. B. Salmonella, the host and disease: a brief review. *Immunol. Cell Biol.* **85**, 112–118 10.1038/sj.icb.7100007 (2007). 17146467

[55] Galan, J. E. & Curtiss, R. 3rd. Cloning and molecular characterization of genes whose products allow Salmonella typhimurium to penetrate tissue culture cells. *Proc. Natl. Acad. Sci. USA* **86**, 6383–6387 10.1073/pnas.86.16.6383 (1989). PMC297844 2548211

[56] Hapfelmeier, S. et al. The Salmonella pathogenicity island (SPI)-2 and SPI-1 type III secretion systems allow Salmonella serovar typhimurium to trigger colitis via MyD88-dependent and MyD88-independent mechanisms. *J. Immunol.* **174**, 1675–1685 10.4049/jimmunol.174.3.1675 (2005). 15661931

[57] Hensel, M. et al. Simultaneous identification of bacterial virulence genes by negative selection. *Science* **269**, 400–403 10.1126/science.7618105 (1995). 7618105

[58] Swart, A. L. & Hensel, M. Interactions of Salmonella enterica with dendritic cells. *Virulence* **3**, 660–667 10.4161/viru.22761 (2012). PMC3545948 23221476

[59] Sturm, A. et al. The cost of virulence: retarded growth of Salmonella Typhimurium cells expressing type III secretion system 1. *PLoS Pathog.* **7**, e1002143 10.1371/journal.ppat.1002143 (2011). PMC3145796 21829349

[60] Hapfelmeier, S. et al. Role of the Salmonella pathogenicity island 1 effector proteins SipA, SopB, SopE, and SopE2 in Salmonella enterica subspecies 1 serovar Typhimurium colitis in streptomycin-pretreated mice. *Infect. Immun.* **72**, 795–809 10.1128/IAI.72.2.795-809.2004 (2004). PMC321604 14742523

[61] Fattinger, S. A. et al. Salmonella Typhimurium discreet-invasion of the murine gut absorptive epithelium. *PLoS Pathog.* **16**, e1008503 10.1371/journal.ppat.1008503 (2020). PMC7224572 32365138

[62] Raffatellu, M. et al. SipA, SopA, SopB, SopD, and SopE2 contribute to Salmonella enterica serotype typhimurium invasion of epithelial cells. *Infect. Immun.* **73**, 146–154 10.1128/IAI.73.1.146-154.2005 (2005). PMC538951 15618149

[63] Lopez, C. A., Rivera-Chavez, F., Byndloss, M. X. & Baumler, A. J. The periplasmic nitrate reductase NapABC supports luminal growth of Salmonella enterica serovar typhimurium during colitis. *Infect. Immun.* **83**, 3470–3478 10.1128/IAI.00351-15 (2015). PMC4534643 26099579

[64] Winter, S. E. et al. Gut inflammation provides a respiratory electron acceptor for Salmonella. *Nature* **467**, 426–429 10.1038/nature09415 (2010). PMC2946174 20864996

[65] Stecher, B. et al. Like will to like: abundances of closely related species can predict susceptibility to intestinal colonization by pathogenic and commensal bacteria. *PLoS Pathog.* **6**, e1000711 10.1371/journal.ppat.1000711 (2010). PMC2796170 20062525

[66] Nguyen, B. D. et al. Salmonella Typhimurium screen identifies shifts in mixed-acid fermentation during gut colonization. *Cell Host Microbe* **32**, 1758–1773 e1754 10.1016/j.chom.2024.08.015 (2024). 39293436

[67] Stecher, B. et al. Salmonella enterica serovar typhimurium exploits inflammation to compete with the intestinal microbiota. *PLoS Biol.* **5**, 2177–2189 10.1371/journal.pbio.0050244 (2007). PMC1951780 17760501

[68] Bakkeren, E. et al. Salmonella persisters promote the spread of antibiotic resistance plasmids in the gut. *Nature* **573**, 276–280 10.1038/s41586-019-1521-8 (2019). PMC6744281 31485077

[69] Ashkar, A. A., Reid, S., Verdu, E. F., Zhang, K. & Coombes, B. K. Interleukin-15 and NK1.1+ cells provide innate protection against acute Salmonella enterica serovar Typhimurium infection in the gut and in systemic tissues. *Infect. Immun.* **77**, 214–222 10.1128/IAI.01066-08 (2009). PMC2612292 19015253

[70] Kupz, A. et al. Contribution of Thy1+ NK cells to protective IFN-gamma production during Salmonella typhimurium infections. *Proc. Natl. Acad. Sci. USA* **110**, 2252–2257 10.1073/pnas.1222047110 (2013). PMC3568339 23345426

[71] Muller, A. A. et al. An NK cell perforin response elicited via IL-18 controls mucosal inflammation kinetics during Salmonella gut infection. *PLoS Pathog.* **12**, e1005723 10.1371/journal.ppat.1005723 (2016). PMC4920399 27341123

[72] Harrington, L., Srikanth, C. V., Antony, R., Shi, H. N. & Cherayil, B. J. A role for natural killer cells in intestinal inflammation caused by infection with Salmonella enterica serovar Typhimurium. *FEMS Immunol. Med. Microbiol.* **51**, 372–380 10.1111/j.1574-695X.2007.00313.x (2007). PMC3205980 17727655

[73] Simpson, R. B., Zhou, B. & Naumova, E. N. Seasonal synchronization of foodborne outbreaks in the United States, 1996-2017. *Sci. Rep.* **10**, 17500 10.1038/s41598-020-74435-9 (2020). PMC7562704 33060743

[74] Naumova, E. N. et al. Seasonality in six enterically transmitted diseases and ambient temperature. *Epidemiol. Infect.* **135**, 281–292 10.1017/S0950268806006698 (2007). PMC2870561 17291363

[75] Tobolowsky, F. A., Cui, Z., Hoekstra, R. M. & Bruce, B. B. Salmonella serotypes associated with illnesses after the Thanksgiving holiday, United States, 1998–2018. *Emerg. Infect. Dis.* **28**, 210–213 10.3201/eid2801.211986 (2022). PMC8714195 34932459

[76] Federal Office of Public Health (FOPH). Infectious Diseases Dashboard (IDD): Mandatory reporting system. Swiss Confederation. https://www.idd.bag.admin.ch/en/diseases/salmonellosis/statistic (2026).

[77] Lake, I. R. et al. A re-evaluation of the impact of temperature and climate change on foodborne illness. *Epidemiol. Infect.* **137**, 1538–1547 10.1017/S0950268809002477 (2009). 19371450

[78] Misiou, O. & Koutsoumanis, K. Climate change and its implications for food safety and spoilage. *Trends Food Sci. Technol.* **126**, 142–152 10.1016/j.tifs.2021.03.031 (2022).

[79] Wilson, E. Foodborne illness and seasonality related to mobile food sources at festivals and group gatherings in the state of Georgia. *J. Environ. Health* **77**, 54 (2015). 8-11; quiz. 25796696

[80] Kreuzer, M. & Hardt, W. D. How food affects colonization resistance against enteropathogenic bacteria. *Annu Rev. Microbiol.* **74**, 787–813 10.1146/annurev-micro-020420-013457 (2020). 32692613

[81] Wotzka, S. Y. et al. Escherichia coli limits Salmonella Typhimurium infections after diet shifts and fat-mediated microbiota perturbation in mice. *Nat. Microbiol.* **4**, 2164–2174 10.1038/s41564-019-0568-5 (2019). PMC6881180 31591555

[82] Kieboom, J. & Abee, T. Arginine-dependent acid resistance in Salmonella enterica serovar Typhimurium. *J. Bacteriol.* **188**, 5650–5653 10.1128/JB.00323-06 (2006). PMC1540025 16855258

[83] Viala, J. P. et al. Sensing and adaptation to low pH mediated by inducible amino acid decarboxylases in Salmonella. *PLoS ONE* **6**, e22397 10.1371/journal.pone.0022397 (2011). PMC3143133 21799843

[84] Foster, J. W. The acid tolerance response of Salmonella typhimurium involves the transient synthesis of key acid shock proteins. *J. Bacteriol.* **175**, 1981–1987 10.1128/jb.175.7.1981-1987.1993 (1993). PMC204281 8458840

[85] Foster, J. W. Escherichia coli acid resistance: tales of an amateur acidophile. *Nat. Rev. Microbiol.* **2**, 898–907 10.1038/nrmicro1021 (2004). 15494746

[86] Ohkuma, S. & Poole, B. Fluorescence probe measurement of the intralysosomal pH in living cells and the perturbation of pH by various agents. *Proc. Natl. Acad. Sci. USA* **75**, 3327–3331 10.1073/pnas.75.7.3327 (1978). PMC392768 28524

[87] Drecktrah, D., Knodler, L. A., Ireland, R. & Steele-Mortimer, O. The mechanism of Salmonella entry determines the vacuolar environment and intracellular gene expression. *Traffic* **7**, 39–51 10.1111/j.1600-0854.2005.00360.x (2006). 16445685

[88] Alpuche Aranda, C. M., Swanson, J. A., Loomis, W. P. & Miller, S. I. Salmonella typhimurium activates virulence gene transcription within acidified macrophage phagosomes. *Proc. Natl. Acad. Sci. USA* **89**, 10079–10083 10.1073/pnas.89.21.10079 (1992). PMC50281 1438196

[89] Rathman, M., Sjaastad, M. D. & Falkow, S. Acidification of phagosomes containing Salmonella typhimurium in murine macrophages. *Infect. Immun.* **64**, 2765–2773 10.1128/iai.64.7.2765-2773.1996 (1996). PMC174137 8698506

[90] Baik, H. S., Bearson, S., Dunbar, S. & Foster, J. W. The acid tolerance response of Salmonella typhimurium provides protection against organic acids. *Microbiology* **142**, 3195–3200 10.1099/13500872-142-11-3195 (1996). 8969516

[91] Ruddle, S. J., Massis, L. M., Cutter, A. C. & Monack, D. M. Salmonella-liberated dietary L-arabinose promotes expansion in superspreaders. *Cell Host Microbe* **31**, 405–417 e405 10.1016/j.chom.2023.01.017 (2023). PMC10016319 36812913

[92] Yu, J. et al. Dietary L-arabinose-induced gut dysbiosis exacerbates Salmonella infection outcome. *mSystems* **9**, e0052224 10.1128/msystems.00522-24 (2024). PMC11334454 38980058

[93] Lopez-Garrido, J., Puerta-Fernandez, E., Cota, I. & Casadesus, J. Virulence gene regulation by L-arabinose in Salmonella enterica. *Genetics* **200**, 807–819 10.1534/genetics.115.178103 (2015). PMC4512545 25991823

[94] Lawhon, S. D., Maurer, R., Suyemoto, M. & Altier, C. Intestinal short-chain fatty acids alter Salmonella typhimurium invasion gene expression and virulence through BarA/SirA. *Mol. Microbiol.* **46**, 1451–1464 10.1046/j.1365-2958.2002.03268.x (2002). 12453229

[95] Hockenberry, A. M. et al. Microbiota-derived metabolites inhibit Salmonella virulent subpopulation development by acting on single-cell behaviors. *Proc. Natl. Acad. Sci. USA* **118**, e2103027118 (2021). 10.1073/pnas.2103027118 PMC8346864 34330831

[96] Gantois, I. et al. Butyrate specifically down-regulates Salmonella pathogenicity island 1 gene expression. *Appl. Environ. Microbiol.* **72**, 946–949 10.1128/AEM.72.1.946-949.2006 (2006). PMC1352287 16391141

[97] Deiwick, J., Nikolaus, T., Erdogan, S. & Hensel, M. Environmental regulation of Salmonella pathogenicity island 2 gene expression. *Mol. Microbiol.* **31**, 1759–1773 10.1046/j.1365-2958.1999.01312.x (1999). 10209748

[98] Jiang, L. et al. Salmonella Typhimurium reprograms macrophage metabolism via T3SS effector SopE2 to promote intracellular replication and virulence. *Nat. Commun.* **12**, 879 10.1038/s41467-021-21186-4 (2021). PMC7873081 33563986

[99] Rosenberg, G. et al. Host succinate is an activation signal for Salmonella virulence during intracellular infection. *Science* **371**, 400–405 10.1126/science.aba8026 (2021). 33479153

[100] Coombes, B. K., Brown, N. F., Valdez, Y., Brumell, J. H. & Finlay, B. B. Expression and secretion of Salmonella pathogenicity island-2 virulence genes in response to acidification exhibit differential requirements of a functional type III secretion apparatus and SsaL. *J. Biol. Chem.* **279**, 49804–49815 10.1074/jbc.M404299200 (2004). 15383528

[101] Brown, N. F. et al. Salmonella pathogenicity island 2 is expressed prior to penetrating the intestine. *PLoS Pathog.* **1**, e32 10.1371/journal.ppat.0010032 (2005). PMC1287911 16304611

[102] Cirillo, D. M., Valdivia, R. H., Monack, D. M. & Falkow, S. Macrophage-dependent induction of the Salmonella pathogenicity island 2 type III secretion system and its role in intracellular survival. *Mol. Microbiol.* **30**, 175–188 10.1046/j.1365-2958.1998.01048.x (1998). 9786194

[103] Osborne, S. E. & Coombes, B. K. Transcriptional priming of Salmonella pathogenicity Island-2 precedes cellular invasion. *PLoS ONE* **6**, e21648 10.1371/journal.pone.0021648 (2011). PMC3125303 21738750

[104] Jones, B. D., Ghori, N. & Falkow, S. Salmonella typhimurium initiates murine infection by penetrating and destroying the specialized epithelial M cells of the Peyer’s patches. *J. Exp. Med.* **180**, 15–23 10.1084/jem.180.1.15 (1994). PMC2191576 8006579

[105] Sansonetti, P. J. & Phalipon, A. M cells as ports of entry for enteroinvasive pathogens: mechanisms of interaction, consequences for the disease process. *Semin Immunol.* **11**, 193–203 10.1006/smim.1999.0175 (1999). 10381865

[106] Tahoun, A. et al. Salmonella transforms follicle-associated epithelial cells into M cells to promote intestinal invasion. *Cell Host Microbe* **12**, 645–656 10.1016/j.chom.2012.10.009 (2012). 23159054

[107] Kishikawa, S. et al. Allograft inflammatory factor 1 is a regulator of transcytosis in M cells. *Nat. Commun.* **8**, 14509 10.1038/ncomms14509 (2017). PMC5322540 28224999

[108] Ragnarsson, E. G. et al. Yersinia pseudotuberculosis induces transcytosis of nanoparticles across human intestinal villus epithelium via invasin-dependent macropinocytosis. *Lab Invest.* **88**, 1215–1226 10.1038/labinvest.2008.86 (2008). 18810251

[109] Owen, R. L., Pierce, N. F., Apple, R. T., Cray & Cray, W. C. Jr. cell transport of Vibrio cholerae from the intestinal lumen into Peyer’s patches: a mechanism for antigen sampling and for microbial transepithelial migration. *J. Infect. Dis.* **153**, 1108–1118 10.1093/infdis/153.6.1108 (1986). 2422297

[110] Gopinath, A., Allen, T. A., Bridgwater, C. J., Young, C. M. & Worley, M. J. The Salmonella type III effector SpvC triggers the reverse transmigration of infected cells into the bloodstream. *PLoS ONE* **14**, e0226126 10.1371/journal.pone.0226126 (2019). PMC6901223 31815949

[111] Lim, C. H. et al. Independent bottlenecks characterize colonization of systemic compartments and gut lymphoid tissue by Salmonella. *PLoS Pathog.* **10**, e1004270 10.1371/journal.ppat.1004270 (2014). PMC4117638 25079958

[112] Armitage, S. A., Genersch, E., McMahon, D. P., Rafaluk-Mohr, C. & Rolff, J. Tripartite interactions: how immunity, microbiota and pathogens interact and affect pathogen virulence evolution. *Curr. Opin. Insect Sci.* **50**, 100871 10.1016/j.cois.2021.12.011 (2022). 34999035

[113] Bohm, L. et al. The yeast form of the fungus Candida albicans promotes persistence in the gut of gnotobiotic mice. *PLoS Pathog.* **13**, e1006699 10.1371/journal.ppat.1006699 (2017). PMC5673237 29069103

[114] Tso, G. H. W. et al. Experimental evolution of a fungal pathogen into a gut symbiont. *Science* **362**, 589–595 10.1126/science.aat0537 (2018). 30385579

[115] Gul, E. et al. The microbiota conditions a gut milieu that selects for wild-type Salmonella Typhimurium virulence. *PLoS Biol.* **21**, e3002253 10.1371/journal.pbio.3002253 (2023). PMC10499267 37651408

[116] Gul, E. et al. Salmonella T3SS-2 virulence enhances gut-luminal colonization by enabling chemotaxis-dependent exploitation of intestinal inflammation. *Cell Rep.* **43**, 113925 10.1016/j.celrep.2024.113925 (2024). 38460128

[117] Diard, M. et al. Stabilization of cooperative virulence by the expression of an avirulent phenotype. *Nature* **494**, 353–356 10.1038/nature11913 (2013). 23426324

[118] Pol, K., Puhlmann, M. L. & Mars, M. Efficacy of L-arabinose in lowering glycemic and insulinemic responses: the modifying effect of starch and fat. *Foods* **11**, 157 10.3390/foods11020157 (2022). PMC8774789 35053889

[119] Bloom, P. P. & Tapper, E. B. Lactulose in cirrhosis: current understanding of efficacy, mechanism, and practical considerations. *Hepatol. Commun.* **7**, e0295 10.1097/HC9.0000000000000295 (2023). PMC10578757 37820287

[120] Tungland, B. in *Human Microbiota in Health and Disease* (ed Bryan Tungland) 289–348 (Academic Press, 2018).

[121] Grembecka, M. Sugar alcohols—their role in the modern world of sweeteners: a review. *Eur. Food Res. Technol.* **241**, 1–14 10.1007/s00217-015-2437-7 (2015).

[122] Wotzka, S. Y. et al. Microbiota stability in healthy individuals after single-dose lactulose challenge: a randomized controlled study. *PLoS ONE* **13**, e0206214 10.1371/journal.pone.0206214 (2018). PMC6201941 30359438

[123] Vidyasagar, S., Barmeyer, C., Geibel, J., Binder, H. J. & Rajendran, V. M. Role of short-chain fatty acids in colonic HCO(3) secretion. *Am. J. Physiol. Gastrointest. Liver Physiol.* **288**, G1217–G1226 10.1152/ajpgi.00415.2004 (2005). 15677553

[124] Becker, H. M. & Seidler, U. E. Bicarbonate secretion and acid/base sensing by the intestine. *Pflug. Arch.* **476**, 593–610 10.1007/s00424-024-02914-3 (2024). PMC11006743 38374228

[125] Kini, A. et al. Slc26a3 deletion alters pH-microclimate, mucin biosynthesis, microbiome composition and increases the TNFalpha expression in murine colon. *Acta Physiol. (Oxf.)* **230**, e13498 10.1111/apha.13498 (2020). 32415725

[126] Asano, K. et al. Contribution of susceptibility variants at FCGR2A and 13q12 to the risk of relapse among Japanese patients with ulcerative colitis. *J. Gastroenterol.* **50**, 1094–1102 10.1007/s00535-015-1062-3 (2015). 25787843

[127] Fujii, T. et al. Assessment of the family history of patients with ulcerative colitis at a single center in Japan. *J. Pediatr. Gastroenterol. Nutr.* **63**, 512–515 10.1097/MPG.0000000000001275 (2016). 27352080

[128] Shao, X. X. et al. Association of ulcerative colitis with solute-linked carrier family 26 member A3 gene polymorphisms and its expression in colonic tissues in Chinese patients. *Int J. Colorectal Dis.* **33**, 1169–1172 10.1007/s00384-018-3097-4 (2018). 29855681

[129] Fooks, L. J. & Gibson, G. R. In vitro investigations of the effect of probiotics and prebiotics on selected human intestinal pathogens. *FEMS Microbiol. Ecol.* **39**, 67–75 10.1111/j.1574-6941.2002.tb00907.x (2002). 19709185

[130] Petersen, A. et al. Some putative prebiotics increase the severity of Salmonella enterica serovar Typhimurium infection in mice. *BMC Microbiol.* **9**, 245 10.1186/1471-2180-9-245 (2009). PMC2789089 19948011

[131] Ten Bruggencate, S. J., Bovee-Oudenhoven, I. M., Lettink-Wissink, M. L. & Van der Meer, R. Dietary fructo-oligosaccharides dose-dependently increase translocation of Salmonella in rats. *J. Nutr.* **133**, 2313–2318 10.1093/jn/133.7.2313 (2003). 12840199

[132] Hoiseth, S. K. & Stocker, B. A. Aromatic-dependent *Salmonella typhimurium* are non-virulent and effective as live vaccines. *Nature* **291**, 238–239 (1981). 10.1038/291238a0 7015147

[133] Kaniga, K., Bossio, J. C. & Galan, J. E. The Salmonella typhimurium invasion genes invF and invG encode homologues of the AraC and PulD family of proteins. *Mol. Microbiol.* **13**, 555–568 (1994). 10.1111/j.1365-2958.1994.tb00450.x 7997169

[134] Datsenko, K. A. & Wanner, B. L. One-step inactivation of chromosomal genes in Escherichia coli K-12 using PCR products. *Proc. Natl. Acad. Sci. USA* **97**, 6640–6645 10.1073/pnas.120163297 (2000). PMC18686 10829079

[135] Bertani, G. Studies on lysogenesis. I. The mode of phage liberation by lysogenic Escherichia coli. *J. Bacteriol.* **62**, 293–300 10.1128/jb.62.3.293-300.1951 (1951). PMC386127 14888646

[136] Wittekind, D. Traditional staining for routine diagnostic pathology, including the role of tannic acid. 1. Value and limitations of the hematoxylin-eosin stain. *Biotech. Histochem.* **78**, 261–270 10.1080/10520290310001633725 (2003). 14989644

[137] Slack, E. et al. Innate and adaptive immunity cooperate flexibly to maintain host-microbiota mutualism. *Science* **325**, 617–620 10.1126/science.1172747 (2009). PMC3730530 19644121

[138] Han, J., Lin, K., Sequeira, C. & Borchers, C. H. An isotope-labeled chemical derivatization method for the quantitation of short-chain fatty acids in human feces by liquid chromatography-tandem mass spectrometry. *Anal. Chim. Acta* **854**, 86–94 10.1016/j.aca.2014.11.015 (2015). 25479871

[139] Mulleder, M. et al. Functional metabolomics describes the yeast biosynthetic regulome. *Cell* **167**, 553–565 e512 10.1016/j.cell.2016.09.007 (2016). PMC5055083 27693354

[140] Kroon, S. et al. Sublethal systemic LPS in mice enables gut-luminal pathogens to bloom through oxygen species-mediated microbiota inhibition. *Nat. Commun.* **16**, 2760 10.1038/s41467-025-57979-0 (2025). PMC11926250 40113753

[141] Apprill, A., Weber, L., Parsons, R. & McNally, S. Minor revision to V4 region SSU rRNA 806R gene primer greatly increases detection of SAR11 bacterioplankton. *Aquat. Microb. Ecol.* **75**, 129–137 (2015).

[142] Parada, A. E., Needham, D. M. & Fuhrman, J. A. Every base matters: assessing small subunit rRNA primers for marine microbiomes with mock communities, time series and global field samples. *Environ. Microbiol.* **18**, 1403–1414 10.1111/1462-2920.13023 (2016). 26271760

[143] Martin, M. Cutadapt removes adapter sequences from high-throughput sequencing reads. *EMBnet.journal**;**Vol 17*, *No 1: Next Generation Sequencing Data AnalysisDO**-*10.14806/ej.17.1.200 (2011).

[144] Callahan, B. J. et al. DADA2: High-resolution sample inference from Illumina amplicon data. *Nat. Methods* **13**, 581–583 10.1038/nmeth.3869 (2016). PMC4927377 27214047

[145] Murali, A., Bhargava, A. & Wright, E. S. IDTAXA: a novel approach for accurate taxonomic classification of microbiome sequences. *Microbiome* **6**, 140 10.1186/s40168-018-0521-5 (2018). PMC6085705 30092815

[146] Quast, C. et al. The SILVA ribosomal RNA gene database project: improved data processing and web-based tools. *Nucleic Acids Res.* **41**, D590–D596 10.1093/nar/gks1219 (2013). PMC3531112 23193283

[147] Maier, L. et al. Extensive impact of non-antibiotic drugs on human gut bacteria. *Nature.* **555**, 623–628. 10.1038/nature25979 (2018). PMC6108420 29555994

[148] Cremer, J., Arnoldini, M. & Hwa, T. Effect of water flow and chemical environment on microbiota growth and composition in the human colon. *Proc. Natl. Acad. Sci. USA* **114**, 6438–6443 10.1073/pnas.1619598114 (2017). PMC5488924 28588144

[149] Maier, L. et al. Granulocytes impose a tight bottleneck upon the gut luminal pathogen population during Salmonella typhimurium colitis. *PLoS Pathog.* **10**, e1004557 10.1371/journal.ppat.1004557 (2014). PMC4270771 25522364

[150] Grant, A. J. et al. Modelling within-host spatiotemporal dynamics of invasive bacterial disease. *PLoS Biol.* **6**, e74 10.1371/journal.pbio.0060074 (2008). PMC2288627 18399718

