# Supplementary material for: Overconsumption of low-digestible sugars can increase susceptibility to Salmonella Typhimurium infection
Source: Nat Commun. 2026 Nov 3;17:11372. doi: 10.1038/s41467-026-78218-0 (PMC13731649; doi:10.1038/s41467-026-78218-0)
Supplement: Supplementary file 1 [file 41467_2026_78218_MOESM1_ESM.pdf]

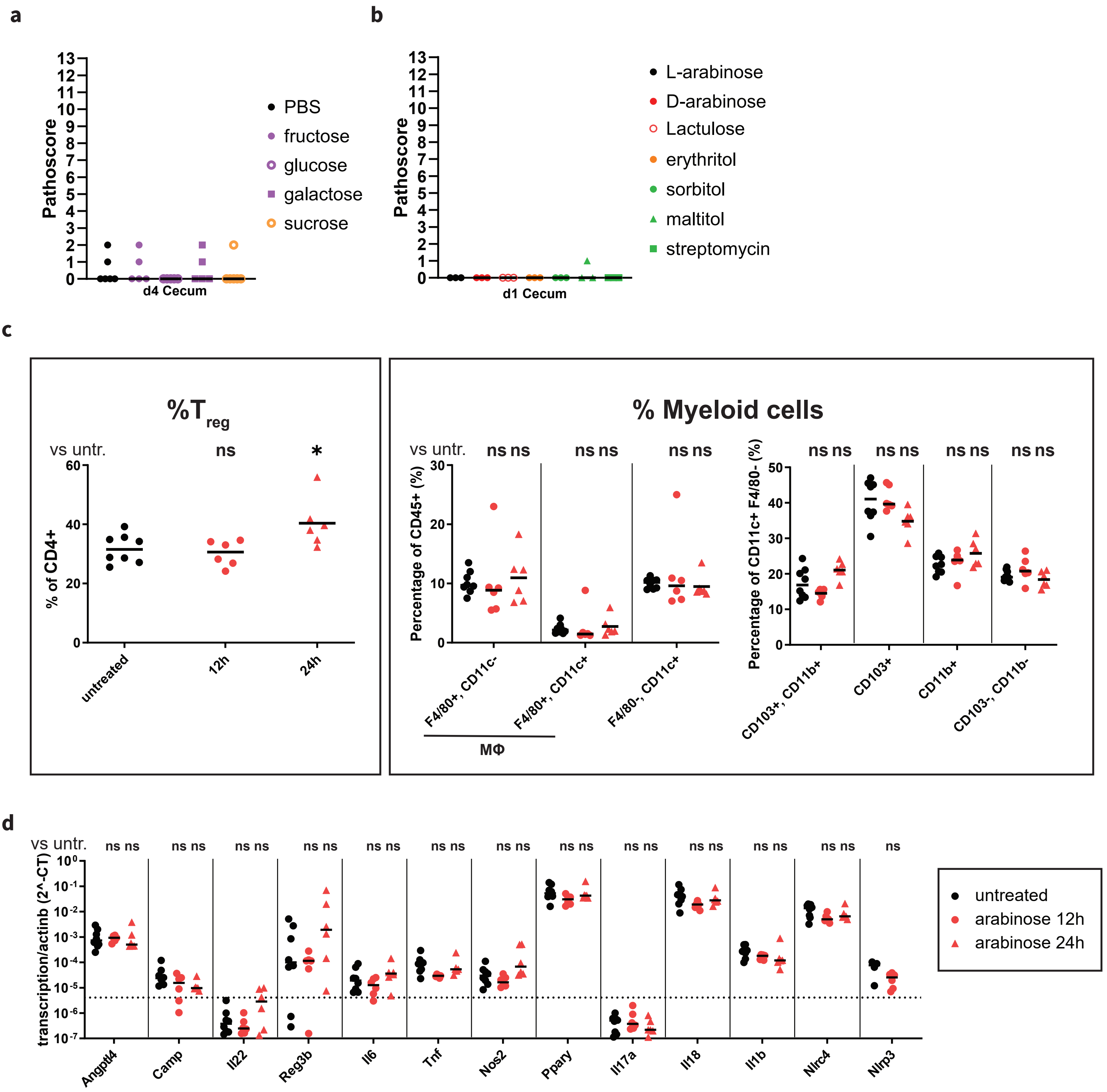

Supplementary Figure 1. Assessment of inflammatory responses in uninfected mice following LDS overconsumption. Related to Figure 1. (a) Histopathological analysis corresponding to Figure 1F. (b) Histopathological sections from uninfected mice one day after administration of two doses of 132 mg of the indicated LDS. (c–d) Cecal tissue samples were collected 12 or 24 hours after administration of two doses of 132 mg arabinose, or from untreated controls. (c) Regulatory T cells (Tregs) and myeloid cells in cecal tissue. Treg fractions (of CD45⁺ CD4⁺ live cells) and myeloid cell fractions in the cecal lamina propria were analyzed. Cells were pre-gated on CD45⁺ live cells. MΦ, macrophages. (d) Transcriptional analysis of cecal tissue by qPCR for selected immune cell markers and cytokines. The Mann-Whitney U test was used for statistical analysis; ns, not significant.

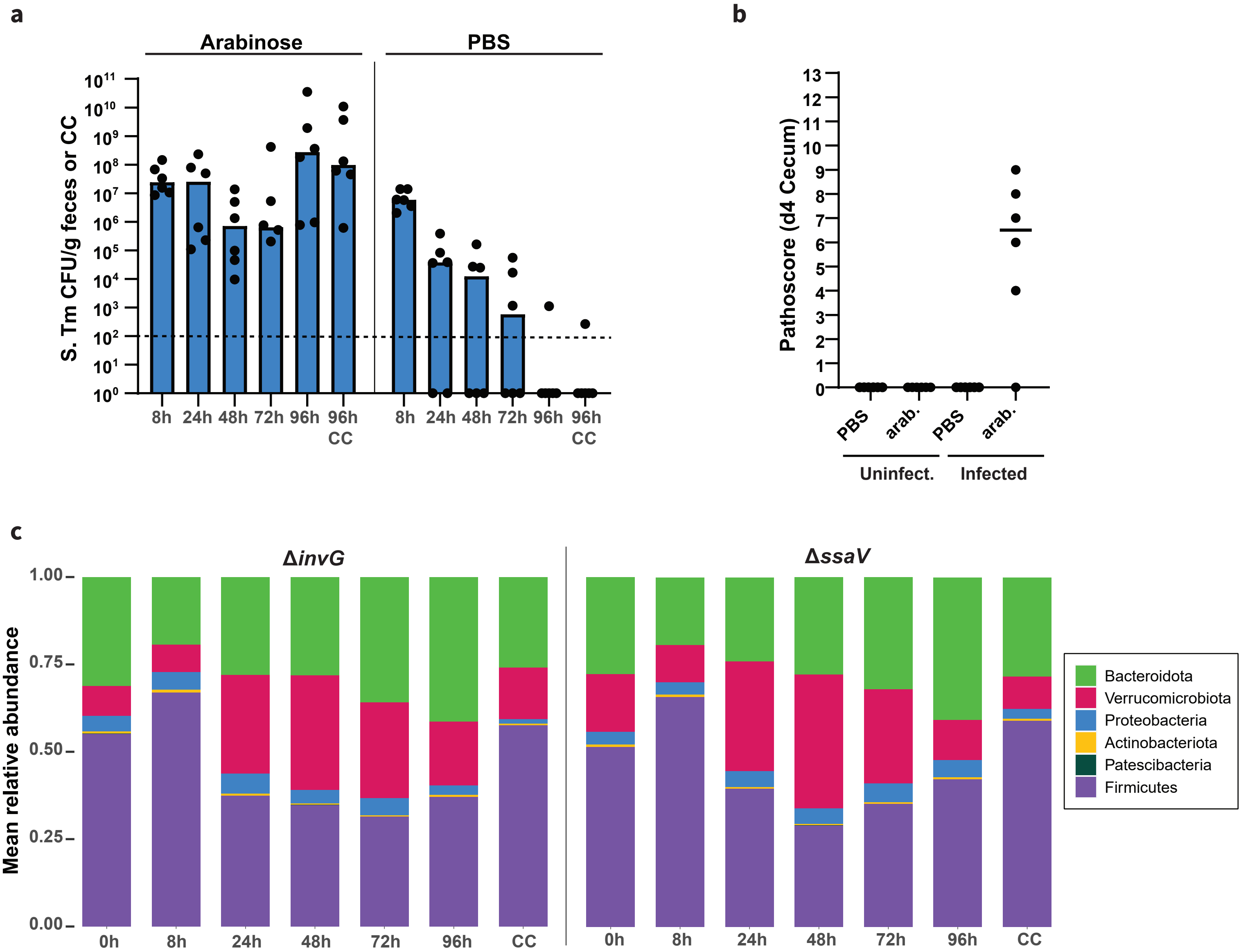

Supplementary Figure 2. Additional assessment of pathogen colonization, inflammation, and microbiota dynamics following arabinose overconsumption. Related to Figure 2.
(a) Pathogen fecal and cecal content (CC) loads enumerated by differential plating on MacConkey agar and (b) corresponding histopathological analysis from the experiment shown in Figure 2A (n = 6 mice per group). (c) 16S rRNA gene analysis of fecal microbiota composition in mice administered two doses of 132 mg arabinose and infected by oral gavage with either a Δ*invG* or Δ*ssaV* mutant. Shown is the mean composition (n = 7 mice per group).

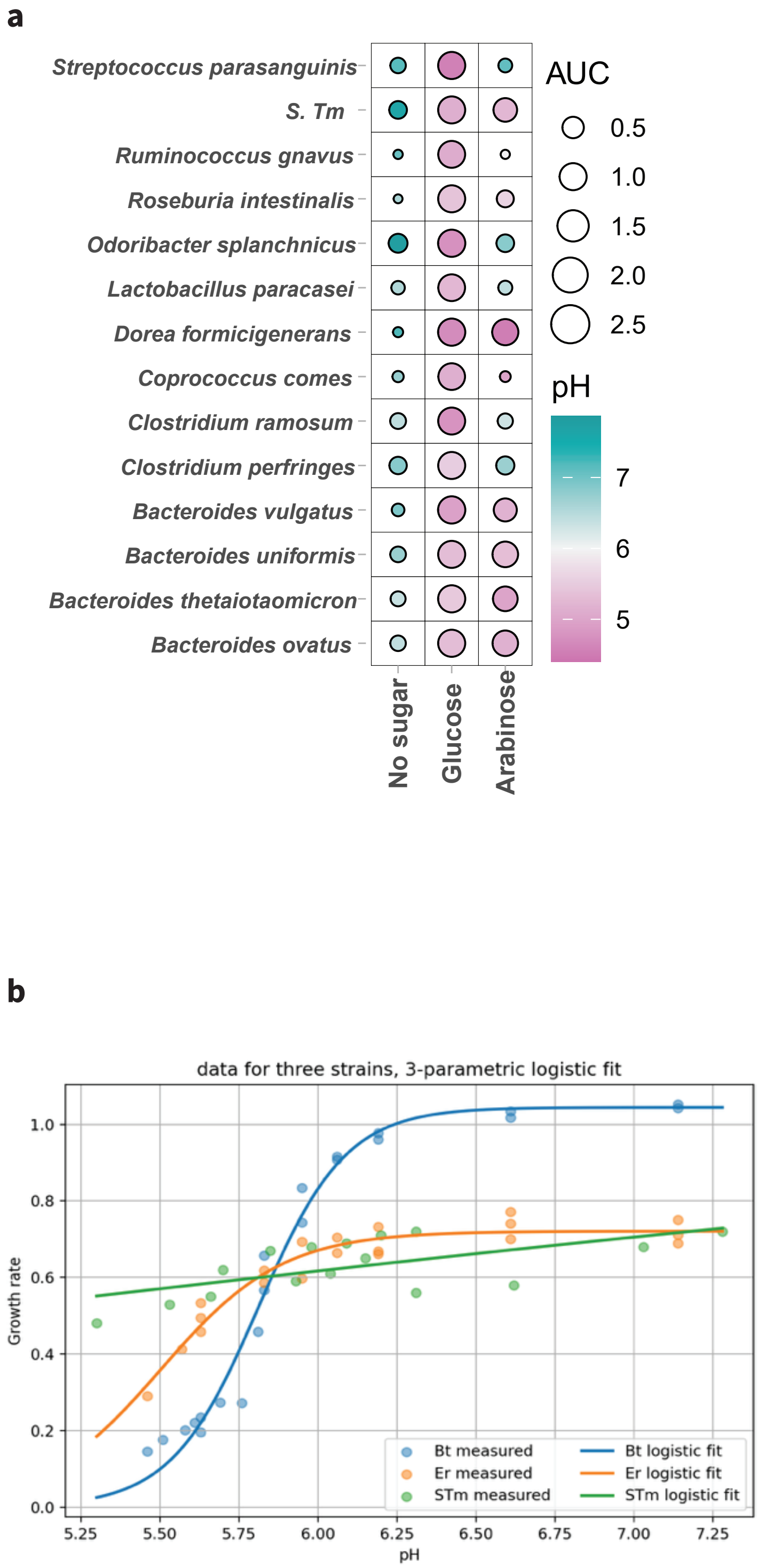

Supplementary Figure 3. **Comparative *in vitro* growth assays of microbiota strains and *S*. Tm.** Related to Figure 3. (a) *In vitro* growth assays of selected bacterial strains from a human microbiota consortium, cultured in YCSF medium supplemented with either 0.5 % glucose or 0.5 % arabinose as the primary carbon source. Growth was assessed by area under the curve (AUC). Circle radius corresponds to growth (AUC) and color indicates the final pH of the medium. (b) Comparison of growth rates of *B. thetaiotaomicron*, *E. rectale*, and *Salmonella* across pH values. Points are measured data (*B. thetaiotaomicron* and *E. rectale* data from (Cremer et al., 2017)), and lines are 3-parameteric logistic fits to the data.

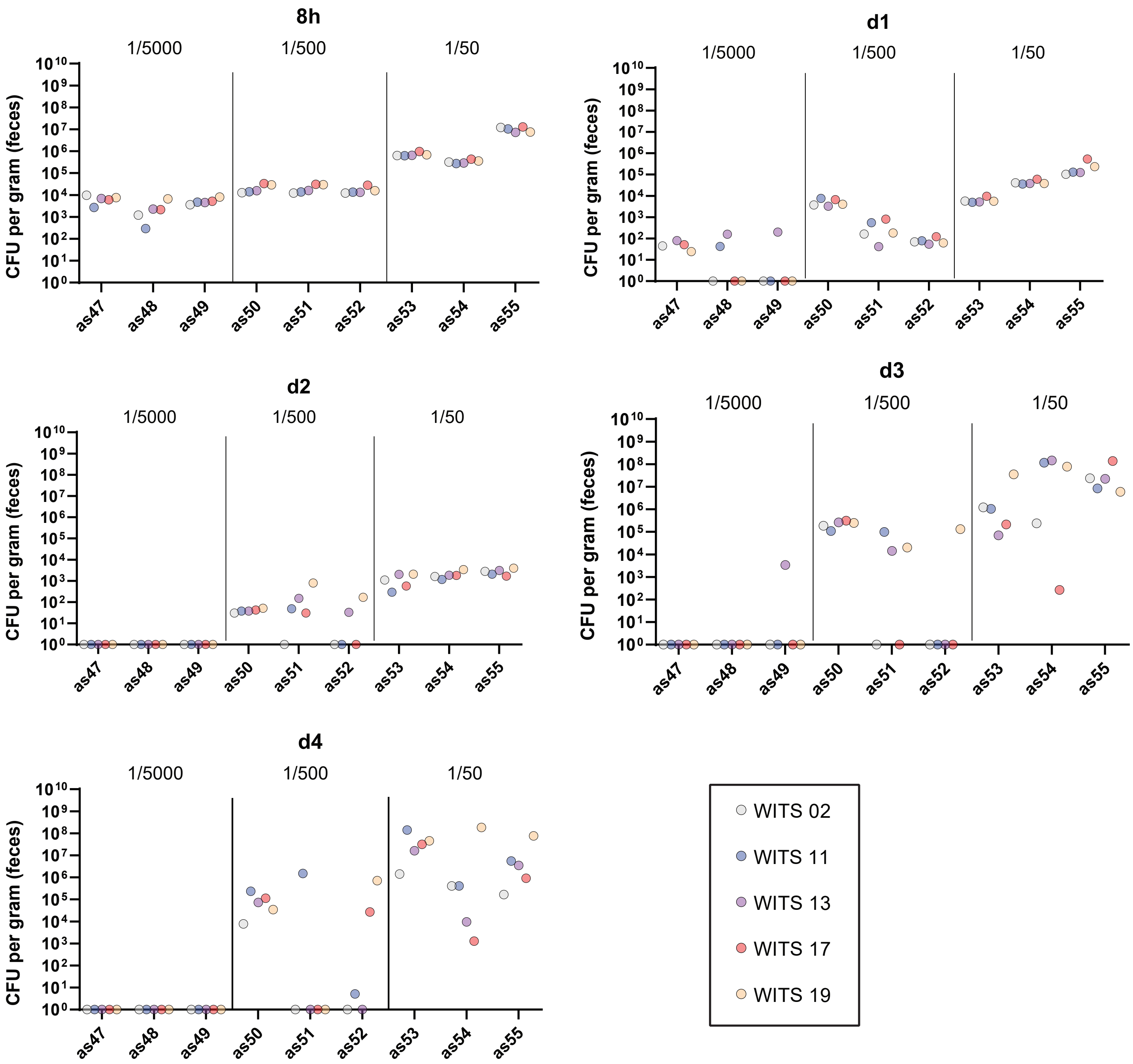

Supplementary Figure 4. Population dynamics analysis using wildtype isogenic tagged strains (WITS). Related to Figure 6. Mice were gavaged with two doses of 132 mg arabinose and infected with a mixture of tagged and untagged strains at the indicated ratios, totaling 5×10^7 CFU. WITS population sizes from feces collected 1-4 days post-infection (d1-d4) were calculated from relative abundances determined by qPCR and antibiotic plating. n=3 mice per WITS dilution with mouse identifiers (as47-as49) represented on the x-axis.

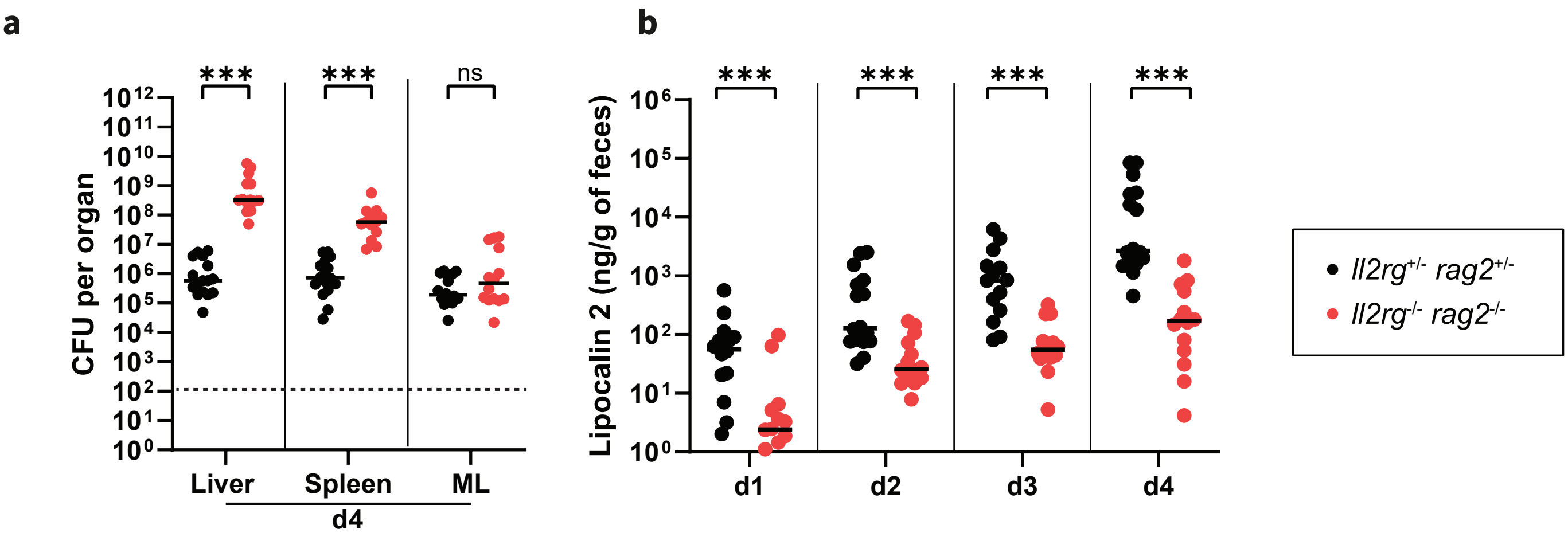

Supplementary Figure 5. **Systemic pathogen loads and intestinal inflammation in *Il2rg/Rag2* mice.** Related to Figure 7. Heterozygous and homozygous *Il2rg*/*Rag2* mice were given two doses of 132 mg arabinose and orally infected with 5 × 10^7 CFU of *S*. Tm. (a) *S*. Tm counts from systemic organs. (b) Fecal lipocalin-2 levels. The Mann–Whitney U test was used for statistical analysis; *p < 0.05; **p < 0.01; ***p < 0.001, ns, not significant.

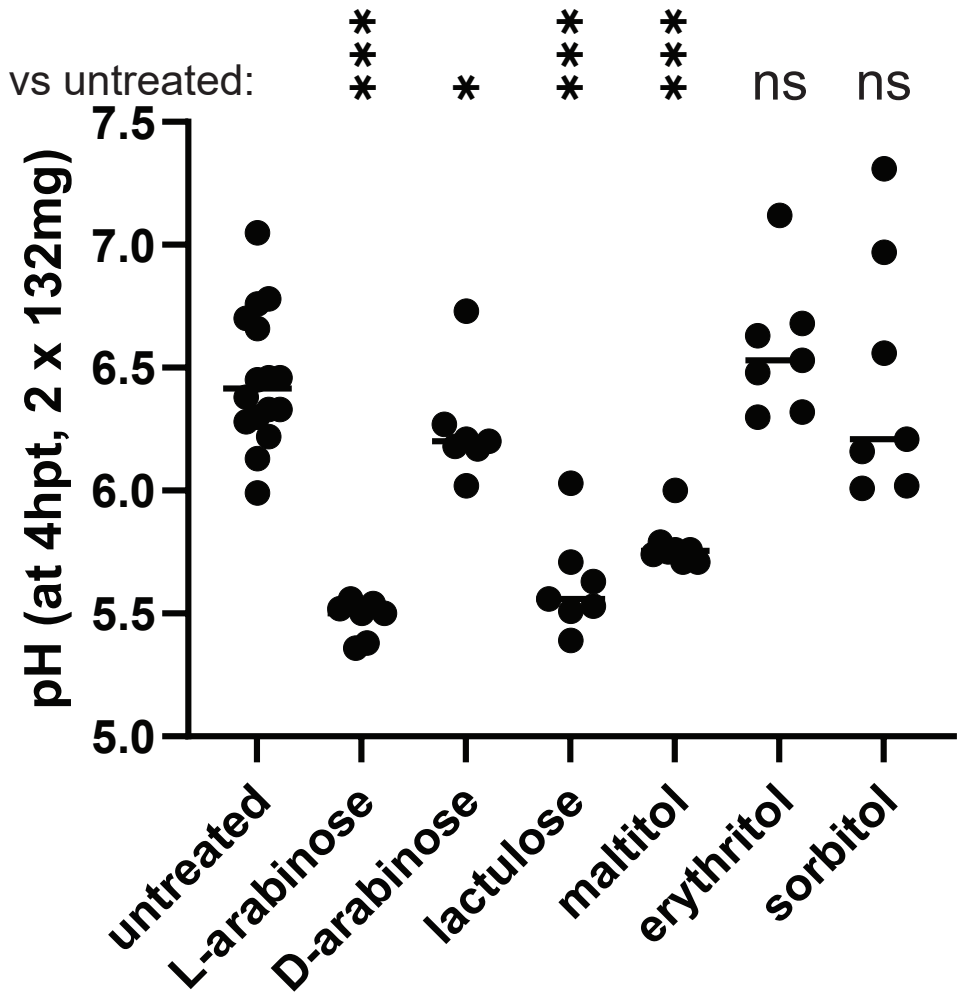

Supplementary Figure 6. **Cecal pH in mice following LDS ingestion.** Related to Figure 3. pH of cecal contents measured at 4 hours after two 132 mg doses of the indicated LDS. The Mann-Whitney U test was used for statistical analysis; *p < 0.05; **p < 0.01; *** p < 0.001; ns, not significant.

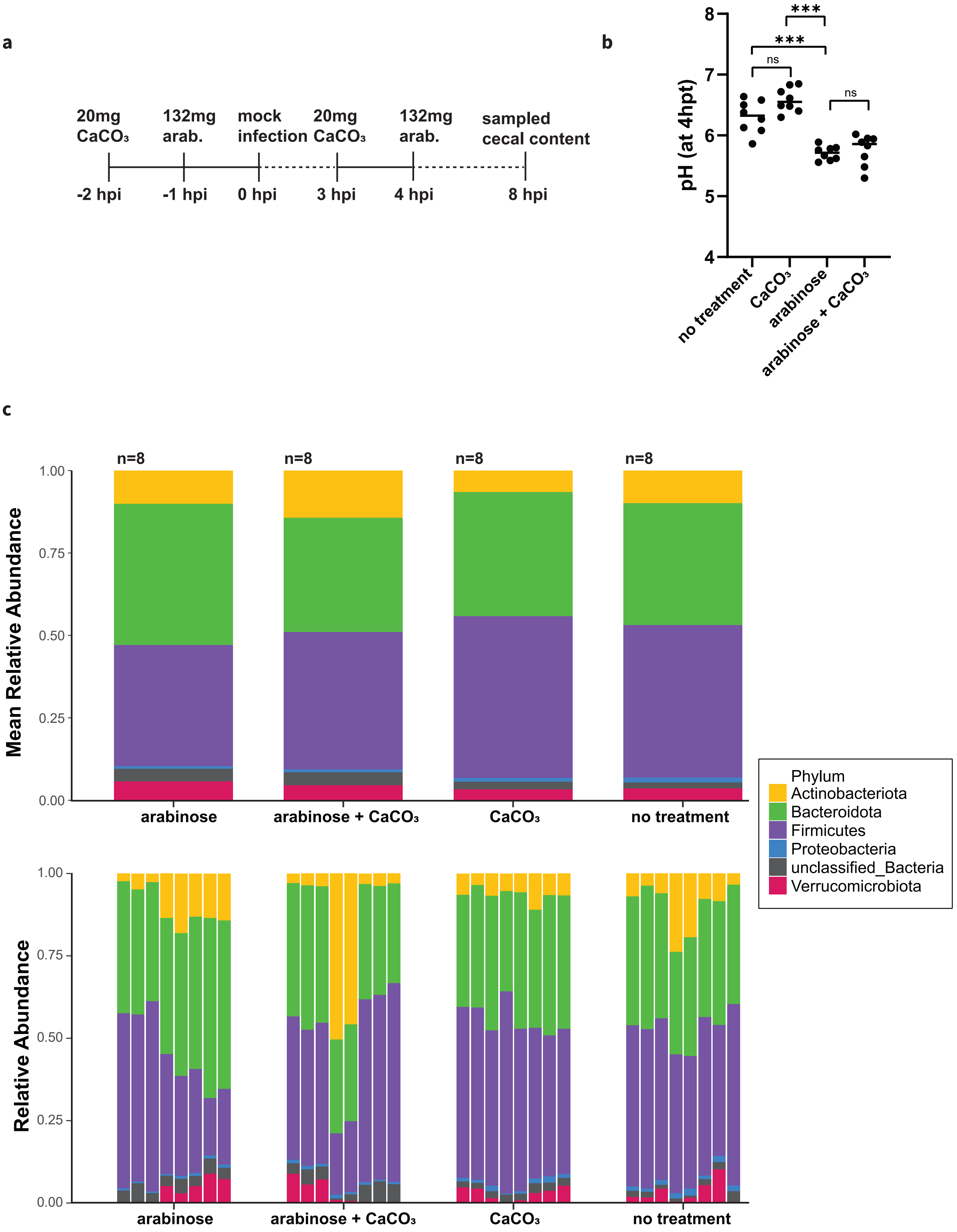

Supplementary Figure 7. See the following page for the figure caption.

d

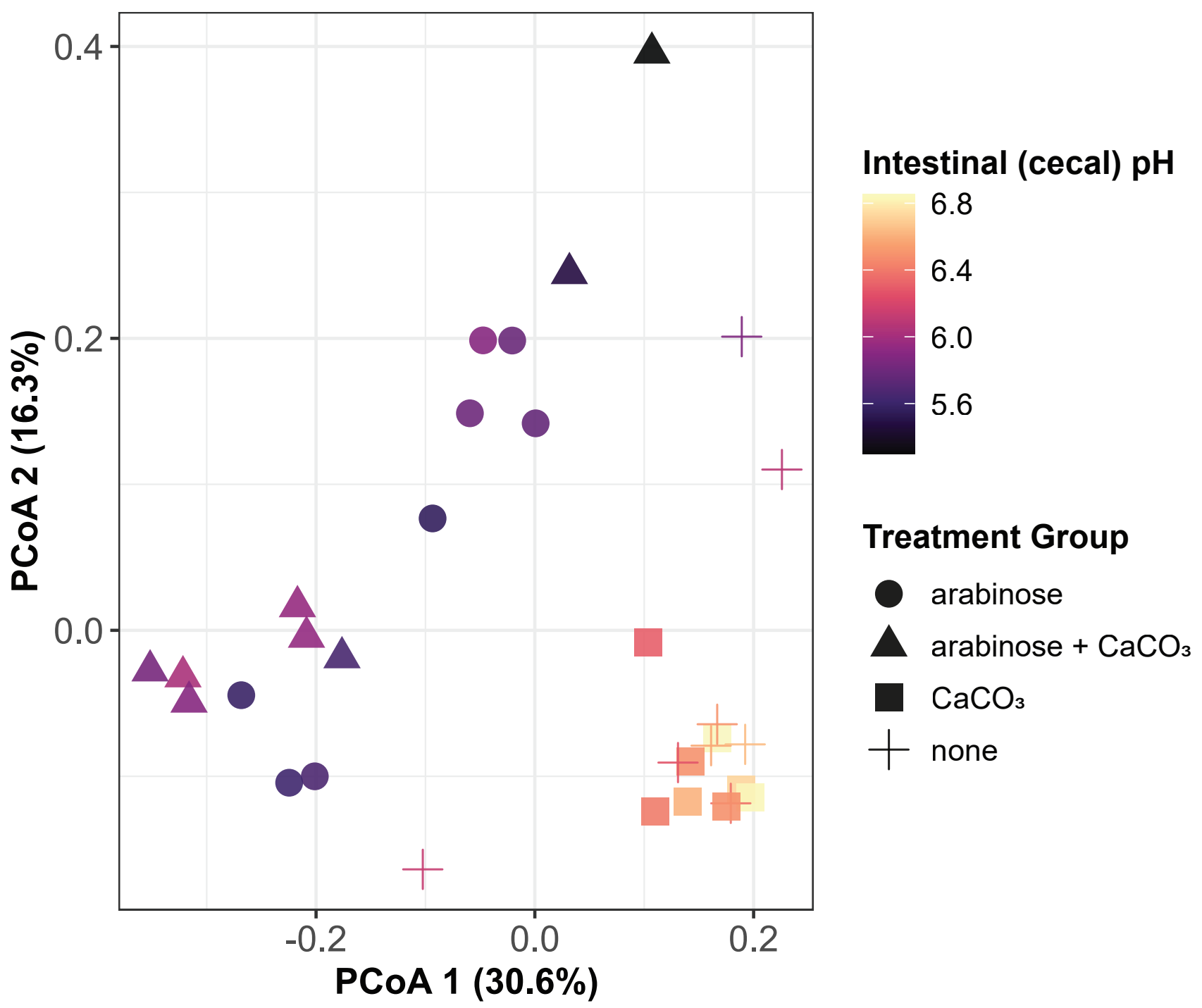

Supplementary Figure 7. Effects of calcium carbonate treatment on arabinose-associated changes in the cecal microbiota. (a) Experimental scheme. Timing of treatments corresponded to the mock infection schedule. Cecal contents were collected 4 hours after the final treatment. (b) pH measurements of cecal contents from the indicated treatment groups. (c) Cecal microbiota composition determined by 16S rRNA gene sequencing. Upper panel: mean relative abundance across mice per group; lower panel: relative abundance in individual mice. (d) Principal component analysis (PCA) of 16S rRNA gene sequencing data from the indicated groups.
