# Supplementary material for: Overconsumption of low-digestible sugars can increase susceptibility to Salmonella Typhimurium infection
Source: Nat Commun. 2026 Nov 3;17:11372. doi: 10.1038/s41467-026-78218-0 (PMC13731649; doi:10.1038/s41467-026-78218-0)
Supplement: Supplementary file 2 [file 41467_2026_78218_MOESM3_ESM.pdf]

nature portfolio

Corresponding author(s): Wolf-Dietrich Hardt and Bidong D Nguyen

Last updated by author(s): Jul 28, 2026

Reporting Summary

Nature Portfolio wishes to improve the reproducibility of the work that we publish. This form provides structure for consistency and transparency in reporting. For further information on Nature Portfolio policies, see our Editorial Policies and the Editorial Policy Checklist.

Statistics

For all statistical analyses, confirm that the following items are present in the figure legend, table legend, main text, or Methods section.

n/a	Confirmed	
☐	☒	The exact sample size (*n*) for each experimental group/condition, given as a discrete number and unit of measurement
☐	☒	A statement on whether measurements were taken from distinct samples or whether the same sample was measured repeatedly
☐	☒	The statistical test(s) used AND whether they are one- or two-sided<br>*Only common tests should be described solely by name; describe more complex techniques in the Methods section.*
☒	☐	A description of all covariates tested
☒	☐	A description of any assumptions or corrections, such as tests of normality and adjustment for multiple comparisons
☐	☒	A full description of the statistical parameters including central tendency (e.g. means) or other basic estimates (e.g. regression coefficient) AND variation (e.g. standard deviation) or associated estimates of uncertainty (e.g. confidence intervals)
☐	☒	For null hypothesis testing, the test statistic (e.g. *F*, *t*, *r*) with confidence intervals, effect sizes, degrees of freedom and *P* value noted<br>*Give P values as exact values whenever suitable.*
☒	☐	For Bayesian analysis, information on the choice of priors and Markov chain Monte Carlo settings
☒	☐	For hierarchical and complex designs, identification of the appropriate level for tests and full reporting of outcomes
☒	☐	Estimates of effect sizes (e.g. Cohen's *d*, Pearson's *r*), indicating how they were calculated

Our web collection on statistics for biologists contains articles on many of the points above.

Software and code

Policy information about availability of computer code

Data collection	Data was collected as described in the manuscript.
Data analysis	All data analysis software used in this study is described in the Materials and Methods section. For mouse experiments, statistical testing was performed using GraphPad Prism 10 for Windows. All data plots were prepared using GraphPad Prism 10.<br><br>16S rRNA Analysis: Reads were trimmed using cutadapt and analysed with the dada2 pipeline. ASVs were taxonomically annotated using IDTAXA in combination with the Silva v138 database (https://www.arb-silva.de/).<br><br>Flow Cytometry Data Analysis: Data was analysed using FlowJo 10

For manuscripts utilizing custom algorithms or software that are central to the research but not yet described in published literature, software must be made available to editors and reviewers. We strongly encourage code deposition in a community repository (e.g. GitHub). See the Nature Portfolio guidelines for submitting code & software for further information.

Data

Policy information about availability of data

All manuscripts must include a data availability statement. This statement should provide the following information, where applicable:

- Accession codes, unique identifiers, or web links for publicly available datasets
- A description of any restrictions on data availability
- For clinical datasets or third party data, please ensure that the statement adheres to our policy

In the Data Availability section, we provided the following statement: "The raw sequencing data generated in this study have been deposited in the European Nucleotide Archive (ENA) under accession numbers PRJEB115268 and ERP195487. Source data are provided with this paper (Source Data File)."

Research involving human participants, their data, or biological material

Policy information about studies with human participants or human data. See also policy information about sex, gender (identity/presentation), and sexual orientation and race, ethnicity and racism.

Reporting on sex and gender	N/A
Reporting on race, ethnicity, or other socially relevant groupings	N/A
Population characteristics	N/A
Recruitment	N/A
Ethics oversight	N/A

Note that full information on the approval of the study protocol must also be provided in the manuscript.

Field-specific reporting

Please select the one below that is the best fit for your research. If you are not sure, read the appropriate sections before making your selection.

☒ Life sciences ☐ Behavioural & social sciences ☐ Ecological, evolutionary & environmental sciences

For a reference copy of the document with all sections, see nature.com/documents/nr-reporting-summary-flat.pdf

Life sciences study design

All studies must disclose on these points even when the disclosure is negative.

Sample size	At least 5 mice were used in each group. Sample size was chosen according to institutional directives and in accordance with the 3Rs rules (Replacement, Reduction and Refinement) guiding principles underpinning the humane use of animals in research, but no statistical analyses were performed to predetermine the sample sizes.
Data exclusions	No data were excluded from the analysis
Replication	All the experiments were performed at least twice, with all attempts at data replication being successful.
Randomization	Mice, regardless of gender, were randomly allocated to different treatment groups.
Blinding	Blinding does not apply to this study because the investigators needed to identify the cages of mice for subsequent treatments/infections with respective bacterial strains.

Reporting for specific materials, systems and methods

We require information from authors about some types of materials, experimental systems and methods used in many studies. Here, indicate whether each material, system or method listed is relevant to your study. If you are not sure if a list item applies to your research, read the appropriate section before selecting a response.

Materials & experimental systems

n/a	Involved in the study
☐	☒ Antibodies
☐	☐ Eukaryotic cell lines
☐	☐ Palaeontology and archaeology
☐	☒ Animals and other organisms
☐	☐ Clinical data
☐	☐ Dual use research of concern
☐	☐ Plants

Methods

n/a	Involved in the study
☐	☐ ChIP-seq
☐	☒ Flow cytometry
☐	☐ MRI-based neuroimaging

Antibodies

Antibodies used

For bacterial flow cytometry: human anti-O12 antibody (hSTA5, gift from A. Lanzavecchia, IRB Bellinzona)kind gift of Antonio Lanzavecchia, Institute for Research in Biomedicine, Bellinzona, Switzerland Dilution 1:20

CD45 (30-F11, BioLegend), Dilution 1/100
CD4 (RM4-5, Thermo Fisher), 1/1000
Foxp3 (FJK-16s, Thermo Fisher), 1/1000
F4/80 (BM8, BioLegend), Dilution 1/400
CD103 (2E7, BioLegend), Dilution 1/100
MHCII (M5/114.15.2, BioLegend), Dilution 1/400
CD11b (M1/70, BioLegend), Dilution 1/200
CD11c (N418, BioLegend), Dilution 1/200
Sytox Blue (Invitrogen), Dilution 1/1000
Zombie NIR (BioLegend), Dilution 1/1000

Validation

The human anti-S. Typhimurium O:12 antibody (hSTA5) used for bacterial flow cytometry was previously validated (Kroon et al., 2025 doi: 10.1038/s41467-025-57979-0)

The antibodies were validated by the manufacturer:
CD45 https://www.biolegend.com/en-gb/products/percp-anti-mouse-cd45-antibody-4265
CD4 https://www.thermofisher.com/antibody/product/CD4-Antibody-clone-RM4-5-Monoclonal/14-0042-82
Foxp3 https://www.thermofisher.com/antibody/product/FOXP3-Antibody-clone-FJK-16s-Monoclonal/12-5773-82
F4/80 https://www.biolegend.com/en-gb/products/apc-anti-mouse-f4-80-antibody-4071?GroupID=BLG5319
MHCII https://www.biolegend.com/en-us/products/brilliant-violet-421-anti-mouse-i-a-i-e-antibody-7147
CD11b https://www.biolegend.com/de-at/products/purified-anti-mouse-human-cd11b-antibody-351
CD11c https://www.biolegend.com/en-us/products/pe-cyanine7-anti-mouse-cd11c-antibody-3086
Sytox Blue https://www.thermofisher.com/order/catalog/product/S34857

Eukaryotic cell lines

Policy information about cell lines and Sex and Gender in Research

Cell line source(s): N/A

Authentication: N/A

Mycoplasma contamination: N/A

Commonly misidentified lines (See ICLAC register): N/A

Palaeontology and Archaeology

Specimen provenance: N/A

Specimen deposition: N/A

Dating methods: N/A

☐ Tick this box to confirm that the raw and calibrated dates are available in the paper or in Supplementary Information.

Ethics oversight: N/A

Note that full information on the approval of the study protocol must also be provided in the manuscript.

Animals and other research organisms

Policy information about studies involving animals; ARRIVE guidelines recommended for reporting animal research, and Sex and Gender in Research

Laboratory animals	7- to 12-week old C57BL/6J (The Jackson Laboratory) mice were held under specific pathogen-free (SPF) conditions at the EPIC facility at ETH Zürich (light/dark cycle 12:12 h, room temperature 21±1 °C, humidity 50±10%).
Wild animals	No wild animals were used
Reporting on sex	7-12 week old mice of both sexes were randomly assigned to experimental groups. The consideration of animal sex was not included in the study design.
Field-collected samples	This study did not involve samples collected from the field.
Ethics oversight	All studies were conducted in accordance with ethical and legal requirements and were reviewed and approved by the Kantonales Veterinäramt Zürich under the licenses: ZH193/2016, ZH158/19, and ZH109/22.

Note that full information on the approval of the study protocol must also be provided in the manuscript.

Clinical data

Policy information about clinical studies

All manuscripts should comply with the ICMJE guidelines for publication of clinical research and a completed CONSORT checklist must be included with all submissions.

Clinical trial registration	N/A
Study protocol	N/A
Data collection	N/A
Outcomes	N/A

Dual use research of concern

Policy information about dual use research of concern

Hazards

Could the accidental, deliberate or reckless misuse of agents or technologies generated in the work, or the application of information presented in the manuscript, pose a threat to:

No	Yes	
☒	☐	Public health
☒	☐	National security
☒	☐	Crops and/or livestock
☒	☐	Ecosystems
☒	☐	Any other significant area

Experiments of concern

Does the work involve any of these experiments of concern:

No	Yes	
☒	☐	Demonstrate how to render a vaccine ineffective
☒	☐	Confer resistance to therapeutically useful antibiotics or antiviral agents
☒	☐	Enhance the virulence of a pathogen or render a nonpathogen virulent
☒	☐	Increase transmissibility of a pathogen
☒	☐	Alter the host range of a pathogen
☒	☐	Enable evasion of diagnostic/detection modalities
☒	☐	Enable the weaponization of a biological agent or toxin
☒	☐	Any other potentially harmful combination of experiments and agents

Plants

Seed stocks	N/A
Novel plant genotypes	N/A
Authentication	N/A

ChIP-seq

Data deposition

☐ Confirm that both raw and final processed data have been deposited in a public database such as GEO.

☐ Confirm that you have deposited or provided access to graph files (e.g. BED files) for the called peaks.

Data access links *May remain private before publication.*	N/A
Files in database submission	N/A
Genome browser session (e.g. UCSC)	N/A

Methodology

Replicates	N/A
Sequencing depth	N/A
Antibodies	N/A
Peak calling parameters	N/A
Data quality	N/A
Software	N/A

Flow Cytometry

Plots

Confirm that:

☒ The axis labels state the marker and fluorochrome used (e.g. CD4-FITC).

☒ The axis scales are clearly visible. Include numbers along axes only for bottom left plot of group (a 'group' is an analysis of identical markers).

☒ All plots are contour plots with outliers or pseudocolor plots.

☒ A numerical value for number of cells or percentage (with statistics) is provided.

Methodology

Sample preparation

Analysis of sicA promoter activity (adapted from Kroon et al., 2025; doi: 10.1038/s41467-025-57979-0) . Analysis of sicA promoter activityActivity of the sicA promoter was analysed in mice using a plasmid-based P_sicA_-gfp reporter. Fresh caecal contents were collected at 24 h post-infection and diluted in PBS. The samples were incubated for 1 h at room temperature with 2 µg ml⁻¹ chloramphenicol (AppliChem) to inhibit protein synthesis and allow existing GFP molecules to mature fully. S. Tm was stained using the human anti-S. Tm O:12 antibody hSTA5, kindly provided by Antonio Lanzavecchia (Institute for Research in Biomedicine, Bellinzona, Switzerland), followed by a goat anti-human IgG Alexa Fluor 647 antibody (Jackson ImmunoResearch Europe). Fluorescence was measured using a CytoFLEX flow cytometer (Beckman Coulter), and data were acquired using CytExpert software v2.5.

Cecum lamina propria cell isolation (adapted from Wotzka et al., 2019; doi:10.1038/s41564-019-0568-5)..Ceca were excised, opened longitudinally, cut into small pieces, and incubated twice in 14 mL PBS containing 5 mM EDTA, 15 mM HEPES, and

	10% FCS (20 min, 37 °C, mild shaking). After washing in RPMI containing 30% FCS, tissues were digested in RPMI containing 1 mg/mL collagenase VIII and 0.2 mg/mL DNase I (1 h, 37 °C, mild shaking). Samples were filtered through a 70 µm strainer and separated using a NycoPrep 1.077 gradient (30 min, 400 × g). Interphase cells were collected, washed in RPMI containing 15% FCS, and resuspended in PBS containing 1% FCS. Following surface staining, cells were fixed and permeabilized with Foxp3 Fix/Perm buffer (40 min, room temperature, protected from light), washed, and stained for intracellular markers under the same conditions.
Instrument	LSR Fortessa (BD Biosciences), Beckman Coulter CytoflexS
Software	DIVA, FlowJo V10, CytExpert software v.2.5
Cell population abundance	For lamina propria cell isolation, approximately 10% of acquired events were live CD45⁺ cells.<br>For S. Tm GFP analysis, approximately 6% of acquired events were positive for S. Tm O:12 staining.
Gating strategy	Bacterial flow cytometry. The bacterial flow-cytometry gating strategy was adapted from Kroon et al. (2025). S. Tm P_sicA_-gfp bacteria were identified using a human anti-S. Tm O:12 primary antibody followed by a goat anti-human Alexa Fluor 647 secondary antibody. Controls included a fluorescence-minus-one control in which the primary antibody was omitted and an S. Tm control strain that did not express GFP.<br><br>Lamina propria cells.The initial gate was defined according to cell size and granularity. All subsequent gates were set using pseudocolor-plot cut-offs. The gating strategy was based on a previous publication by Wotzka et al. (2019).Identifying intestinal regulatory T cells: live leukocytes were identified based on CD45 expression and Zombie-NIR exclusion. T cells were subsequently identified using CD4, which were further gated for FOXP3+ cells to identify regulatory T cells. Identification of intestinal dendritic-cell subsets and macrophages: the staining panel used to distinguish intestinal DC populations and macrophages included CD45, CD11c, MHC II, F4/80, CD11b, and CD103. CD45 and SYTOX Blue were used to identify viable leukocytes. MHC II-positive antigen-presenting cells were subsequently divided based on expression of the macrophage marker F4/80 and the DC marker CD11c. The F4/80⁻CD11c⁺ population was then separated into four DC subsets according to CD103 and CD11b expression. Example Gating strategies are found in the Source Data file.

☒ Tick this box to confirm that a figure exemplifying the gating strategy is provided in the Supplementary Information.

Magnetic resonance imaging

Experimental design

Design type	N/A
Design specifications	N/A
Behavioral performance measures	N/A

Acquisition

Imaging type(s)	N/A
Field strength	N/A
Sequence & imaging parameters	N/A
Area of acquisition	N/A

Diffusion MRI ☐ Used ☐ Not used

Preprocessing

Preprocessing software	N/A
Normalization	N/A
Normalization template	N/A
Noise and artifact removal	N/A
Volume censoring	N/A

Statistical modeling & inference

Model type and settings	N/A

Effect(s) tested: N/A

Specify type of analysis: ☐ Whole brain ☐ ROI-based ☐ Both

Statistic type for inference (See Eklund et al. 2016): *Specify voxel-wise or cluster-wise and report all relevant parameters for cluster-wise methods.*

Correction: N/A

Models & analysis

n/a	Involved in the study
☒	☐ Functional and/or effective connectivity
☒	☐ Graph analysis
☒	☐ Multivariate modeling or predictive analysis
