# Supplementary material for: Overconsumption of low-digestible sugars can increase susceptibility to Salmonella Typhimurium infection
Source: Nat Commun. 2026 Nov 3;17:11372. doi: 10.1038/s41467-026-78218-0 (PMC13731649; doi:10.1038/s41467-026-78218-0)
Supplement: Supplementary file 3 [file 41467_2026_78218_MOESM4_ESM.pdf]

nature portfolio

Peer Review File

Overconsumption of low-digestible sugars can increase susceptibility to *Salmonella* Typhimurium infection

Corresponding Author: Professor Wolf-Dietrich Hardt

This manuscript has been previously reviewed at another journal that is not operating a transparent peer review scheme. The manuscript was considered suitable for publication without further review at Nature Communications.

Version 0:

Reviewer comments:

Reviewer #1

(Remarks to the Author)
This manuscript identifies that low-digestible carbohydrates (LDCs) enhance Salmonella infection. By focusing on the LDC arabinose, they provide mechanistic insights on how LDCs promote pathogen colonization. Specifically, they show that arabinose overconsumption results in gut acidification and they propose that this low pH environment shifts the microbiota composition, thus reducing colonization resistance and driving Salmonella expansion. They further found that arabinose consumption promotes Salmonella virulence by increasing SPI-1 expression, broadening tissue tropism, and enhancing systemic infection. This work addresses an important gap in our understanding how dietary sugars affect intestinal pathogen virulence and will be of broad interest to those studying the effects of diet on infection, host-pathogen interactions, and microbial metabolism. However, a few aspects require further clarification and experimental strengthening to firmly establish the mechanistic claims.
Major points
1. Strengthening claim that low pH reduces Bacteroidota species. In the results section for Fig 3, the authors show that arabinose leads to decreases in the gut pH and speculate that this may be why pH-sensitive Bacteroidota species important for S.Tm. colonization resistance decrease after arabinose administration. To strengthen the claim that the reduction in pH leads to a decrease in Bacteroidota, it is recommended that the authors repeat their calcium carbonate experiments where the pH is restored and determine whether in these conditions there is no longer a reduction in Bacteroidota species.

2. Determining whether amino acids promote S.Tm blooms. In Fig 4, the authors observed an increase in several amino acids following arabinose administration. They state that these amino acids may help Salmonella bloom, but no direct evidence was provided. Suggested experiment: Infect mice that have been administered arabinose with amino acid auxotrophic strains of Salmonella to determine whether any of the increased amino acids help Salmonella bloom after arabinose administration.

3. The data presented in Figure 2 B and C include only 5 mice, and appears to have been performed one time. Given the spread in the data points, the authors should perform the experiments again with more mice and have repeated at least 3 times.
Minor points:
1. Fig S1A says “A. Histopathological analysis corresponding to Figure 1E”. This seems to be a typo since S1A corresponds to Figure 1F.

2. Lines 194-195: “by day 2 p.i. Inflammation then drives drastic changes in the microbiome, leading to a robust Tm bloom on days 3–4 p.i.” Since the authors did not directly prove that inflammation drives changes in the microbiome, the language in this paragraph should be modified to be more speculative.

3. Line 299: typo in “as efficiently at wild-type Tm”. The “at” should be “as”

4. Lines 356-357: reference for “Based on previous work in antibiotic pretreated mice, such re-seeding events should be comparatively rare”

5. Lines 411-413: The following sentence “While these compositional changes were subtle, they sufficiently promoted

pathogen growth through increased availability of nutrients by an impaired microbiota, including amino acids that supply carbon and nitrogen." Seems like an overinterpretation and should be written as more speculative.

6. Supplementary Figure 6: pH results for sorbitol, erythritol, and maltitol are mentioned in the discussion section (Lines 438-445). If the data is available, it should be included in this figure.

Reviewer #2

(Remarks to the Author)
Results: The Authors investigate that certain LDCs, particularly low digestible sugars (LDSs) such as arabinose, lactulose, and polyols commonly used as sugar substitutes, can impair colonization resistance against Salmonella enterica serovar Typhimurium.
They show that acute overconsumption of LDSs results in elevated fecal pathogen loads, systemic dissemination, and colitis in infected mice.

Comment1: The findings are technically well done and convincingly shown.
But does not acute overconsumption of any food lead to elevated fecal loads and microbiome shifts?
So please discuss more such clear limitations of your study.

Results II: By focussing on arabinose, the authors observed that rapid fermentation of this sugar acidifies the intestinal environment, which alters the microbiota composition and metabolite profile,including shifts in short-chain fatty acids and elevated amino acids that fuel pathogen growth. Arabinose consumption impacts virulence by broadening tissue tropism, enhancing systemic infection, and selecting for fully virulent, invasive Salmonella through modulation of microbiota–pathogen interactions.

Comment2: Again the question regarding specificity arises: so how far are these effects only the effect of an acidification of the intestinal environment, so have not really to do with LDS?
Please show some convincing data on the specificity of the effect, compare as control with another acidifying food.

Abstract III: These findings
reveal how diet can impact the gut microbiota in ways that enhance pathogen colonization and virulence.
Comment 3: Please modify in the light of the revision comments, in the moment one should rather state:
"These data show how a strong food stimulus including strong acidification changes the gut intestine. Further studies are necessary to be more specific, for instance to reveal the specific effects of a LDS pulse."
The infection data are quite nice, including SPI-1 and SPI-2 involvement and data, but again, they only show how an infection comes about, nothing specific yet (or if I overlooked here something, then stress these specific points, will be interesting to read).

Discussion:
--You start the discussion with two very informative reviews: Kreuzer and Hardt, 2020, Woelfel et al., 2024. These also compare what is known for gut microbiota and colonization resistance not only in the mouse model but also what applies to the human and patients.
So please expand this section and convince the reader that you really can add here compared to what is known from the two reviews, particular regarding human resistance to Salmonella infection.
Again, it is completely fine if you want in the end just to say "These data show how a strong food stimulus including strong acidification changes the gut intestine in our mouse model. Further studies are necessary to be more specific, for instance to reveal the specific effects of a LDS pulse, and to be more sure how far this can be translated to humans and ultimately, patients." However, the reader should not be left in the dark about this really important point.

--Moreover, as far as I see (but may be you know even better), at least regarding the mouse model this is a rather extensive and detailed study for such a food stimulus and the resulting infection and so you can stress this convincing positive point of your study, too, in the discussion.

Minor:
Overall statistics is well done, but only in Figure 2 we learn N = 5 mice per group, please tell the group sizes also for the other figures were appropriate

Version 1:

Reviewer comments:

Reviewer #1

(Remarks to the Author)

Major point #1 It is concerning that the authors cannot reproduce their pH neutralization with calcium carbonate, which makes me think that their data in Fig 3G and 3H using calcium carbonate is not reproducible. If this is the case, I don't think it is appropriate nor rigorous for them to publish any data using calcium carbonate that is not reproducible.

Manor point #2 I still think their data do not directly support their conclusion regarding inflammation. In my opinion, they should use an anti-inflammatory drug and show that reducing inflammation during infection reverts the microbiota composition to those of uninfected arabinose-treated mice if they want to state that inflammation is driving changes in the microbiota at days 3-4. With their current data, they can only say that Salmonella virulence drives microbiota changes at days 3-4.

Reviewer #2

(Remarks to the Author)
Thank you for your well taken revisions:
1) it is essential to distinguish between the effects of general overconsumption and those driven by specific dietary components. These findings indicate that overconsumption per se is not sufficient to disrupt
colonization resistance. In response to the reviewer's comment, we have clarified this point in the Discussion
(Lines 387-392). We also now explicitly acknowledge the limitation that our study did
not constitute an exhaustive screen of dietary nutrients or food combinations, and that
additional compounds or mixed dietary contexts may reveal further mechanisms
influencing colonization resistance (Lines 488-496).
--> well done
2) how far are these effects only the effect of an acidification of the intestinal environment?
We have now included additional data in Supplemental Fig.6 showing intestinal pH
measurements for all LDSs tested in this study.
In response to the reviewer's comment, we have revised the text (Lines 378-385) to
clarify this distinction and to avoid implying that all LDSs act through one common
mechanism
--> well done
3) we now more clearly describe that arabinose
overconsumption leads to intestinal acidification, microbiota perturbation, and
metabolite changes and that these changes are associated with enhanced pathogen
growth, while pointing out that the full mechanistic details explaining the enhanced
growth await further studies and avoiding overgeneralization across all LDSs.
--> well done
4) we have expanded the opening paragraph of the Discussion (Lines 338-
352) to better frame the translational relevance of our study
--> well done
Minor:
we identified and corrected an error in the labeling of group sizes in Figure 2 and Supplementary Figure 2B; the correct group
sizes are n = 6 for Figure 2 and n = 7 for Supplementary Figure 2B.
--> fine

Nothing left, all done!

Open Access This Peer Review File is licensed under a Creative Commons Attribution 4.0 International License, which permits use, sharing, adaptation, distribution and reproduction in any medium or format, as long as you give appropriate credit to the original author(s) and the source, provide a link to the Creative Commons license, and indicate if changes were made.
In cases where reviewers are anonymous, credit should be given to 'Anonymous Referee' and the source.
The images or other third party material in this Peer Review File are included in the article's Creative Commons license, unless indicated otherwise in a credit line to the material. If material is not included in the article's Creative Commons license and your intended use is not permitted by statutory regulation or exceeds the permitted use, you will need to obtain permission directly from the copyright holder.
To view a copy of this license, visit https://creativecommons.org/licenses/by/4.0/

REVIEWER COMMENTS

Reviewer #1 (Remarks to the Author):

This manuscript identifies that low-digestible carbohydrates (LDCs) enhance Salmonella infection. By focusing on the LDC arabinose, they provide mechanistic insights on how LDCs promote pathogen colonization. Specifically, they show that arabinose overconsumption results in gut acidification and they propose that this low pH environment shifts the microbiota composition, thus reducing colonization resistance and driving Salmonella expansion. They further found that arabinose consumption promotes Salmonella virulence by increasing SPI-1 expression, broadening tissue tropism, and enhancing systemic infection. This work addresses an important gap in our understanding how dietary sugars affect intestinal pathogen virulence and will be of broad interest to those studying the effects of diet on infection, host-pathogen interactions, and microbial metabolism. However, a few aspects require further clarification and experimental strengthening to firmly establish the mechanistic claims.

Major points

1. Strengthening claim that low pH reduces Bacteroidota species. In the results section for Fig 3, the authors show that arabinose leads to decreases in the gut pH and speculate that this may be why pH-sensitive Bacteroidota species important for S.Tm. colonization resistance decrease after arabinose administration. To strengthen the claim that the reduction in pH leads to a decrease in Bacteroidota, it is recommended that the authors repeat their calcium carbonate experiments where the pH is restored and determine whether in these conditions there is no longer a reduction in Bacteroidota species.:

RESPONSE:

We thank the reviewer for this thoughtful suggestion and performed the requested experiment (attached **Figure X**). However, in these follow-up experiments, the calcium carbonate dosing regimen that had previously restored intestinal pH did not reproducibly achieve the same degree of pH neutralization, preventing a definitive assessment of whether restoration of pH rescues Bacteroidota abundance (**Figure XA, B**). Despite the incomplete neutralization, we performed 16S rRNA gene profiling of the follow-up cohort and found that the baseline microbiota composition differed substantially from that shown in **Figure 2**, including markedly reduced levels of Verrucomicrobiota and increased Actinobacteriota (**Figure XC**). This suggests that cohort-specific differences in the microbial community may have influenced the experimental outcome. One possible contributing factor is diet, as our animal facility informed us of inconsistencies between recent chow lots, and as a result, the facility is in the process of changing vendors. As diet can influence microbiota composition and

host physiology, the altered experimental conditions may also have affected the efficacy of calcium carbonate treatment.

Consistent with the failure to fully restore pH, we did not observe significant microbiota differences between arabinose-treated mice and arabinose + calcium carbonate mice, both of which maintained relatively low intestinal pH (5.3–5.9; **Figure XB, C**). Moreover, we did not observe a clear Bacteroidota trend across groups, likely due to the substantially altered baseline microbiota composition in this cohort (**Figure XC)**. PCA analysis nevertheless showed that both groups remained clearly distinct from untreated and calcium carbonate-only controls, which maintained higher intestinal pH values (5.9–6.9; **Figure XB, D**), suggesting that microbiota shifts still broadly tracked with intestinal pH.

As a result, we could not directly test the causal relationship between pH restoration and recovery of Bacteroidota abundance in the current revision, and therefore we do not consider these follow-up data sufficiently interpretable for inclusion in the revised manuscript. That said, the follow-up experiment remains broadly consistent with our overall conclusion that arabinose overconsumption induces pH-associated perturbations of the intestinal microbiota. The manuscript does not present reduction of Bacteroidota as a definitive mechanistic explanation for loss of colonization resistance, but rather as one possible interpretation (e.g., "may explain" and "suggesting a potential mechanism**," Lines 186–190)** among others.

2. Determining whether amino acids promote *S*.Tm blooms. In Fig 4, the authors observed an increase in several amino acids following arabinose administration. They state that these amino acids may help Salmonella bloom, but no direct evidence was provided. Suggested experiment: Infect mice that have been administered arabinose with amino acid auxotrophic strains of Salmonella to determine whether any of the increased amino acids help Salmonella bloom after arabinose administration.

RESPONSE:

We thank the reviewer for this helpful suggestion. We have performed the requested experiments using amino acid auxotrophic *S.*Tm strains. Specifically, auxotrophs defective in lysine, phenylalanine, or arginine biosynthesis exhibited growth comparable to, or in some cases exceeding, that of the wild-type strain in arabinose-treated mice at day 1 post-infection. These findings indicate that the intestinal environment created by arabinose overconsumption includes sufficient amino acids to relax amino acid biosynthetic requirements for *S.*Tm. This is consistent with the relatively high amino acid availability detected in our metabolomic analysis (**Figure 4B**). Thereby the new data supports the conclusion that elevated amino acid pools can contribute to *Salmonella* expansion under these conditions.

These new data have been included as **Figure 4C** and described in **Lines 224-227**

In addition, we now cite recent studies supporting this interpretation (Pickard et al., 2025; Shealy et al., 2025), which independently demonstrate that increased amino acid availability can promote *S.*Tm growth and compromise colonization resistance.

3. The data presented in Figure 2 B and C include only 5 mice, and appears to have been performed one time. Given the spread in the data points, the authors should perform the experiments again with more mice and have repeated at least 3 times.

RESPONSE:
We have repeated the experiment two additional times, resulting in a total of four independent experiments (N=10 mice group). With the increased statistical power, we still found no significant difference between the arabinose-treated and untreated groups in CFU counts in feces, cecal contents, and organs. Therefore, our conclusions remain unchanged.

Minor points:
1. Fig S1A says "A. Histopathological analysis corresponding to Figure 1E". This seems to be a typo since S1A corresponds to Figure 1F.

Corrected.

2. Lines 194-195: "by day 2 p.i. Inflammation then drives drastic changes in the microbiome, leading to a robust S. Tm bloom on days 3–4 p.i." Since the authors did not directly prove that inflammation drives changes in the microbiome, the language in this paragraph should be modified to be more speculative.

RESPONSE:
We appreciate the reviewer's comment. We have supporting evidence for this statement that was already described in the manuscript but not explicitly referenced. Histopathological analysis of the cecum at day 4 post-infection (**Supplementary Figure 2B**) shows inflammation only in arabinose-treated mice infected with wild-type *Salmonella*, which also exhibited marked microbiota changes on days 3-4 p.i. (**Figure 2A**). Moreover, mice infected with the avirulent ΔinvG or ΔssaV mutants, which do not induce inflammation, maintained microbiota compositions resembling those of uninfected arabinose-treated mice and did not undergo the pronounced microbiota shifts or pathogen expansion observed with wild-type infection (**Supplementary Figure 2C**). Together, these data support the conclusion that pathogen-induced inflammation drives the more drastic microbiota changes observed during the later phase of infection.

To clarify this point, we have revised the text and added the relevant figure references in **LINES 159-162**.

3. Line 299: typo in "as efficiently at wild-type S. Tm". The "at" should be "as"

Corrected.

4. Lines 356-357: reference for "Based on previous work in antibiotic pretreated mice, such re-seeding events should be comparatively rare"

We added the reference, Bakkeren, E., et al 2019 in **LINES 298-299**.

5. Lines 411-413: The following sentence "While these compositional changes were subtle, they sufficiently promoted pathogen growth through increased availability of nutrients by an impaired microbiota, including amino acids that supply carbon and nitrogen." Seems like an overinterpretation and should be written as more speculative.

RESPONSE:
As addressed in our response to major point #2, we now provide functional support for this interpretation.

6. Supplementary Figure 6: pH results for sorbitol, erythritol, and maltitol are mentioned in the discussion section (Lines 438-445). If the data is available, it should be included in this figure.

RESPONSE:
We have now included the pH data for sorbitol, erythritol, and maltitol in **Supplementary Figure 6**.

Reviewer #2 (Remarks to the Author):

Results: The Authors investigate that certain LDCs, particularly low digestible sugars (LDSs) such as arabinose, lactulose, and polyols commonly used as sugar substitutes, can impair colonization resistance against Salmonella enterica serovar Typhimurium. They show that acute overconsumption of LDSs results in elevated fecal pathogen loads, systemic dissemination, and colitis in infected mice.

Comment1: The findings are technically well done and convincingly shown. But does not acute overconsumption of any food lead to elevated fecal loads and microbiome shifts? So please discuss more such clear limitations of your study.

RESPONSE:
We thank the reviewer for this important point. We agree that it is essential to distinguish between the effects of general overconsumption and those driven by specific dietary components. This question was directly addressed in our study through comparison with multiple additional carbohydrates administered at similarly high doses. Notably, overconsumption of arabinan, a slowly fermentable polysaccharide composed of arabinose, or more inert substances like polyethylene glycol did not promote *S.* Typhimurium colonization or systemic infection (**Fig. 3E–F; Fig. 1G**). In contrast to free arabinose, arabinan induced only mild intestinal acidification, consistent with its slower fermentation kinetics. Similarly, high doses of readily absorbable sugars such as sucrose, glucose, fructose, and galactose did not significantly impair colonization

resistance compared to PBS controls (**Fig. 1F; Supplementary Fig. 1A**). We reason that these sugars are efficiently absorbed in the small intestine and therefore largely unavailable to the gut microbiota, limiting their capacity to drive fermentation-dependent changes in the intestinal environment.

These findings indicate that overconsumption *per se* is not sufficient to disrupt colonization resistance. Rather, the observed effects are specific to certain low-digestible sugars that reach the lower gastrointestinal tract and are rapidly fermented by the microbiota, leading to transient acidification and downstream microbiota and metabolic changes.

In response to the reviewer's comment, we have clarified this point in the Discussion (**Lines 387-392**). We also now explicitly acknowledge the limitation that our study did not constitute an exhaustive screen of dietary nutrients or food combinations, and that additional compounds or mixed dietary contexts may reveal further mechanisms influencing colonization resistance (**Lines 488-496**).

Results II: By focussing on arabinose, the authors observed that rapid fermentation of this sugar acidifies the intestinal environment, which alters the microbiota composition and metabolite profile, including shifts in short-chain fatty acids and elevated amino acids that fuel pathogen growth. Arabinose consumption impacts virulence by broadening tissue tropism, enhancing systemic infection, and selecting for fully virulent, invasive Salmonella through modulation of microbiota–pathogen interactions.

Comment2: Again the question regarding specificity arises: so how far are these effects only the effect of an acidification of the intestinal environment, so have not really to do with LDS?
Please show some convincing data on the specificity of the effect, compare as control with another acidifying food.

RESPONSE:
We thank the reviewer for raising this important point. Our data indicate that acidification is one mechanism by which certain LDSs impair colonization resistance, but not the only LDS-associated mechanism.

We have now included additional data in **Supplemental Fig.6** showing intestinal pH measurements for all LDSs tested in this study. These data clarify the relationship between LDS overconsumption and luminal acidification. In addition to arabinose, overconsumption of lactulose or maltitol also induces intestinal acidification. This supports the idea that chemically distinct, rapidly fermentable LDSs can converge on a shared mechanism involving luminal acidification, microbiota perturbation, and reduced colonization resistance.

However, this effect is not universal across LDSs. Sorbitol and erythritol promoted *S.* Tm infection without detectable intestinal acidification, indicating that acidification alone is not sufficient to explain the observed phenotype. We therefore conclude that LDSs can

impair colonization resistance through both acidification-dependent and acidification-independent mechanisms.

To further assess nonspecific effects of carbohydrate overconsumption, we showed that LDS overconsumption in the absence of infection did not cause overt tissue damage or inflammation (**Supplementary Figure 1A, B**), arguing against host tissue injury as the primary explanation for increased susceptibility.

In response to the reviewer's comment, we have revised the text (**Lines 378-385**) to clarify this distinction and to avoid implying that all LDSs act through one common mechanism and to remind the reader that the LDS may disrupt colonization resistance in a context-dependent fashion (as shown for the case of the $CaCO_3$ neutralization in arabinose overconsuming mice. We now explicitly state that arabinose, lactulose, and maltitol likely act, at least in part, through rapid fermentation and acidification, whereas sorbitol and erythritol appear to involve distinct mechanisms. Elucidating these acidification-independent mechanisms will require dedicated follow-up studies.

Abstract III: These findings reveal how diet can impact the gut microbiota in ways that enhance pathogen colonization and virulence.

Comment 3: Please modify in the light of the revision comments, in the moment one should rather state: "These data show how a strong food stimulus including strong acidification changes the gut intestine. Further studies are necessary to be more specific, for instance to reveal the specific effects of a LDS pulse." The infection data are quite nice, including SPI-1 and SPI-2 involvement and data, but again, they only show how an infection comes about, nothing specific yet (or if I overlooked here something, then stress these specific points, will be interesting to read).

RESPONSE
We thank the reviewer for this comment. As outlined in our responses above, we have addressed the question of specificity by providing additional data and clarifications demonstrating that the observed effects are not due to overconsumption *per se*, but depend on the properties of individual low-digestible sugars.

In light of these revisions, we have modified the abstract as suggested by the reviewer to better reflect both the mechanistic conclusions supported by our data and the specific findings of our study. In particular, we now more clearly describe that arabinose overconsumption leads to intestinal acidification, microbiota perturbation, and metabolite changes and that these changes are associated with enhanced pathogen growth, while pointing out that the full mechanistic details explaining the enhanced growth await further studies and avoiding overgeneralization across all LDSs.

We have also strengthened the presentation of the infection data to highlight the specific insights gained from our model. Specifically, we now emphasize that, unlike in antibiotic-pretreatment models, sustained gut colonization in arabinose-overfed mice requires SPI-1 and SPI-2 virulence functions. This establishes a direct link between virulence factor expression and pathogen fitness in a diet-induced perturbation context,

providing a mechanistic distinction from existing infection models. We also rewrote part of the last paragraph in the introduction to emphasize this point in **Lines 69-72**.

We believe these revisions address the reviewer's concern by clarifying both the scope and specificity of our findings.

Discussion:
--You start the discussion with two very informative reviews: Kreuzer and Hardt, 2020, Woelfel et al., 2024. These also compare what is known for gut microbiota and colonization resistance not only in the mouse model but also what applies to the human and patients.
So please expand this section and convince the reader that you really can add here compared to what is known from the two reviews, particular regarding human resistance to Salmonella infection. Again, it is completely fine if you want in the end just to say "These data show how a strong food stimulus including strong acidification changes the gut intestine in our mouse model. Further studies are necessary to be more specific, for instance to reveal the specific effects of a LDS pulse, and to be more sure how far this can be translated to humans and ultimately, patients." However, the reader should not be left in the dark about this really important point.

--Moreover, as far as I see (but may be you know even better), at least regarding the mouse model this is a rather extensive and detailed study for such a food stimulus and the resulting infection and so you can stress this convincing positive point of your study, too, in the discussion.

RESPONSE
We thank the reviewer for this very helpful suggestion. We agree and have modified the Discussion to more clearly place our findings in the broader context of microbiota-mediated colonization resistance and human infection susceptibility.

To achieve this, we have expanded the opening paragraph of the Discussion (**Lines 338-352)** to better frame the translational relevance of our study. Specifically, we now include discussion that while antibiotics are well-established risk factors for enteric infection, additional susceptibility determinants remain poorly understood, particularly in humans, where studies largely focus on identifying contaminated food sources rather than the contribution of diet itself to infection susceptibility. We also place our findings more explicitly in the context of prior work showing that dietary perturbations can weaken colonization resistance in animal models.

Importantly, we have clarified the specific contribution of our study relative to these prior reviews. While previous work has established that diet can alter microbiota-mediated colonization resistance, our study provides a mechanistic framework showing how acute overconsumption of specific carbohydrates can not only disrupt colonization resistance, but also reshape pathogen–microbiota interactions in ways that enhance pathogen virulence in vivo.

We also explicitly acknowledge that the extent to which these findings translate directly to humans remains to be determined in the last paragraph of the Discussion (**Lines 488-493).**

Minor:
Overall statistics is well done, but only in Figure 2 we learn N = 5 mice per group, please tell the group sizes also for the other figures were appropriate

We thank the reviewer for noting this. Group sizes for all experiments are provided in the raw data/statistics Excel file included in the supplementary materials. To improve clarity and consistency, we have now also added the corresponding group sizes (n values) to the figure legends where appropriate. In doing so, we identified and corrected an error in the labeling of group sizes in **Figure 2** and Supplementary **Figure 2B**; the correct group sizes are **n = 6** for **Figure 2** and **n = 7** for Supplementary **Figure 2B**.

Figure X

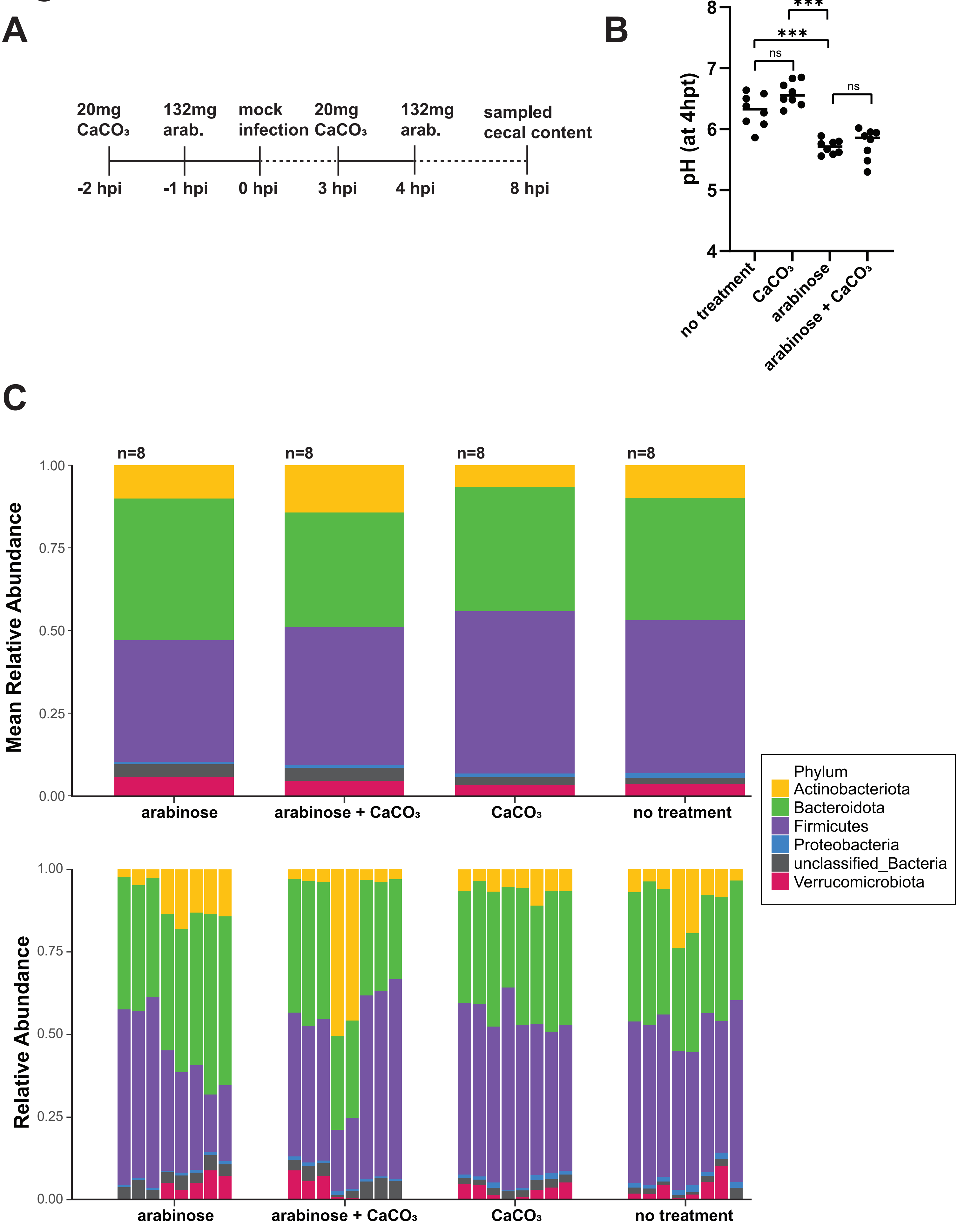

Figure legends on the following page.

D

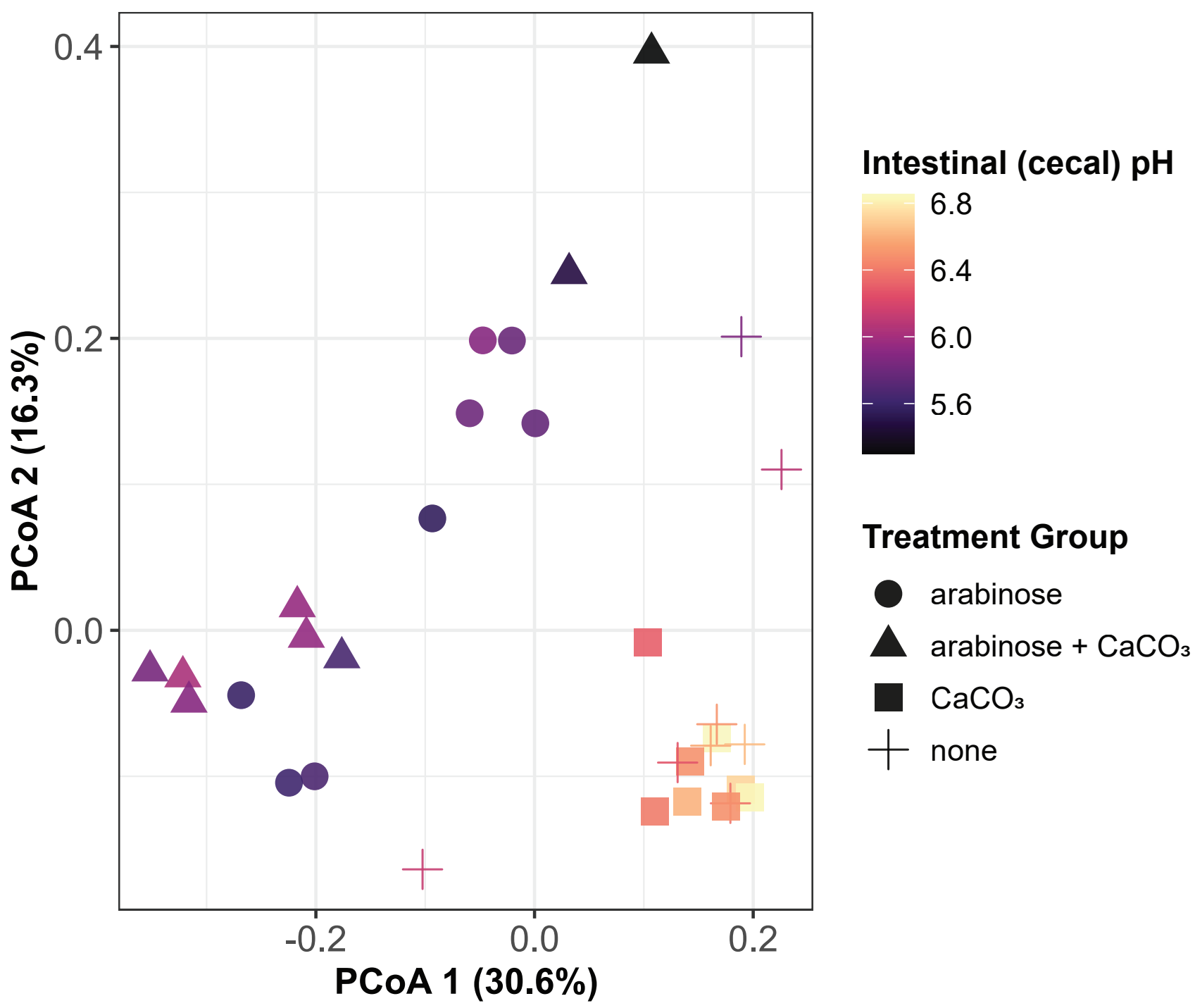

Figure X. Effects of calcium carbonate treatment on arabinose-associated changes in the cecal microbiota.

(A) Experimental scheme.Timing of treatments corresponded to the mock infection schedule. Cecal contents were collected 4 hours after the final treatment.

(B) pH measurements of cecal contents from the indicated treatment groups.

(C) Cecal microbiota composition determined by 16S rRNA gene sequencing. Upper panel: mean relative abundance across mice per group; lower panel: relative abundance in individual mice.

(D) Principal component analysis (PCA) of 16S rRNA gene sequencing data from the indicated groups.

REVIEWERS' COMMENTS

Reviewer #1 (Remarks to the Author):

Major point #1 It is concerning that the authors cannot reproduce their pH neutralization with calcium carbonate, which makes me think that their data in Fig 3G and 3H using calcium carbonate is not reproducible. If this is the case, I don't think it is appropriate nor rigorous for them to publish any data using calcium carbonate that is not reproducible.

RESPONSE
We agree with the reviewer that data that could not be reproduced should not be included. However, the failure to reproduce the effects of calcium carbonate treatment does not exclude a potential role for gut acidification, which is also supported by other findings in our study. We have therefore retained these supporting observations but tempered their interpretation to avoid asserting a causal relationship. Differences observed during the repeat experiments only suggest that the effects of LDS overconsumption and pH neutralization may depend on microbiota composition. The interplay between microbiota composition and gut acidification remains an important avenue for future investigation.

Accordingly, we have removed **Fig. 3G** and **H** and the associated text. We also revised the relevant interpretations and conclusions to avoid causal claims regarding pH and instead describe gut acidification as being associated with reduced colonization resistance.

Changes:
Remove the word "which" in **line 26** of the Abstract

Remove the phrase " leading to" in **line 68** of the Introduction.

Remove **lines 201-211** of the Results, "To determine whether reduced pH causally promoted *S*.Tm growth… suggesting additional factors may contribute" and **Figure 3G** and **3H** and replace with the following text, "*These data suggest an association between gut acidification and reduced colonization resistance.*"

In the Discussion:

Add "may have" to "This pH shift *may have* reversibly altered…" in **line 356**

Remove "which was lost when gut acidification is neutralized by calcium carbonate (**Figure 3H**). These findings suggest that gut acidification can drive transient microbiota dysbiosis that weakens colonization resistance" in **lines 361-363.**

Manor point #2 I still think their data do not directly support their conclusion regarding inflammation. In my opinion, they should use an anti-inflammatory drug and show that reducing inflammation during infection reverts the microbiota composition to those of uninfected arabinose-treated mice if they want to state that inflammation is driving changes in the microbiota at days 3-

4. With their current data, they can only say that Salmonella virulence drives microbiota changes at days 3-4.

We appreciate the reviewer's suggestion. Although anti-inflammatory drugs are available, their effects on host defenses, pathogen burden, and microbiota composition would confound this experiment and prevent inflammation from being isolated as the causal variable. Regardless, we have replaced "pathogen-induced inflammation" with "*Salmonella* virulence" in **line 161**.

Reviewer #2 (Remarks to the Author):

Thank you for your well taken revisions:
1) it is essential to distinguish between the effects of general overconsumption and those driven by specific dietary components. These findings indicate that overconsumption per se is not sufficient to disrupt colonization resistance. In response to the reviewer's comment, we have clarified this point in the Discussion (Lines 387-392). We also now explicitly acknowledge the limitation that our study did not constitute an exhaustive screen of dietary nutrients or food combinations, and that
additional compounds or mixed dietary contexts may reveal further mechanisms
influencing colonization resistance (Lines 488-496). --> well done
2) how far are these effects only the effect of an acidification of the intestinal environment? We have now included additional data in Supplemental Fig.6 showing intestinal pH measurements for all LDSs tested in this study. In response to the reviewer's comment, we have revised the text (Lines 378-385) to clarify this distinction and to avoid implying that all LDSs act through one common mechanism. --> well done
3) we now more clearly describe that arabinose overconsumption leads to intestinal acidification, microbiota perturbation, and metabolite changes and that these changes are associated with enhanced pathogen growth, while pointing out that the full mechanistic details explaining the enhanced growth await further studies and avoiding overgeneralization across all LDSs. --> well done
4) we have expanded the opening paragraph of the Discussion (Lines 338-352) to better frame the translational relevance of our study --> well done
Minor: we identified and corrected an error in the labeling of group sizes in Figure 2 and Supplementary Figure 2B; the correct group sizes are n = 6 for Figure 2 and n = 7 for Supplementary Figure 2B. --> fine
Nothing left, all done!

RESPONSE
We thank the reviewer for their encouraging "well done's"—an assessment we would not presume to make ourselves!
